# Knocking for gold. How long must I? A survey report on international students seeking healthcare in Hungary

**DOI:** 10.3389/fpubh.2025.1624806

**Published:** 2026-01-22

**Authors:** Livia Yawa Like Atiku, Erika Maria Marek

**Affiliations:** 1 Department of Public Health Medicine, University of Pécs Medical School Hungary, Pécs, Hungary; 2 Doctoral School of Clinical Medical Sciences, University of Pécs Medical School Hungary, Pécs, Hungary

**Keywords:** health, access, healthcare, international students, Hungary, wellbeing

## Abstract

**Introduction:**

Migration significantly impacts the health of international students in host countries. It has been reported in several studies that one of the main challenges for international students is gaining access to healthcare services. This study examined the experiences of international students in Hungarian universities, focusing on their self-reported health status, access to healthcare services, adequacy of services rendered, cultural sensitivity, and quality of care.

**Method:**

This cross-sectional study gathered responses from 476 students across major universities. However, only 440 were strictly analysed, and 436 in situations where questions under certain parameters were left unanswered.

**Results:**

Using a structured online survey and CART analysis with chi-squared test as goodness of fit, we examined socio demographic influences on healthcare access and the availability of culturally competent care. Key findings revealed notable health challenges, with 28.3% of students reporting health deterioration after migrating to Hungary. Approximately 63% arrived with limited or no prior information on the healthcare system, and only 35.9% had a full understanding of their entitlements to comprehensive healthcare.

**Conclusion:**

Despite the Stipendium Hungaricum scholarship’s insurance coverage, 30.9% of students incurred some out-of-pocket expenses, and 4.6% paid entirely for public healthcare. Trust issues also surfaced, with 36.7% placing more confidence in home-country healthcare service providers compared to 20.6% for Hungarian healthcare service providers. Issues of discrimination were implied from the data but not supported in explicit statements. These findings underscore critical policy needs, including enhanced intercultural competence, better language support, and expanded mental health services.

## Introduction

In a joint report of the World Health Organisation (WHO) and the World Bank on global monitoring in the light of Universal Health Coverage (UHC), it was revealed that progress towards providing people everywhere with quality, affordable, and accessible healthcare has suffered a terrifying stagnation (1). Captured in a precise and succinct statement of the Director-General of WHO was the concern about the rippling effect of leaving some people behind the bridge to universal health coverage: “The fact that so many people cannot benefit from affordable, quality, essential health services not only puts their own health at risk, it also puts the stability of communities, societies and economies at risk, We urgently need stronger political will, more aggressive investments in health, and a decisive shift to transform health systems based on primary healthcare” (2).

In European countries like Bulgaria and Germany, beneficiaries’ claims to healthcare were primarily restricted to medical or surgical exigency care, but in recent times, have been upgraded to comprehensive coverage with the rollout of new regulations (3). Migrant health has been a central issue for many corporate bodies of global interest (4). The World Health Organisation creates the awareness that migrant-inclusive health systems improve public and global health outcomes for all; thus, preserving lives, reducing disease burdens, and death rates (5). With this mindset, efforts were marshalled towards ensuring displaced persons from disturbed geographical locations, such as Ukraine, acquired a new status that placed them at par with documented asylum seekers who have been integrated into the social security system of Germany (6). This wave of humane assistance was replicated by Croatia, Hungary, and Sweden, granting entitlements in specific, smaller groups of beneficiaries to the full state benefits coffers, including (unaccompanied) children and adolescents below the age of 18 and (pregnant) women. Hungary typically offered general health assessments to identify those who required immediate healthcare, some of which were compulsory on arrival (7).

The Stipendium Hungaricum scholarship programme paved the way for over 11,000 international students to benefit from free tuition, comprehensive health insurance, regular stipends to cushion the cost of living, and accommodation allowances for all levels of higher education (8). This contributed to making Hungary an attractive destination for these huge numbers (350,000^+^) (9), drawing international students from across the globe, with dominance in the field of medicine due to the introduction of English-taught programmes specifically tailored for international medical students. In the realisation of these kinds of figures, global consultations on migration to accelerate deliberations on the role of scientific research in supporting evidence-based health responses associated with migration were embraced with speed (7). One category of such migrants is foreign students (10). International students are driven to study in their host nations for many reasons. Notably, they are enthusiastic about having new experiences of discerning and performing in their study discipline. Furthermore, studying abroad increases career opportunities due to the attainment of improved expertise for prospective economic engagement in their native country or other nations (11). The outbreak of SARS-CoV-2, despite its contagious nature, did not dim the chances of both native universities and prospective international students. Rather, measures were put in place to minimise the risks of cross-border spread through criteria defined by state protocols (8). Impacts of the pandemic rippled in many unfathomable dimensions. Students arriving had to complete forms at immigration borders detailing whether they carried signs and symptoms of the respiratory infection. Questions regarding the polymerase chain reaction (PCR) test within a time frame of 48 h were also confirmed at the immigration checkpoints before being allowed entry into the country (12). Marked changes occurred during the period under review, including an unprecedented drop in the number of international students admitted to programmes in Hungary. It was reported that a significant 15% dip was recorded from the previous year, resulting in the enrolment of only 35,000 in 2020. The disruption was also felt in the call for vaccination at approved hospital centres, with restrictions on travel activities compounding individual health risks and financial strain on healthcare resources (13). The pandemic narrowed international students in their movement and meetings as lectures became an online engagement, compounding their challenges with reduced chances of socialising with others in activities of fitness or leisure that nurture friendships in cultural diversity. The prevailing circumstances confronting migrants from the start of their journey to the time of settling in their new environment potentially expose them to life-threatening dangers that predispose them to physical and mental disorders (14). Some of these situations include, but are not limited to, unequal access to healthcare services, susceptibilities related to migrant status (asylum seekers, refugees, foreign workers, or international students), side-lining and exploitation due to austere immigration checks, and policies guiding employment and other socio-economic factors. International students are reported to have been treated to some extent with poor services in host countries by native caregivers (15). Often, common anti-migrant views held by members of a society also take a toll on these vulnerable migrants. Thus, with their influence on social life, they become the determinants of the migrant’s health. Fixing this conglomerate of factors catalyses migrant’s acculturation and integration for progressive development (16).

While global discussions on control measures for the pandemic were ongoing, some fresh students were battling with acculturation issues, including nutrition (17), and others had their graduation in limbo (18). Evacuation of native students from hostels to create the recommended distance allowance for foreign students became crucial. Amidst these difficulties was the yoke of unemployment hanging on the neck of international students, both for personal survival and remittance to families back home. The transition phase recorded outbursts of racism and xenophobic sentiments, which overwhelmed the mental health of students. Students of certain nationalities were said to have been tagged as carriers of the viral infection (18).

Feelings of isolation, loneliness, and homesickness were widespread among students, opening the floodgates for mental health problems to creep in. Complaints of anxiety and depression were dominating, while some struggled post-traumatically. Many students were reporting ill, and deteriorating health was common, predominantly from the quarters of undergraduate students (19). The mental health of international students suffered during the COVID-19 pandemic due to various factors arising from enforced restrictions, evident as financial strains, academic hurdles, and inadequate support systems (20).

This situation became the litmus paper that determined whether the health system of Hungary had the required shock absorbers to remain resilient under the pressure of an unforeseen pandemic. Unfortunately, many countries are confronted with the challenge to strengthen their health systems by improving the quality and accessibility of healthcare while streamlining the cost of health expenditure (21). Based on Ashcroft’s principle of support captured in a statement, “Many issues in professional ethics concern failures to respect a person’s autonomy, ranging from manipulative under-disclosure of pertinent information to non-recognition of a refusal of medical interventions.,” we embarked on this study to find out more (22).

Health spending continues to grow amidst the advent of novel trends in technology and demography as crucial elements for policymaking for healthcare. Consumers have become increasingly aware not only of their rights but their concept of quality healthcare. European healthcare systems usually provide coverage of most healthcare costs for their entire populace, with systems structured to control healthcare spending at the national level (23).

Hungary offers its populace a relatively affordable health insurance package as compared to many of its other European member countries. Generally, Stipendium Hungaricum scholarship students are covered by a comprehensive national health insurance plan that exempts such students from making out-of-pocket payments for services except for prescribed drugs. The national health insurance package absorbs the cost of preventive screening tests, outpatient care, surgeries, and a 24-hour call centre for consultation, among others (24). The state of Hungary heavily subsidises public health services to lift the economic burden off patients. This goodwill is available to international students in an active semester and within the duration of their habitual stay in Hungary as stipulated in the operational regulations of the Stipendium Hungaricum scholarship programme. The services of private entities are also available with a concession for foreign students at affordable rates.

### The significance of research in transforming society

Crafting a space in the field of research for an advancement in human health is a gallant step in leaving no one behind. Magnifying the usual and common movement of people through a humanitarian lens showcased a necessary spot for global health engagement. Thus, sculpting a giant step towards softening the hard, crusty grounds of a million ages in neglect to merit the ardent attention of world health advocates in order to shift the culture from research to research and innovation (25). Migration health has provoked an interest in varied geographical distributions, so that scientific research and the segregation of migrant groups for an understanding of their distinct health needs demand appropriate interventions to drive the agenda for accurate service delivery in different migrant populations. Research scholars and other significant stakeholders have solicited quality research works on migration health on both the domestic and international fronts to propel SDG-3 into realisation (26). International students in Hungary were captured in a study together with domestic students with respect to the statistics and economic gains made within definite time periods, in which Vincze and Bács (27) made an important observation on international students in institutions of higher learning in Hungary. Their study hoisted the flag of Hungary for its booming non-alcoholic beverage industry that introduced some vibrancy into the nation’s economy following the high demand for cocoa and coffee in breakfast and snack packs in its higher learning institutions, but bemoaned the steep decline in the number of native students in their various institutions with each passing year (27). Other researchers have attributed the challenges of international students in Hungary to acculturation stress. One of such cross-sectional studies on foreign students in their new environment blew the whistle on the failing mental and general health among international students due to poor adjustment strategies. The authors believed foreign students could free themselves from all social entanglements if they allowed themselves to be immersed in the Hungarian culture (28). Onosu (29) supported and validated that finding after measuring the impact of cultural immersion experience on the identity transformation process by resonating its significance on the formation, creation, and development of an individual’s persona.

## Materials and methods

### Study design

A cross-sectional design was adopted for an online survey. The authors conducted the study to gather data and identify issues affecting the health and general wellbeing of international students in Hungary using a standardised questionnaire.

### Research setting

Universities in Hungary offer a supportive and affordable environment for international students, characterised by low tuition costs, a range of English-taught programmes, readily available dormitories, and vibrant student life. The country provides a safe and welcoming environment with opportunities for integration and support, such as demonstrated in the Stipendium Hungaricum scholarship and mentor programmes. Housing, safety, and modernity are prioritised. Investments in higher education have resulted in strong academic programmes and a globally recognised educational system. The country, in general, is known for its historical setting, safe environment, and diverse student life. Universities in Hungary provide a vibrant student life with accessible cinemas, bars, restaurants, and shopping options. Students also engage in sports and participate in exchange programmes. Most universities in the country are bio-friendly and have been recognised by world bodies for their green environment. The country boasts of a rich history and culture, with architectural landmarks and cultural events, including the UNESCO World Heritage site.

### Research tool development

Our approach to maintaining methodological clarity focused on a clear questionnaire design, rigorous validation, and appropriate participant selection. A careful question construction, pre-testing using established scales, and the employment of statistical analysis for validation were stages observed. Appropriate participant selection criteria, e.g. representativeness of the sample and efforts at addressing potential biases were of prime concern.

In designing the questionnaire, questions were crafted in clear, unambiguous language and without jargons or technical words for respondents to easily understand. With the use of multiple-choice questions and the Likert scale, responses were formatted to align with the research questions and allow respondents to express their opinions accurately. Questions were logically stringed from simple to progressively more difficult and complex ones. They were also from general knowledge to specific knowledge, ensuring a smooth flow.

The questionnaire was pre-tested with a few people to identify any ambiguities or technical issues. Reverse-worded items were used to detect inconsistencies in responses and to assess the validity of the questionnaire. Validation of established scales (Likert, numerical, descriptive, and verbal scales) from existing research was carried out to ensure reliability and validity. The validation process continued into pilot testing our questionnaire to refine the questionnaire and identify potential issues before the main study. With Cronbach’s alpha, we were able to assess the internal consistency of the questionnaire and factor analysis to examine the underlying structure of the questionnaire and its validity. We triangulated the survey results with other data sources (qualitative and quantitative) for insights to confirm the validity of the findings. Different theoretical frameworks were used to analyse the survey data.

### Inclusion criteria

International students who were active at the time of data collection were included. An active student is the student who has registered for the semester and is actively participating in lectures and other curricular activities.

### Exclusion criteria

Students who had a passive status in the active semester of data collection and were absent from lectures and other curricular activities were excluded.

### Sampling and sampling technique

Respondents in this survey were international students in universities in Hungary. Using an online survey, everyone who received the questionnaire had a chance of being selected at random. Steps to mitigating potential biases of any form were considered. An email invitation to participate in the survey was sent to international students through the university’s portal, Neptun. The email sought the consent of international students and their interest in responding to the questionnaire.

A purposeful empirical survey was carried out in Hungary between May and August 2021. An anonymous online survey questionnaire was sent to the international students’ community by email from the University of Pécs portal, Neptun. A total of 476 responses were received at the end of data collection, but 440 were finally analysed after data purification. The difference of 36 was excluded from the analysis of some variables due to reasons related to incomplete questionnaire responses and visible evidence of illogical responses.

### Data collection method

Data were collected from several international students, including Stipendium Hungaricum scholarship beneficiaries. An anonymous online survey questionnaire was sent to students in Hungarian universities in no specific order or preference, with responses from predominantly 4 universities. There were 63 questions in total for the questionnaire, two of which were open-ended and the rest, closed-ended. There were questions pinned to the Likert scale to allow respondents to indicate their opinions, attitudes, or feelings about their general and mental health wellbeing since arriving in Hungary for academic purposes. The questions were in three main categories seeking information on their basic demographics, self-assessed health status, health habits and health-seeking behaviors, and expectations on healthcare in Hungary. Each closed-ended question had options from which respondents could choose, depending on their reality or choice of preference. There was also available among these, the option to withhold themselves from answering a particular question. Some areas that questions covered included their self-assessed health status, health service availability, accessibility, affordability, acceptability, and quality.

### Data analysis

Descriptive statistics (e.g., response frequencies, percentages, and proportions) were used to summarise variables. Chi-square goodness of fit test for independence was used to investigate the significant differences among the studied parameters and socio-demographic variables. The chi-square analysis in this study was conducted as a goodness-of-fit test, comparing observed frequencies to expected frequencies based on a hypothesised distribution. This involved categorical data, not continuous variables.

To enhance interpretability, we included proportions alongside *p*-values to highlight practical significance. A significant result (*p* < 0.05) indicates a meaningful difference between at least the smallest and largest observed frequencies. The degrees of freedom, however, have been added to all tables.

CART algorithms were employed to assess the relationship between some responses and the socio-demographic variables of participants. Misclassification cost was used to select the most appropriate tree. Finally, *p*-values less than 0.05 (significance at 95% CI level) were considered statistically significant. Data were analysed using SPSS software (Windows version 26).

We used Classification and Regression Tree (CART) models instead of traditional regression methods for several important reasons.

### Sample size justification

With approximately 436 student responses, the sample size was sufficient for tree-based modeling. CART does not require very large datasets to generate reliable splits, especially when the goal is to identify patterns and segment populations rather than estimate population-level coefficients.

### Handling of non-linearity and interactions

CART is well-suited for capturing complex, non-linear relationships between variables without requiring pre-specification. In contrast, traditional regression assumes linear relationships and requires explicit modeling of interaction terms, which may lead to model misspecification if relationships are complex.

### Interpretability

CART models produce intuitive decision trees that are easy to interpret and communicate, especially for applied audiences. This is particularly beneficial in survey research where stakeholder understanding is important.

### Suitability for mixed data types

The dataset included a combination of categorical and continuous variables. CART naturally handles both types of data without requiring transformation or dummy coding, unlike traditional regression.

We have included a visual representation of the CART tree (Figures 1–8) to aid in interpretation and to demonstrate the model’s segmentation logic. The figure highlights the key predictors and decision paths that best explain the variation in survey responses.

**Figure 1:**
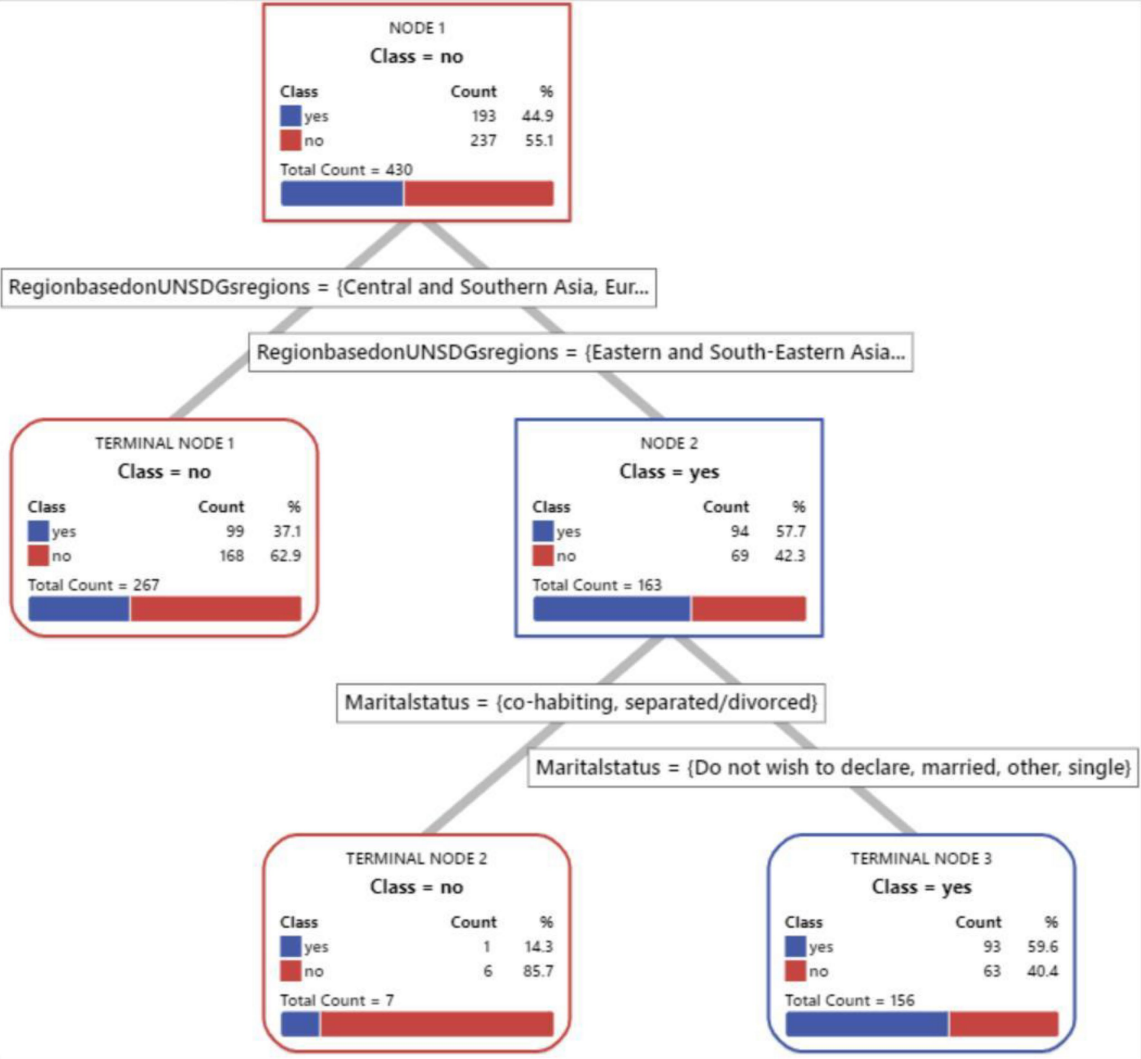
CART algorithm for worry about corona viruses.

**Figure 2:**
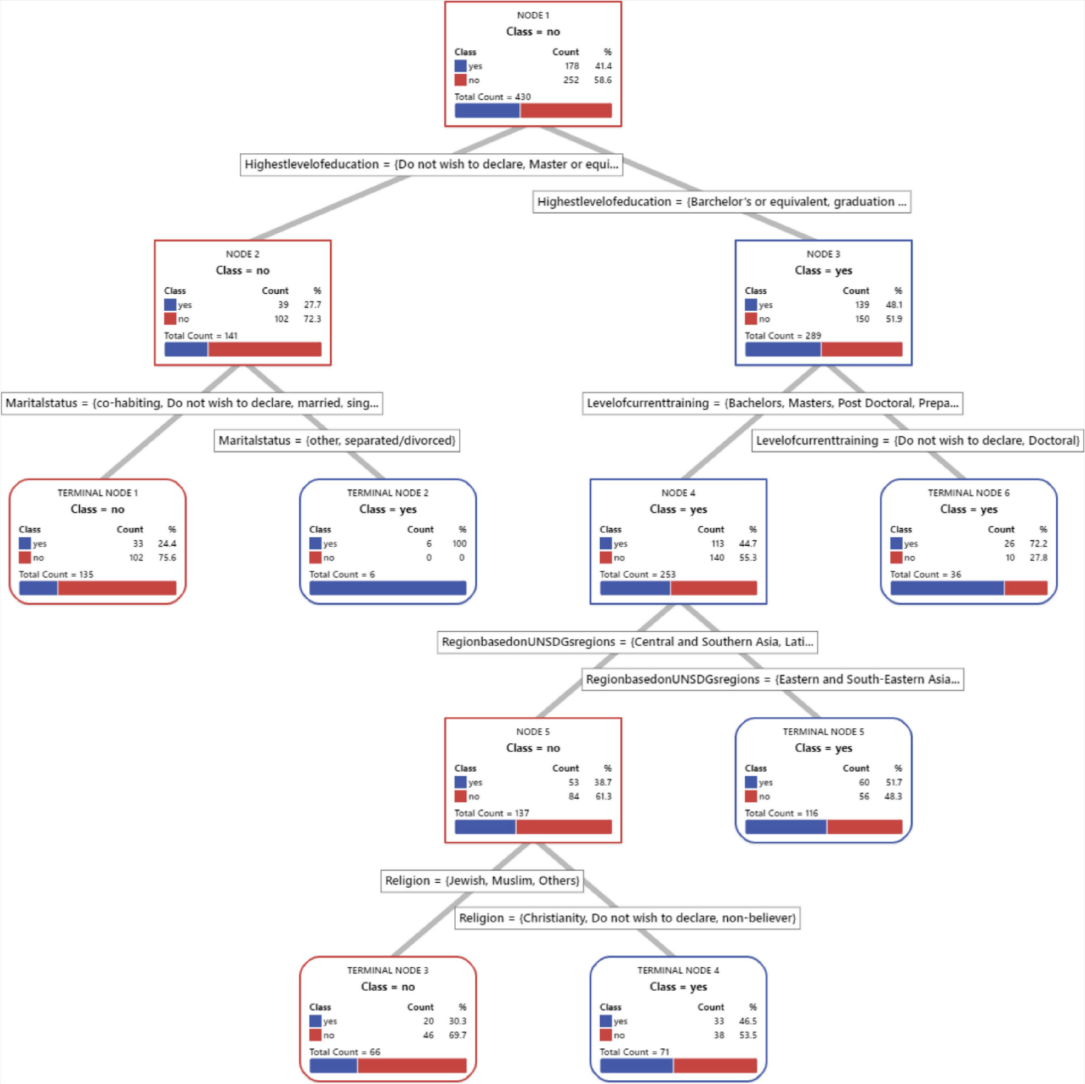
CART algorithm for worry about mental health issues.

**Figure 3:**
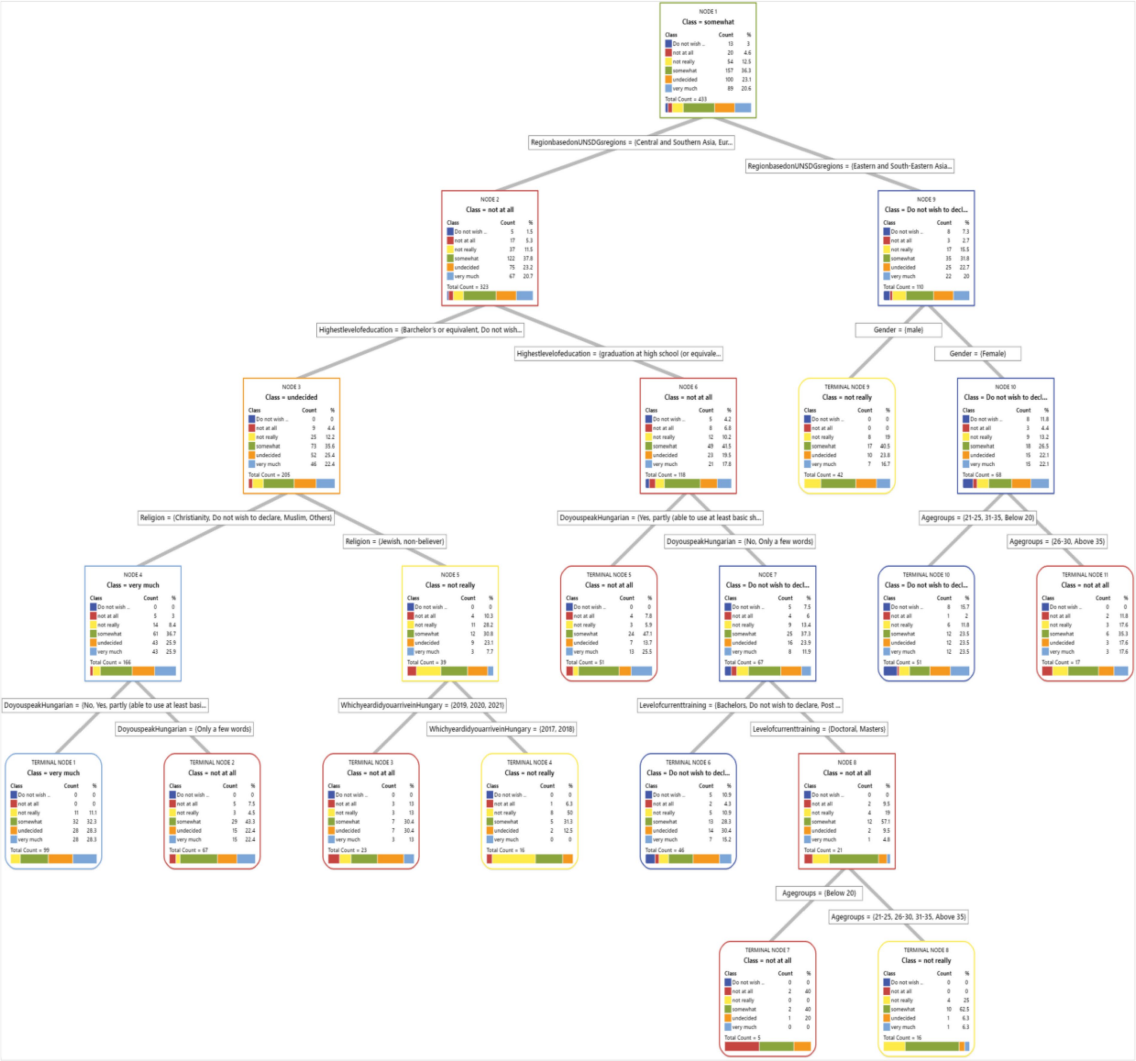
CART algorithm for trust for healthcare professionals in Hungary.

**Figure 4:**
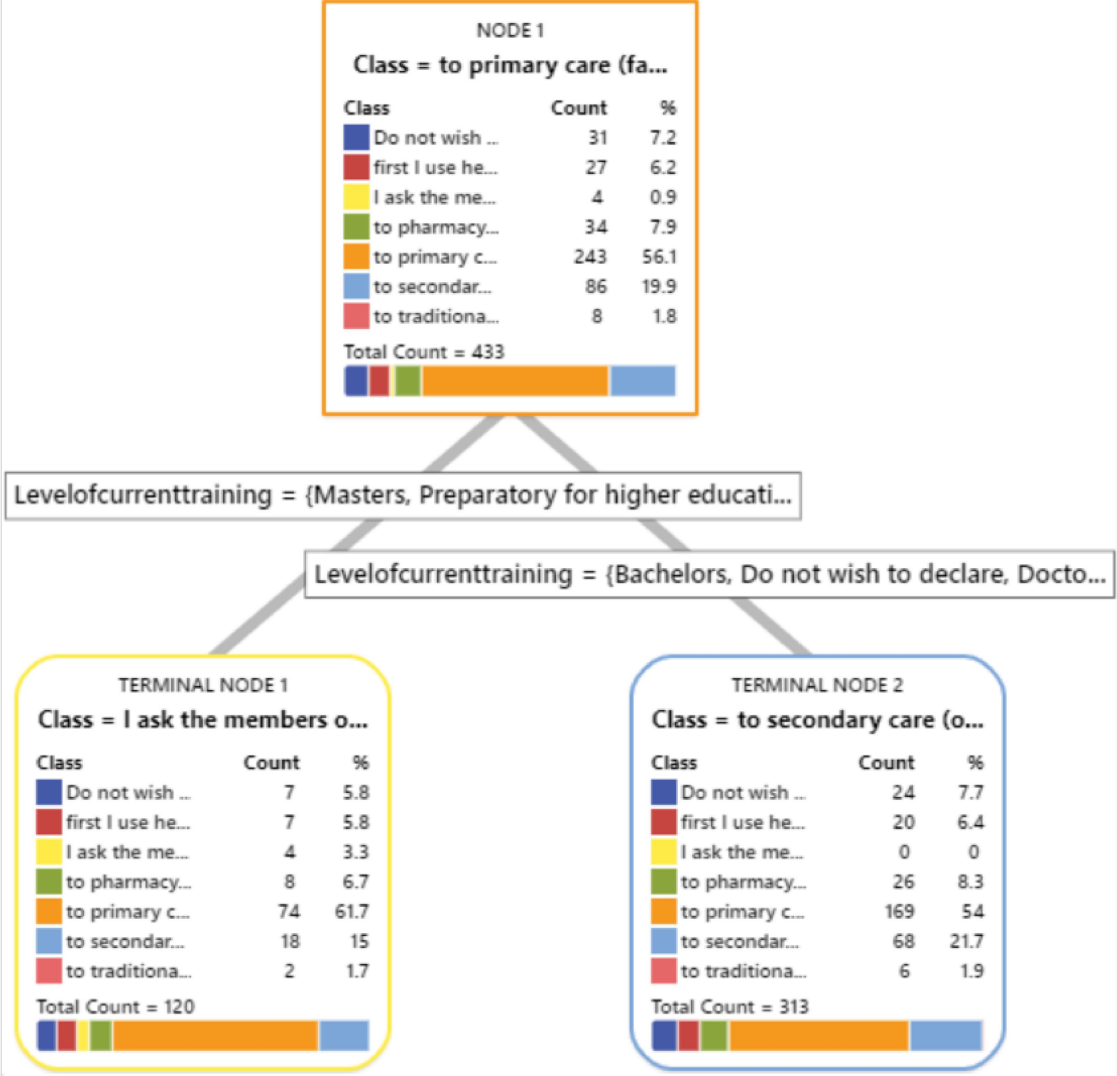
CART algorithm and first preference for healthcare services when needed.

**Figure 5:**
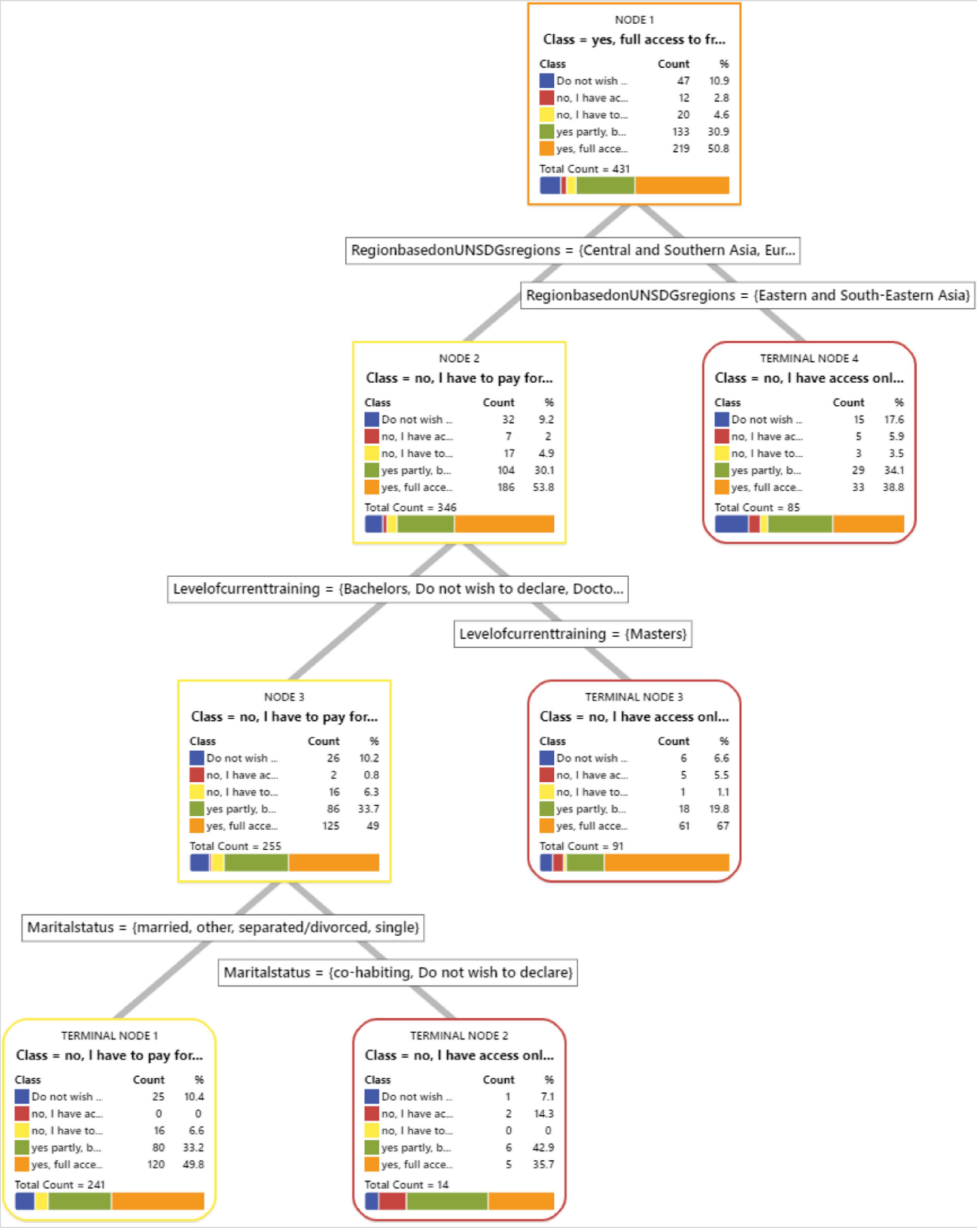
CART algorithms and access to healthcare services in Hungary.

**Figure 6:**
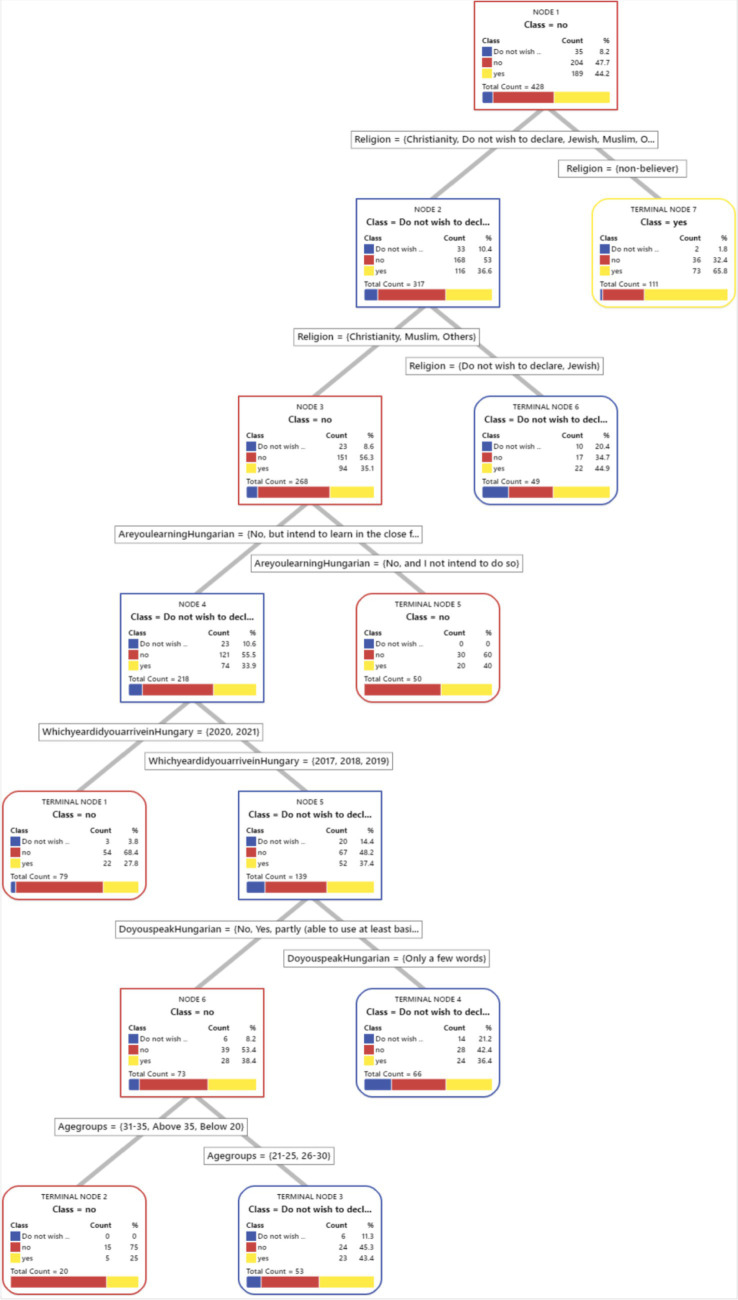
CART algorithm and the need for mental health counselling in Hungary.

**Figure 7:**
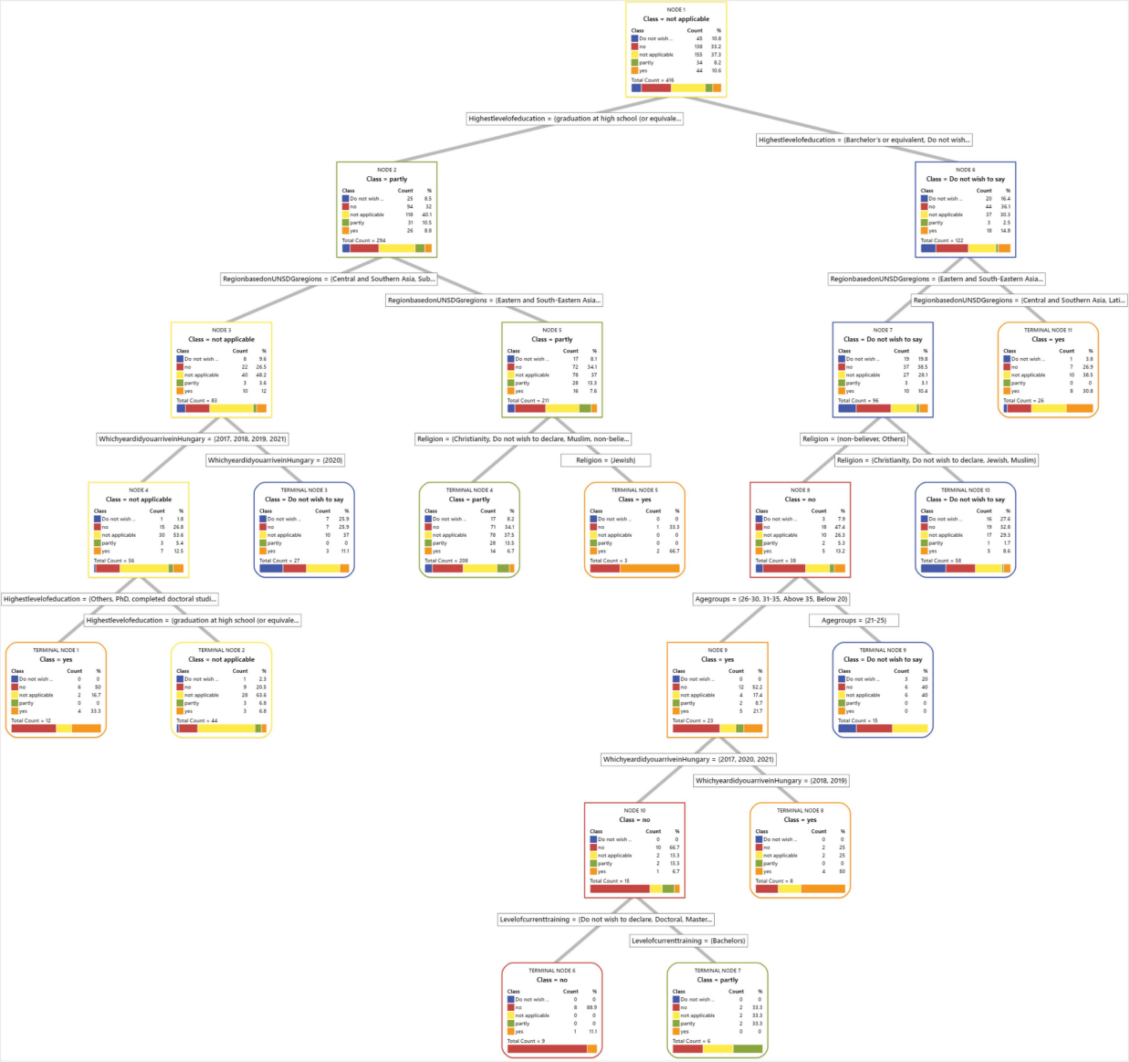
CART algorithm and getting the necessary mental healthcare.

**Figure 8:**
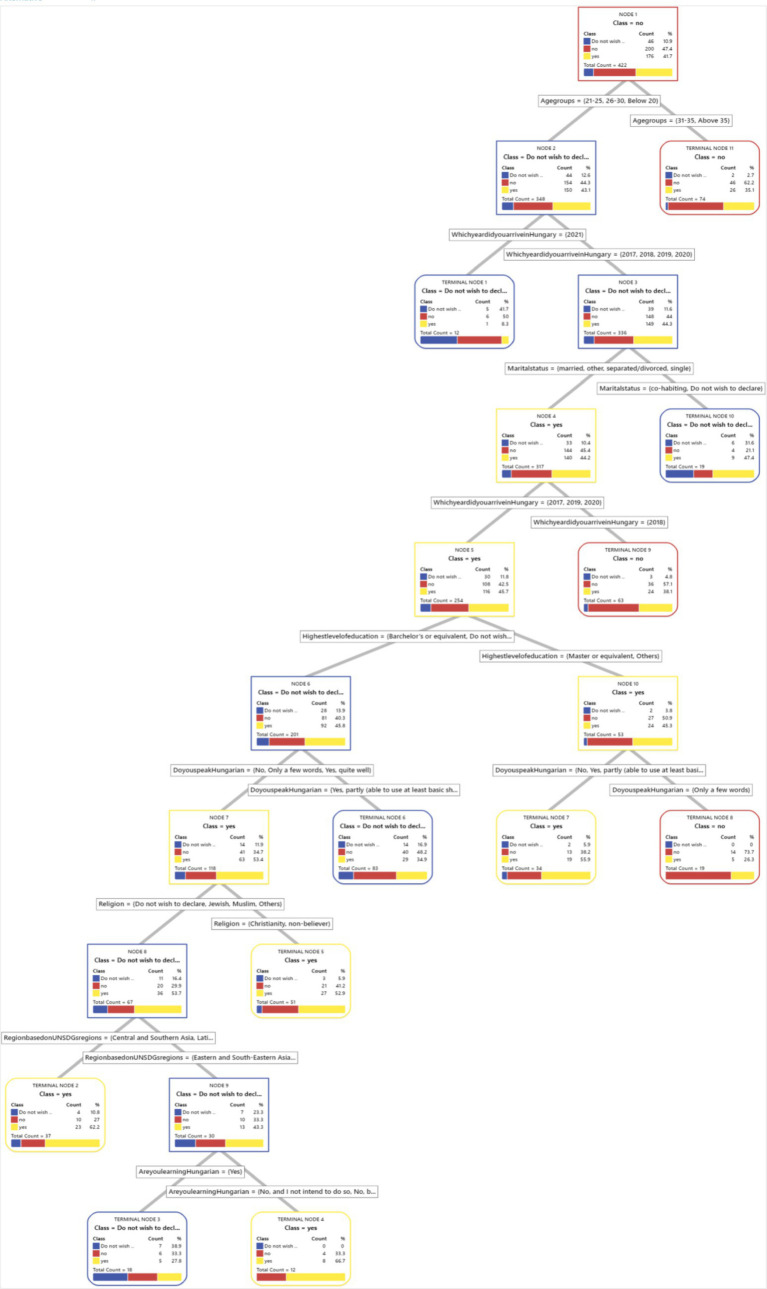
CART models and difficulties in accessing healthcare generally in Hungary.

### Ethical considerations

The study was conducted in accordance with the Helsinki Declaration and was approved by the Institutional Scientific Research Committee of the University of Pecs (approval no: PTE/61924/2021) and the Hungarian Medical Research Council (ethical approval no: BM/26490-1). Protection of data privacy and confidentiality, as required in ethical considerations for scientific research, was followed by granting anonymity in reporting findings as well as limiting access to survey data to only relevant public health specialists and statisticians. Clear and transparent information about the purpose of the research and how the data will be used was given in the mail that disseminated the questionnaire. In conclusion, we adhered to all relevant ethical guidelines and regulations for conducting online surveys.

## Results

### Sociodemographic characteristics

The sociodemographic information of respondents is summarised in Table 1. The proportion of women (53.4%) in the study exceeded men (46.6%). Most respondents, comprising 42.5%, fell within the age group of 21–25 years, while only 5.2% were above 35 years old. The highest proportion of international students came from the Northern Africa and Western Asia region, accounting for 23.9% of respondents. Following closely were students from Eastern and Southeastern Asia, constituting 19.3%. In contrast, Latin America and the Caribbean, as well as Sub-Saharan Africa, had the lowest representation, with proportions of 5.2 and 8.9%, respectively. While Muslims dominated the study with a count of 124 respondents representing 28.2%, Jews were the least represented with a count of just 4, which was less than 1% of the total number of respondents. There were 36.6, 26.4, and 23.6% students with high school, bachelor’s, and master’s qualifications, respectively. Only 6.6% of the respondents had attained their PhD qualification. As a result, the majority of the students surveyed were currently pursuing their bachelor’s, master’s, or PhD degrees with respective proportions of 39.8, 26.6, and 29.3%.

**Table 1:** Sociodemographic characteristics of the study participants.

Variables	Frequency	Proportion (%)	Chi-squared critical value	*p*-value
Gender				
Female	235	53.4	2.05	0.153
Male	205	46.6		
Age groups				
Below 20	80	18.2	176	<0.001**
21–25	187	42.5		
26–30	98	22.3		
31–35	52	11.8		
Above 35	23	5.2		
Region based on UN SDGs regions				
Do not apply	54	12.3	72.7	<0.001**
Europe and Northern America	71	16.1		
Northern Africa and Western Asia	105	23.9		
Sub-Saharan Africa	39	8.9		
Central and Southern Asia	63	14.3		
Latin America and the Caribbean	23	5.2		
Eastern and South-Eastern Asia	85	19.3		
Religion				
Christianity	92	20.9	142	<0.001**
Non-believer	115	26.1		
Muslim	124	28.2		
Jewish	4	0.9		
Others	59	13.4		
Do not wish to declare	46	10.5		
Marital status				
Single	350	79.5	1,266	<0.001**
Co-habiting	15	3.4		
Married	45	10.2		
Separated/divorced	4	0.9		
Other	17	3.9		
Do not wish to declare	9	2		
Number of children				
Have no child	395	89.8	1,340	<0.001**
Have 1 child	13	3		
Have 2 children	6	1.4		
Have 3 or more children	7	1.6		
Do not wish to declare	19	4.3		
Highest level of education				
Graduation from high school (or equivalent)	161	36.6	262	<0.001**
Bachelor’s or equivalent	116	26.4		
Master’s or equivalent	104	23.6		
PhD, completed doctoral studies	29	6.6		
Others	18	4.1		
Do not wish to declare	12	2.7		
Level of current training				
Preparatory for higher education admission	5	1.1	394	<0.001**
Bachelors	175	39.8		
Masters	117	26.6		
Doctoral	129	29.3		
Post Doctoral	1	0.2		
Do not wish to declare	13	3		

### Self-health assessment of participants

International students studying in Hungary were asked to self-assess their health status, and the result is presented in Table 2. Before leaving their home countries, most of the students claimed their health status was very good, with a proportion of 56.6%. This was significantly higher than (*p* < 0.05) those students who had bad health (0.7%) before moving to Hungary. A similar observation was made when the students first arrived in Hungary, where the significant majority (47.5%) again indicated a very good health status, with only 2.5% indicating a poor health upon arrival. When they were asked to rate their health status in the recent period, the proportion of students with very good health status reduced to 36.2%, whereas those with poor health increased to 3%. This led 28.3% of respondents to admit that their health status has slightly deteriorated since they arrived in Hungary. In contrast, a significant 43.7% indicated that their health status had not changed since they arrived in Hungary. Coronavirus infection and mental health problems were the top health problems respondents were worried about at the time of this study. While 44.9% were concerned about coronavirus infections, 41.4% worried about mental health issues. These proportions were significantly higher (*p* < 0.05) than those of respondents who were concerned about acute infectious diseases (6.3%), chronic infectious diseases (3.7%), chronic non-infectious diseases (8.8%), or sexual and reproductive problems (7.7%). The majority of respondents (67.4%) had no known emerging health problems. Conversely, 22.5% had mental health problems as their emerging health problem of concern. Chronic health problems were not of concern to most of the respondents, as 75.5% said they do not have any chronic health problems. Accordingly, 83.8% of respondents do not have to take any medications regularly. For those 13.7% who responded that they take medications regularly, 20.3% had stopped taking their medications because they claim they have finished and are no more being prescribed in Hungary. A significant 33.8% of respondents attributed their decision to stop their medication to other reasons aside from their personal decision to stop taking the medication (9.4%), the medication not being available in Hungary (9.4%), or no specialist found yet in Hungary (27.0%). Dental problems were of no concern for international students in Hungary, as the significant majority (81.5%) responded ‘no’ when asked if they had this health issue.

**Table 2:** Self-health assessment of participants.

Characteristics	Frequency	Proportion (%)	Chi-square (χ^2^)	*p*-value (α ≤ 0.05)
How would you rate your health (before leaving home country)				
Very good	246	56.6	526	<0.001
Good	147	33.8		
Fair	39	9		
Bad	3	0.7		
How would you rate your health (When you first arrived in Hungary)				
Very good	187	47.5	337	<0.001
Good	141	35.8		
Fair	56	14.2		
Bad	10	2.5		
How would you rate your health (Recently)				
Very good	147	36.2	199	<0.001
Good	146	36		
Fair	101	24.9		
Bad	12	3		
Has your health status changed since arrival in Hungary				
It improved significantly	27	6.2	225	<0.001
It improved slightly	69	15.9		
It has not changed at all	190	43.7		
It deteriorated slightly	123	28.3		
It deteriorated significantly	26	6		
What health problems have you recently been worried about				
Coronavirus infection (SARS-COV-2), Covid-19	193	44.9	N/A	N/A
Acute infectious disease	27	6.3		
Chronic, infectious disease (i.e., hepatitis and HIV)	16	3.7		
Chronic, non-infectious diseases	38	8.8		
Dental problems	124	28.8		
Sexual and reproductive health issues	33	7.7		
Mental health issues	178	41.4		
Others	77	17.9		
Do not wish to declare	64	14.9		
Do you have any newly emerging health problems				
Yes, acute physical health problem	48	11.1	N/A	N/A
Yes, chronic physical health problem	21	4.9		
Yes, psychological/mental health problem	97	22.5		
No, nothing	291	67.4		
Do you have any chronic health problems				
Yes	44	10.1	597	<0.001
No	330	75.5		
Maybe	39	8.9		
Do not wish to declare	24	5.5		
Do you (have to) take any medication regularly				
Yes	60	13.7	508	<0.001
No	366	83.8		
I should have taken, but I do not take	11	2.5		
Why do not you take your medication if you should have				
Finished and no more prescribed	15	20.3	17.1	0.002
Have decided to stop	7	9.4		
Medication not available in Hungary	7	9.4		
No specialist has been found yet in Hungary	20	27		
Other reasons	25	33.8		
Do you have any longstanding dental problems				
Yes	81	18.5	173	<0.001
No	356	81.5		

α = statistically significant.

### Difficulty in accessing and utilising healthcare services

Respondents were asked about how difficult it is to access healthcare in Hungary, and their responses are summarised in Table 3. The majority indicated that they will trust health professionals from their home countries more than those from Hungary. While 36.7% rated their trust in health professionals from their home country as ‘very much’, only 20.6% gave a similar rating to health professionals in Hungary. The significant (*p* < 0.05) majority (36.3%), however, stated that they somewhat trust health professionals in Hungary. When the need for health services arises, most of the respondents said they would rather contact their primary care providers, with a significant (*p* < 0.05) proportion of 56.1% rather than contacting their religious community for advice (0.9%) or contacting a traditional healer (1.8%). Surprisingly, 63% of respondents did not have any information about the healthcare system in Hungary before their arrival. This proportion significantly exceeded the 12.9% who claimed they had such information before they arrived in Hungary.

**Table 3:** Difficulty in accessing healthcare.

Characteristics	Frequency	Proportion (%)	Chi-square (χ^2^)	*p*-value (α ≤ 0.05)
How much trust do you have in health professionals in your home country				
Very much	159	36.7	272	<0.001
Somewhat	136	31.4		
Not really	47	10.9		
Not at all	8	1.8		
Undecided	68	15.7		
Do not wish to declare	15	3.5		
How much trust do you have in health professionals in Hungary				
Very much	89	20.6	205	<0.001
Somewhat	157	36.3		
Not really	54	12.5		
Not at all	20	4.6		
Undecided	100	23.1		
Do not wish to declare	13	3		
Where do you prefer to go first for health services when you need them				
Primary care (family doctor/GP) providers	243	56.1	846	<0.001
Secondary care (outpatient clinic or hospital) providers	86	19.9		
Pharmacy for over-the-counter (OTC) medications	34	7.9		
First, I use herbal medicine myself or within the family	27	6.2		
Traditional/alternative healers	8	1.8		
I ask the members of my religious community for advice/care	4	0.9		
Do not wish to declare	31	7.2		
Did you have information about the healthcare system in Hungary before your arrival				
Yes	56	12.9	180	<0.001
No	273	63		
Partly	104	24		
Did you get any information about your entitlements to health services in Hungary				
Yes	155	35.9	1.43	0.489
No	142	32.9		
Partly	135	31.3		
How did you get the information				
Online, from the Hungarian governmental website	70	16.8	N/A	N/A
Online, Tempus Public Foundation/Stipendium Hungaricum website	190	45.7		
Online, the University administration website	174	41.8		
Online, from another website	61	14.7		
Informally, friends or family	166	39.9		
From brochures	27	6.5		
On-site, at the university administration office	92	22.1		
From another source	58	13.9		
Do you have access to healthcare services in Hungary				
Yes, full access to free public health services	219	50.8	518	<0.001
Yes, partly, but I have to pay for certain public health services	133	30.9		
No, I have to pay for all public healthcare services	20	4.6		
No, I have access only to private healthcare	12	2.8		
Do not wish to declare	47	10.9		
Have you been presented with any health problems since your arrival in Hungary				
Yes	232	54.1	167	<0.001
No	176	41		
Do not wish to declare	21	4.9		
If yes, did you get the necessary care				
Yes, always	102	27	36.4	<0.001
Yes, in most cases, but not always	97	25.7		
Rather not, only in some cases	42	11.1		
No, never	55	14.6		
Do not wish to declare	82	21.7		
What kind of medical care have you taken since you arrived in Hungary				
Preliminary health check/aptitude test upon arrival at the university	153	38.1	N/A	N/A
Primary care/GP services with acute problems (i.e., infections)	132	32.8		
Primary care/GP services with chronic conditions	60	14.9		
I visited a dentist	95	23.6		
Outpatient care/secondary care/specialist	44	10.9		
I had one-day surgery (without hospitalisation)	11	2.7		
In-patient care/I was hospitalised without surgery	12	3.0		
I had surgery and was hospitalised for a while	7	1.7		
Emergency care with serious/life-threatening conditions	15	3.7		
I participated in a preventive medical screening	25	6.2		
Do not wish to say	99	24.6		
Have you felt the need for mental health counselling in Hungary				
Yes	189	44.2	123	<0.001
No	204	47.7		
Do not wish to declare	35	8.2		
Did you have access to such services				
Yes	81	19.3	57.9	<0.001
No	131	31.2		
Partly	62	14.8		
Not applicable	104	24.8		
Do not wish to say	42	10		
Did you get the necessary mental healthcare				
Yes	44	10.6	163	<0.001
No	138	33.2		
Partly	34	8.2		
Not applicable	155	37.3		
Do not wish to say	45	10.8		
Did you have any difficulties in accessing healthcare (in general) in Hungary				
Yes	176	41.7	97.6	<0.001
No	200	47.4		
Do not wish to declare	46	10.9		

α = statistically significant.

When they were asked about getting any information on their entitlements to health services in Hungary, 35.9, 32.9 and 31.3%, respectively, responded yes, no, and partly. These proportions were not statistically different (*p* > 0.05). For those respondents who had information, the Tempus Public Foundation/ Stipendium Hungaricum website and the university administration website were the leading online websites that provided respondents with such information. While 45.7% resorted to the Tempus Public Foundation/ Stipendium Hungaricum website for their information, 41.8% depended on the University administration website. These were closely followed by those respondents who relied on their friends and family for information (39.9%), and onsite at the university administration office (22.1%). A little over half (50.6%) of the respondents said they could have full access to free public health services in Hungary. A relatively lower proportion of 4.6% of the respondents also stated that they had to pay for all public health services, while 41% of the international students surveyed have not had any health problems that required medical attention. On the contrary, 54.1% of respondents also indicated that they have been faced with health problems that required attention since they arrived in Hungary. Of the 54.1, 27% always received the needed healthcare, 25.7% received healthcare in most cases, 11.1% in some cases, and 14.6% said they never received the healthcare services they required. Preliminary health check/aptitude test upon arrival at the university, primary care/GP services with acute problems, and visiting a dentist were the major medical care services respondents claimed had received since their arrival in Hungary, with respective proportions of 38.1, 32.8, and 23.6%. Exactly 3.7% indicated they have had life-threatening conditions and had to receive emergency care. This proportion, however, was significantly (*p* < 0.05) lower than the three topmost medical care services received by international students in Hungary.

Although 44.2% of respondents indicated they felt the need for mental health counselling, 47.7% also contended that they do not need any mental health counselling in Hungary. Of the 44.2% who felt the need for mental health counselling, 31.2% did not get access to such services, whereas 19.3% did get access. Consequently, only 10.6% of the respondents got the necessary mental health counselling, while 33.2% did not get it. In general, the proportion of international students in Hungary who are likely to face difficulties in accessing healthcare and those who are likely not to face any difficulties was not significant (*p* > 0.05). While 41.7% indicated they were likely to face difficulties in accessing healthcare in Hungary, 47.4% stated otherwise.

### Difficulties impeding healthcare access ratings

Respondents were asked to rate some difficulties that may impede healthcare access in Hungary. The result is summarised in Table 4. For language barriers and lack of qualified interpreters, respondents rated this difficulty as very likely to impede healthcare access in Hungary for international students, with a significant proportion of 39.7% agreeing to this. While cultural barriers and misunderstandings are somewhat (30.6%) likely to impede healthcare access, religious barriers are not likely to impede healthcare access, with more than half (52.8%) of the respondents attesting to this. Similarly, the majority of the respondents stated that the lack of information about entitlements and the lack of providers’ information about entitlements will neither impede nor improve (neutral) healthcare access, with respective proportions of 33.3 and 34.3%. While the majority (31.8%) agreed that the lack of translated informational materials is somewhat likely to impede healthcare access, the lack of vaccination documents will not at all impede healthcare access, with the significant (*p* < 0.05) majority, 40.2% agreeing to this.

**Table 4:** Difficulties impeding healthcare access ratings.

Characteristics	Levels	Proportions (%)					Chi-square (χ^2^)	*p*-value (α ≤ 0.05)
		Very much	Somewhat	Neutral	Not really	Not at all		
Estimate how much the following difficulties may impede healthcare access in Hungary	Language barriers, lack of qualified interpreters	39.7	32.4	17	5.9	5	206	<0.001**
	cultural barriers, misunderstandings	12.5	30.6	28.6	18.3	10	68.5	<0.001**
	religious barriers	2.6	4.6	22.2	17.9	52.8	319	<0.001**
	Lack of information on the structure of the health system	17.3	28.4	31.6	11.9	10.9	73.4	<0.001**
	Lack of information about my entitlements	14.8	25.1	33.3	13	13.8	63.4	<0.001**
	Lack of the provider’s information about my entitlements	12.1	28	34.3	12.3	13.4	85.8	<0.001**
	Lack of translated informational materials	29.6	31.8	23.6	7.2	7.7	113	<0.001**
	Lack of my vaccination documents	3.8	11.3	25.8	18.9	40.2	152	<0.001**
	Administrative problems with my health insurance	9.4	13.2	25.9	18.5	33	71.8	<0.001**
	Lack my translated (previous) documents	9	15.6	22.8	17.9	34.6	71.4	<0.001**
	My personal financial problems	9.9	12.9	25.3	15.7	36.2	91.3	<0.001**
	Previously prescribed medications are not available	5.2	8.8	27.7	16.3	42	174	<0.001**
	Non-availability of mental health services	8.3	11.7	27.3	18.2	34.5	91.1	<0.001**
	Lack of social services for immigrants	16.7	23.3	28.4	11.1	20.5	33.6	<0.001**
	Discrimination by healthcare providers	6.2	16.2	28.7	15.4	33.6	95.3	<0.001**
	Others	6.6	10.4	38.7	10.7	33.5	154	<0.001**

Statistically significant at **p-value<0.001 and *p-value<0.05.

The administrative problems with health insurance, the lack of translated (previous) documents, personal financial problems, the unavailability of previously prescribed medications, the non-availability of mental health services, and discrimination from healthcare providers were all not likely to impede healthcare access for international students in Hungary. The proportion of respondents who were like-minded to this rating was 33.0, 34.6, 36.2, 42.0, 34.5, and 33.6%, respectively, for administrative problems with health insurance, the lack of translated (previous) documents, personal financial problems, the unavailability of previously prescribed medications, the non-availability of mental health services, and discrimination from healthcare providers. On the other hand, the lack of social services for immigrants will have a neutral effect on healthcare access for international students in Hungary, with 28.7% being the significant majority.

### Socio-demographics and changes in health status after arrival in Hungary

Generally, men had their health status significantly or slightly improved than women upon arrival in Hungary, as presented in Supplementary Table 1. As a result, the health of women deteriorated more than compared with men upon arrival in Hungary. Those respondents in the age category of 21–25 years had their health status improved (significantly and slightly) than any other age group. When respondents’ regions based on UN SDG regions were considered, those coming from Central and Southern Asia were the significant (*p* < 0.05) majority who claimed their health status significantly improved upon arrival in Hungary. Those coming from Europe and North America, on the other hand, were the significant majority (*p* < 0.05) who said their health status either significantly or slightly deteriorated. The respondents’ regions based on UN SDG regions had significant (*p* < 0.05) associations with respondents’ health status rating upon arrival in Hungary. The religious background and educational level of respondents also showed significant (*p* < 0.05) associations with their health status rating. For religion, the significant majority who claimed an improvement (both significant or slight improvements) in their health status upon arrival in Hungary were Muslims. In contrast, non-believers were the major group whose health status deteriorated significantly or slightly upon arrival in Hungary. When their level of education was considered, those with a high school graduation certificate or equivalent had their significantly deteriorated health status compared to any other educational level. On the other hand, the significant value (*p* < 0.05) majority of respondents with a bachelor’s degree or equivalent had their health status significantly improved upon arrival in Hungary, than any other educational level.

## Discussion

The survey was conducted to gain insight into the state of health of international students in Hungary amid the COVID-19 pandemic. This cross-sectional study helped identify the challenges foreign students encountered and how they adapted to their new environment for academic gains. The findings of the study brought an exposition to valuable information surrounding how their healthcare needs were met. In this discussion, we elaborate on the issues raised and compare them with existing literature, making recommendations for greater benefits.

### Health status by socio-demographics

Gender differences of participants showed traits of resilience in withstanding migration fatigue in favour of males. As shown in Supplementary Table 1, female participants who suffered health breakdowns were more than their male counterparts. This finding reverberates with the work of Trapollini and Giudici (30), who lent credence to the observation that migrants grow weaker in health after staying in a foreign country for a long period of time. Aside from that, health status differences between migrants and non-migrants are marginally more distinct among women than they are among men (31). This led them to posit that there are factors responsible for the unique trajectories travelled by migrants depending on their gender. Another study, which corroborated our findings, asserts that the disadvantage suffered by women was due to insufficient attention to preventive and screening measures for morbid medical conditions such as metabolic diseases, obesity, and perinatal problems (32). Further confirmation solidified that apart from ailments common to both genders, such as infectious diseases, female migrants may encounter additional difficulties in accessing healthcare because they are mostly relegated to the background for socio-cultural reasons (33). Moreover, women, by their vulnerable status as migrants, become exposed to numerous health risks, of which physical abuse and sexual violence are but a few (34). Narrowing down to the health problems that were of concern to students, as shown in Table 2, 33 of them, representing 7.7%, pointed to sexual and reproductive health issues. Of this figure, 19 (4.4%) were men, and 14 (3.3%) were women. Reasons were not given for the concerns, as this was a closed-ended question. However, existing literature explains that male international students are more sexually active than their female counterparts, hence stand a higher risk of exposure to sexually transmitted infections (35). For obvious reasons, these behaviors are common and may possibly occur without the use of sheaths or condoms to protect them from the infectious body fluids of their sexual partners. This evidence inevitably accounts for the differences in gender susceptibility to poor health as documented in this study (36).

Female international students may be vulnerable to violations of their feminine rights in exercising their rights to negotiate and participate in safe sexual activities. More disturbing is the fear of being stigmatised after falling victim to indiscriminate sexual violence due to socio-cultural norms that frown on women being openly expressive in their sexual exploits (37). Again, they most likely will not patronise sexual and reproductive health services despite the availability of such services to them because they do not want to be part of the statistics. These observations prompted some researchers to advocate putting the life course into view as an important factor in understanding the aging process of migrants (38). Their finding was supported in the recommendations made in a separate study that suggested a conscious effort at nurturing health throughout the course of life, believing it will favour its operationalisation and optimise population health (39). If an underserved community of international students is to benefit from more strategic interventions, timely support programmes, and basic to the development of optimal health will be necessary. By paying particular attention to those emerging trajectories and processes that are most significant to prevalent adult patterns of illness and developing effective ways to address them, researchers can use life course interventions to propel health trajectories on their right track and further optimise them (40) through integrated multifaceted approaches (41). In doing so, the gaps created by inadequate supplies in the face of increased demands of international students, specifically those in Hungary, will reduce the inequalities recorded in this study (42).

Regarding participants’ region of origin, our findings showed that those from Central and Southern Asia experienced an improvement in health while in Hungary. Regrettably, the ones from Europe and North America reported deteriorated health after relocating to Hungary. Eastern Europe, of which Hungary belongs, is reported to have the highest odds ratio of poor health concerning nativity. The perception that long stay abroad impacts migrant’s health negatively was confirmed by Lanari and Bussini (43), and was evident in the number of semesters participants had spent in Hungary and its related consequences on their health. Some other reasons for this outcome could be a result of the variations in how UHC is perceived and implemented in these destinations. Almost every country in Western Europe is said to have universal health coverage, and so is Hungary (44). The disparities with the region of origin could be partly due to differences in the culture of the people (45).

In terms of education, respondents with high school certificates also experienced poor health relative to respondents at other educational levels. These findings were backed by a study that found graduate students usually exhibit greater levels of resilience than undergraduates. In the context of religion, the health of Muslims improved while that of non-believers deteriorated. Religious beliefs may not be empirically proven. Nevertheless, they have an impact on those who practice them. According to Karl Marx, religion is “a fantasy that allowed people to blame their degraded lives.” In an elaborate expression, Marx stated, “Religion is the sigh of the oppressed creature, the heart of a heartless world, and the soul of soulless conditions. It is the opium of the people” (46). Strangely, religion rather moulds the beliefs and behaviors of practitioners and has been confirmed that religiously practising its doctrines improves the impact of health interventions (47, 48). Non-believers, on the other hand, hang on to no supreme force as backup for encouragement, hence their inability to resist stress, hence the deterioration of their health as reported in this study (49). The Islamic faith, to a large extent, incorporates concepts of health beliefs, health tips, and social care into practice (50, 51) and even promotes mental health after traumatic experiences, as observed in the outcome (52).

The study also revealed there was a significant relationship between differences in gender, origin, educational and religious backgrounds, and participants’ health status after migrating to Hungary. This could be a result of their physical and psychological make-up manifesting in their adaptability in their new environment. In the context of existing literature, sociologists have explained that associations between demographic characteristics, sociocultural adjustment, and psychological wellbeing accounted for changes in different sociocultural spheres of growth along the life course and were the most precarious for the psychological wellbeing of immigrants. In the absence of apparent factors such as discrimination, predictions of depression can be made from participants’ responses. Consolidating our findings is the conclusion drawn by Counted (53) that sociodemographic factors remain important variables for discussing the sense of place after they investigated the sociodemographic associated with attitudes toward specific geographic settings of migrants in general.

### Medical services patronised since arriving in Hungary

Considering the kind of medical services sought by students as captured in Table 3, visits to the GP were mainly because of infections (132, 32.8%) and chronic illnesses (60, 14.9%). Juxtaposing these proportions to the total number of participants, approximately 50% of international students had health issues after settling in Hungary, an outcome synonymous with existing literature (54). Proponents of migration health have long advocated interventions for persons in motion over long distances with limited access to healthcare services. They further buttressed their stance not only in line with philosophies underpinning anthropological reasoning but also with empirical evidence of changes occurring in the human body during migration. Unfortunately, the gap between health promotion and disease prevention activities remains wide among vulnerable populations such as foreign students (55). Reasons accounting for the high numbers of infections can be attributed to the COVID-19 pandemic. Suffice it to say that the baseline data of international students were established at the time they arrived in Hungary. The adequacy of how much personal space each student had to themselves in their various dormitories or apartments of residence, lecture rooms, or theatres may have also provided the vehicle that worsened the already volatile, contagious situation. Conditions might have also been compounded by the unexpected influx of a marauding crowd spilling over from the Russian–Ukrainian war when Hungary, in that trail-blazing humanitarian gesture, gave room to the masses fleeing an acquisitive pandemonium (56). Records even confirm that Ukraine has lower SARS-CoV-2 vaccination rates than Hungary, hence a greater possibility that some spread must have resulted from that emergency infiltration (57).

Migrant students in Hungary living with chronic diseases are often disadvantaged, as chronic illnesses require a lifetime of medical treatment management for optimal health. Therefore, delays in accessing timely care are a way of silently courting complications; thus, the fear of the unknown may pose a mental problem to some participants in the study (58). The situation as it stands paints a gloomy picture of the health needs of international students. This finding complements the observation of Mucci et al. (59), who, after their systematic review on the psychological health of migrant workers, uncovered that disorders emerging chiefly from the research were low concentration at work, depressive moods, and anxiety. These were conditions arising from marginalisation, amongst others, for which commitment to occupational medicine would be a wholesome remedy in vanquishing those occupational triggers.

### Making space for diversity and integration

As shown in Table 4, respondents’ estimation of cultural barriers and misunderstandings becoming an impediment to healthcare access in Hungary was tipped for “very much” at 12.5% and somewhat at 30.6%, altogether 43.1%. This figure is greater than the stakes for “neutral”, “not really”, and “not at all” (17, 5.9 and 5% respectively). The expression of international students’ opinion regarding this outcome simply communicates their desire to see and benefit from a multi-culturally oriented health service. Hitherto, they had indicated by popular affirmation that they trust care professionals in their home country more than they do for caregivers in Hungary; a clue for Hungary to run with in allaying the fears of foreign students by birthing its success of an enviable multiethnic health hub within the EU that will draw not only the patronage of international students but the broader community of Europeans within its sub region. But the situation confronting international students in Hungary may not be an isolated case. There is diversity and a lack of integration regarding healthcare provision across Europe due to policy differences between healthcare systems and social services, leading to poorly coordinated outcomes (60). Migrants in general are susceptible to chronic physical health conditions and mental health (61). For this reason, the WHO charges all countries to build robust and resilient healthcare system of sound quality that responds to the needs of all members in their population, including those who are vulnerable, e.g., refugees and migrants (62). Healthcare that is comprehensively and equitably reachable must be provided for these student migrants in national contexts and respond to intricate and emerging individual needs (63). The commitment towards actualising this goal is the heartbeat of the World Health Organisation in the European region, even to the point of demanding the inclusion of undocumented migrants in an integrated healthcare plan for the entire population (64). Lack of funds and of a trained and stable workforce, organisational shortfalls, and poor synchronisation of activities across the different horizons of management hinder the provision of healthcare for migrants (31). Reflecting on the high numbers of international students in dire need of healthcare amid such rich and caring continental provision, more will have to be done for the population of foreign students by the Hungarian institutions as a matter of duty and global prospect.

### Difficulty in accessing healthcare and the discipline of equity

The findings in Table 3 echo a void in the target of having every member within the international student community satisfied with the freedom to access healthcare at their own will when deemed right by them. While some got it always and others had it in most cases, there were those who never had a chance. Factually worrying is the realisation that a staggering 14.6% of these students never got the services they required, an outright contradiction to the WHO’s fight of leaving no one behind as far as universal health coverage is concerned (5). By design, several factors have been implicated as common hitches that buffet international students. Our finding is supported by a study conducted in Hungary to compare the prevalence of acute infection and seropositivity of SARS-CoV-2 among healthcare workers (HCWs) and medical students. A total of 1832 students recruited in a study (53%) were international students, while 682 (20%), Hungarian students had a lower prevalence of seropositivity. High prevalence was recorded among international students (65). By the proportions, an assumption can be made that more international students would need a doctor’s attention than domestic students would. In effect, seeking medical attention is the fulcrum around which recovery evolves (66). Unfortunately, it appeared that affected students encountered some obstacles in reaching out for these services. A simple prediction of the underlying cause may be alluding to bureaucratic and management issues rather than geographical or infrastructural causes (67). Deprivation suffered by the overwhelming numbers may have unleashed a greater burden of the contagious respiratory infection on the population. Even so, though bureaucratic demands were necessary, in some jurisdictions, a call was made to suspend bureaucracy in the provision of essential services during the pandemic because of its time-consuming demands as part of global efforts to nip the spread of infection in the bud (68). Possibilities of racial and ethnic discrimination cannot be ruled out in interrogating the reasons why those concerned were not given the chance, if the results are anything to go by. Authors have remarked that although instilling moral values in students is appropriate, strategic investment for the futuristic goals of a healthy population is necessary to sustain multiracial student communities (69). The need for primary prevention in mental health should be examined closely by policymakers worldwide (70), with a focus on both individuals and groups, since peculiarities in challenges may exist, as an international student may not be directly affected but may be a member of a group severely underserved (71). Formidable in confirming this outcome is the unbiased, unpolarised, and dispassionate follow-up exploratory study of Atiku and Adofo (72) that unearthed the difficulties international students encountered in their various attempts at accessing healthcare services in Hungary. Their findings were also corroborated to a large extent by Lannert and Derenyi, who recorded similar findings for which they recommended a shift of focus from mobility to quality (73).

### Limited insurance coverage

Health insurance solves the problem by half, covering the cost of consultation, examination, and diagnostic procedures as indicated in the Stipendium Hungaricum scholarship package for all beneficiaries. The absence of insured health simply means inaccessible healthcare (74). According to the Hungarian Central Statistical Office, 11.4% of the entire student population in institutions of higher education are international students. Out of this, 4.4% are Stipendium Hungaricum scholarship holders. This implies that a significant 7% of foreign students may either be beneficiaries of other forms of scholarship or self-funding. Secondly, self-funding students are, by regulation, under an obligation to ensure their health during the admission process. Deductively, some form of insurance coverage must have been in place for every single student. Therefore, the ‘obstructed’ access to services for international students depicted in Table 3 could be related to reasons other than health insurance coverage (75). As a minority group in a foreign country, coverage of the population concerned and the kind of services accessible to members are usually limited, as revealed in current scientific literature (76). It has also been on record that without health insurance, it is usually difficult and almost impossible for students to bear the full cost of cash and carry, the reason for which institutions arrange for affordable insurance services for their students. It is a global phenomenon that visiting a specialist and receiving some treatment could cost so many dollars in the United States, while a hospital stay of only three days could cost about 10,000 dollars. Interestingly, the average cost of healthcare in Hungary for international students is between 200–300 EUR per year. Relatively, Hungary offers an affordable alternative; nonetheless, it could be challenging for a lot of foreign students. Weighing the options available, consequences for denied access are ominous and devastating since essential services like vaccinations and management of critical conditions risk being missed and complicated (77). The TAJ card, however, covers basic ophthalmic and dentistry services such as consultation and examination fees, but not prescriptions for lenses or aesthetic procedures, thereby precipitating a limiting factor to accessing complex services (78).

### Communicating on chaotic wavelengths

The stakes were high (39.7%) on the Likert scale in estimating that language barrier and the lack of qualified interpreters are impediments to accessing healthcare in Hungary. Study participants indicated language was their most significant barrier to accessing care through the ratings of the possible options among 14 other variables in its category. It has been noted in other studies that suggest international students were not able to effectively communicate socially and academically, a situation that retarded their ability to acquire new conversational skills in their host country. Indeed, the same observation has been made in this study. This assertion was corroborated by Ibragimova and Tarasova (79), who conducted a study on language-related problems of international students in a Russian university and concluded that international students in non-English speaking countries contend with the challenges of language difficulties. The Hungarian society is mostly condensed in its local language. A significant 98.9% speak Hungarian, English 25.3%, German 12.6%, Russian 2.1%, French 1.5%, Romanian 1.4%, and other 5.1% (80). Unfortunately, the process of patient-caregiver communication is hampered by a strong language barrier. This deficit negatively impacts the quality of care that is rendered or received. There are potential risks to students’ health if their health context, language, and beliefs differ significantly from the understanding of health in the Hungarian context. Misunderstandings can occur, and delays in diagnoses pose a threat to their health and safety unless assistance is sought from people who speak both languages. Until then, the integrity of privacy and confidentiality may be seriously compromised (81). In summary, the importance of this challenge was admitted in the honest confirmation made by Lannert and Derenyi: “Half of the respondents think that insufficient foreign language competence of students, lecturers and the administrative staff is the most important obstacle to internationalization in higher education. The second most frequent mention was shortage of funds. About one in four respondents mentioned structural problems and problems with human resources” (73). Resorting to medical interpreter services for foreign students in Hungary may be a means, agreeably (82). However, to correct this cacophony, hospitals in Hungary should be making use of language translator applications that suit this digital dispensation. Introducing a mandatory language training for international students in the first 6 months of their studies, as it is with countries like Germany, is likely to improve the current situation (83). The findings of this study bear consequences for both the international students’ community and their host country, Hungary. The first point of intervention should be a step towards standardising care and introducing some flexibility in the bureaucratic processes that Lannert and Derenyi (73), described as rigidly unproductive. Improving outcomes should be achieved by phasing out old-fashioned practices that sink the efforts of well-meaning caregivers and pushing for regular research-informed interventions to redefine healthcare in a modernised and acceptable fashion. Secondly, the distinction between perceptions and perspectives will require a concentrated and coordinated commitment from an interdisciplinary health industry team (84).

## Limitations

Appreciating the commitment involved in this study, we are hopeful the findings will positively impact the interests of international students in multiple areas, especially in the advancement and enlightenment of their entitlements to the best patient-centred healthcare. That notwithstanding, we acknowledge that our findings may not necessarily represent the exact situation in all other institutions of higher learning in Hungary. Our study also featured only international students with no concession to health caregivers. We also acknowledge the limitation of not collecting data on the exact immigration periods of our respondents, realising that this could have offered us a chance to disaggregate data and zoom in on some details. Finally, the unequal representation of religious groups for a balanced quorum on issues of religiosity was not a deliberate act but rather the exact reflection of the study population. A major limitation encountered in the study was the apathy that set in from the part of students who had indicated in their response to our survey questionnaire a desire to participate in a follow-up focus group discussion to offer a deeper insight into their experiences and views on health, healthcare and wellbeing. Unfortunately, time and available resources also constrained us from extending the study to include native students and hospital caregivers albeit the contributions of 440 international students are representative enough to be the small weight needed to balance the scale of equity in dispensing good quality healthcare to minority populations, of which international students are a category.

## Conclusion

This study highlights major difficulties encountered by international students in Hungary while navigating the intricate healthcare system of Hungary. From the findings of the study, international students continue to battle with access to healthcare in Hungary as it is in other parts of the world. While geographical location may remain the primary factor for this impediment, the lack of access due to inharmonious linguistic deficiencies between students and caregivers creates a state of confusion, further sinking the attempts of many foreign students in need of medical attention. To continue in this trajectory will only thwart global efforts at improving health outcomes for all and sundry. The impression created by this data portrays access to health services as an atrium-type space in the middle of a wide corridor, with many yet to know which direction to go for caregiver attention. This research is expected to help in reviewing policies and developing significant community partnerships that foster evidence-based interventions. More importantly, it should be embraced by limitless horizons in the strength of neuro-divergent harmony (moving beyond tolerance and toward acceptance and understanding) without acrimony or animosity for an affirmative action that nurtures a home-away-from-home kind of environment for international students in Hungary.

### Recommendations for future studies and use of data

Through the identification of credible associations among variables and the formulation of specific, testable hypotheses that can be evaluated in a structured or comparative design, novel research studies can be engineered from the findings of this research. Included are the following:

1. Descriptive studies, which will add meaning to the numbers depicting the prevalence of barriers to access in the exploratory phase, with a follow-up comparative study that examines how access differs across demographic groups or geographic regions. In essence, public health specialists can conduct intervention studies that test strategies to improve access, based on the empirical acumen benefited from this current study should be greatly considered in:
  - Cross-sectional studies to compare different groups at a single point in time.
  - Longitudinal studies to track the same groups over time to assess changes in access.
  - Case control studies can be carried out for explanatory purposes.
2. Intervention studies may be used to test the effectiveness of interventions designed for improved access based on the findings of the exploratory study.
3. The use of a mixed method approach to confirm findings.
4. Resourcing data sources with new findings as standard evidence for reference and cutting-edge studies.

## Data Availability

The original contributions presented in the study are included in the article/Supplementary material, further inquiries can be directed to the corresponding author.
