# Supplementary material for: Knocking for gold. How long must I? A survey report on international students seeking healthcare in Hungary
Source: Front Public Health. 2026 Jan 22;13:1624806. doi: 10.3389/fpubh.2025.1624806 (PMC12872748; doi:10.3389/fpubh.2025.1624806)
Supplement: Supplementary file 2 [file Data_Sheet_2.PDF]

Manuscript #1624806
Editorial comments addressed: Reviewer 1

1. Under the introduction section, the authors write, “This has contributed to making Hungary an attractive destination for international students.”. Is it possible to view in lens of **the impact of Covid-19 pandemic on the in-migration of international students in Hungary as well**? What were the **additional health conditions and associated vulnerabilities** that international students had to confront relative to the native students due to pandemic and post pandemic situation?

Impact of Covid-19

This has contributed to making Hungary an attractive destination for international students **with dominance in the field of medicine due to the introduction of English-language programs, tailored specifically for international medical students**. The outbreak of SARS COVID-19, despite its contagious nature did not dim the chances of both native universities and prospective students. Rather, measures were put in place to minimize the chances of cross border spread through eligibility criteria defined by state authorities [6]**.** Impacts of the pandemic rippled in many unfathomed dimensions. Students arriving had to complete forms at the border detailing whether they carried signs and symptoms of the respiratory infection. Questions regarding the PCR (Polymerase chain reaction) test within a time frame of 48 hours were also confirmed at the immigration check point before allowed entry into the country [7]. Marked changes occurred including an unprecedented drop in the number of international students in Hungary. It was reported that a significant 15% dip was recorded from the previous year resulting in the enrolment of only 35,000 in 2020. The disruption was also felt in the call for vaccination at approved hospital centres with restrictions on travel activities [8]. The pandemic also narrowed international students in their movement and meetings as lectures became an online engagement compounding their challenges to even socialize in activities of fitness or leisure that nurtures friendships in cultural diversity. While some fresh students were battling with acculturation issues including nutrition and dietetics, [9], others had their graduation in limbo [10]. Evacuation of native students from hostels to create the recommended distance allowance for foreign students became crucial. Amidst these difficulties was the yoke of unemployment hanging on the neck of international students both for personal survival and remittance to families back home. The transition phase reported outbursts of racism and xenophobic sentiments which overwhelmed the mental health of students. Students of certain nationalities were said to have been tagged as carriers of the viral infection [11]. Feelings of isolation, loneliness, and homesickness were widespread among students opening the

floodgates for mental health problems to creep in. Complaints of anxiety, depression were dominating with many while some struggled with post-traumatic stress disorder (PTSD). Ill health was rampant predominantly from the quarters of undergraduate students [12]

2. Under introduction section, "Some prescription drugs are generally subsidised, which substantially reduces costs for patients (April International)."…**What is April International**? If it is a citation, please follow the journal guidelines for referencing.

*April International is an insurance provider. The citation is as follows:

April International, "Hungary Health Insurance 2025 - For Foreigners + Expats," ONLINE, Accessed April, 21, 2024.

3. Introduction section, "After a survey conducted between 2021 and 2022 to establish…..to GP services when they needed them (**University of Pecs Information about GP**)." If 'University of Pecs Information about GP 'is a citation, please follow the journal guidelines for referencing. Can explain about the sources in parenthesis.

University of Pecs International Centre- PTE, "Medical Care," [Online]. Available: https://international.pte.hu/current-students/information-about-services/medical-care. [Accessed 17 July 2025].

4. The manuscript would improve its readability using copyediting services. The **full forms of abbreviations** must be mentioned in the first instance of usage. This is missing…

Corrections have been made in the write-up.

5. Under the Research setting and tool development section, authors mention 'active and passive students in semester'. Please explain the terminologies by definition in the section. Is there any time threshold for students to be regarded as active or passive?

Explanation to the terminologies-active and passive student:

An active student is the student who has registered for a semester and is actively participating in their studies, including attending lectures and fulfilling academic requirements. A passive student, on the other hand is that one who has temporarily suspended their studies for a semester. Such students do not take classes neither do they fulfil any academic requirements. Students can declare their semester status as either active or passive at the beginning of each semester in the Neptun system (University Portal System).

6. In Table no. 1- please mention 'years' beside the age groups as unit.

Same effected:

Table 1: Showing the Sociodemographic Characteristics of the Study Participants

Variables	Levels	Frequency	Proportion (%)	Chi-square (χ^2)	p-value ($\alpha \leq 0.05$)
Gender	Female	235	53.4	2.05	0.153
	male	205	46.6		
Age groups	Below 20 years	80	18.2	176	<0.001
	21-25 years	187	42.5		
	26-30 years	98	22.3		
	31-35 years	52	11.8		
	Above 35 years	23	5.2		

7. In the Likert Scale use, a 3-point, or 5-point scale would have been better to capture nuances in the response. What is the timeframe referred to when asking the students ‘How would you rate your health (Recently)’. What does the term ‘recent’ refer to as regards to the timeline from arrival?

The timeframe applied to the word "recent" in our question on how students would rate their health typically referred to the past few weeks to months within the span of the semester at the time data were collected.

8. Table 2 showing the self-assessment of health status could be presented in gender disaggregated form to identify the gender difference in health status. For e.g. it would provide better understanding whether the SRH issues and mental health conditions are disproportionately impacting health outcomes across gender.

Narrowing down into the health problems that were of concern to students in Table 2, 33 of them representing 7.7% indicated they were worried about sexual and reproductive health issues. Of this figure, 19 (4.4%) were males and 14 (3.3%) were females. Reasons were not given for the concerns as this was a close-ended question. However, existing literature explains that male international students are more sexually active than their female counterparts hence stand a higher risk of exposure to sexually transmitted infections(STIs), Human Immunodeficiency virus (HIV) and Acquired Immunodeficiency Syndrome (AIDS) [37]. This revelation could be lending clues to their vulnerability to indulge in risky sexual behaviours under the influence of alcohol and cigarette smoking. For obvious reasons, these behaviours are common and may possibly occur without the use of sheaths or condoms to protect them from the body fluids of their sexual partners [38] [39].

On the flip side, female international students may be vulnerable to violations of their feminine rights as in exercising their rights to negotiate and participate in safe sexual activities. The fear of associated risks to contraction of sexually transmitted infections and unwanted pregnancies could be triggers to disruption in their mental processes [40]. More disturbing is the fear of being stigmatized after falling victim to indiscriminate sexual violence due to socio-cultural norms that frown on females being openly expressive in their sexual exploits [41]. International students are reported to be aware of protective measures in avoiding all forms of sexual mishaps. Strangely, these mechanisms are underutilized for sake of culture. While sexual and reproductive units of student clinics may be at their disposal, they may not patronize those facilities because they do not want to be part of the statistics [42]. A number of studies have supported this fact and these are some of the reasons mental health services should be better resourced to provide unlimited guidance and counselling services to all manner of students [43] [44].

9. What was the rationale for asking a separate question on dental health. As mentioned in the Table no.2. 'Do you have any longstanding dental problems?

The rationale behind asking the question "Do you have any longstanding dental problems?" was to follow the nomenclature under which the two practices have been placed. Dentistry is separated from general medicine primarily due to its distinct focus on oral health. Dentistry requires specialized knowledge and skills, and historical factors shaping professional autonomy and practice. While there's growing recognition of the link between oral and overall health, the two professions have evolved in separate educational pathways with separate licensing agencies or bodies, and professional organizations. This sovereignty of each has been reinforced through legislation, education, and service delivery (Simon**,** 2016**).**

Lisa Simon **(2016). Overcoming Historical Separation between Oral and General Health Care: Interprofessional Collaboration for Promoting Health Equity**, *DMDAMA J Ethics*. 2016;18(9):941-949. doi: 10.1001/journalofethics.2016.18.9.pfor1-1609.

10. There must be representation of disaggregated data on period of immigration of the students in Hungary in Table 1, using when the data was collected as a time of reference. This would provide a better comprehension of health status changes over time. Recent migration, less than 6 months, less than a year, less than 2 years and so on…

Thank you! Please, we appreciate your suggestion. Unfortunately, we did not collect data on the exact time respondents arrived in Hungary. However, the depth of your visualization has led us to consider it as a limitation

11. The title of the manuscript can be changed into which provides a clear picture of the content of the manuscript. The title reads a fancy one but fails to convey the research objective, location and study participants. 'Immigration, health status, Hungary, student population' etc can be used to form the manuscript title.

The title of the manuscript has been modified to:

Knocking for gold- how long must I? **A survey report on international students seeking healthcare in Hungary.**

12. In the discussion section- "As shown in Table 7, female participants who suffer…". There is no table 7 present in the manuscript or the supplementary material. Please recheck the inclusion and proper numbering of the tables. **?**

The reference now reads…As Shown in Table 5

13. Many sentences in the manuscript need to be rewritten to convey the meanings substantially. Some sentences are incomplete in the discussion section. For instance, "The perception that long stay abroad impacts migrant's health negatively was confirmed by [24]."…"Consolidating our finding is the conclusion drawn by [31]". It is appropriate for readability to mention names of the authors and then give citation in parenthesis. Or write 'authors' and then give citation. Throughout the manuscript the authors have used quotations in italics, but some places the citation is missing. Please add appropriate citation. For instance, "He further revealed "the situation in Eastern Europe and the countries of the former Soviet Union is highly variable." Who revealed ? What is the context and citation is missing.

Thank you! Modifications have been made to improve upon readability.

Almost every country in Western Europe is said to have UHC [46]. Romaniuk and Szromek (2016), revealed there are nuances in how health reforms have transitioned over the years toward UHC in Central and Eastern Europe bringing the countries of the former Soviet Union in perspectives with focus on the near similarities and subtle differences in their health systems. With this evidence, the idea of UHC in North America will be different from how it is interpreted in Hungary and this may affect the health of students from these origins who have come to study in Hungary. Therefore, this problem of deciding whether a given country has, or does not have universal health coverage is perpetuated by the lack of precision in defining its scope. In effect, disparities within regions of origin could be partly due to differences in the culture of the people [47].

14. The discussion section is required to be tighter in its arguments and references particularly when discussing religion and faith as a socio-demographic factor of influence on health status and uptake of healthcare services. Okay!

Several attempts have been made to strengthen the discussion:

Non-believers, on the other hand hang on no supreme force as backup for encouragement hence their inability to resist stress hence the deterioration of their health as reported by these authors [50]. For ages, people have relied on religion for hope and worthiness in times of ill health, trusting that there is a profound sense and purpose to overcoming physical and mental difficulties. In the circles of professional nursing, the spirituality of man has been greatly considered as an element to be held in full regard if their healing process is valued. It is a significant piece of holistic patient care towards recuperation. In fact, it has been emphasized in palliative care that when people are faced with fear of the unknown or are at a point of imminent death, many resort to religion for reassurance [52]. It is understandable therefore that the practice of religion has influence over how people reacted to the pandemic and escaped its harsh effects. Research continues to reverberate that persons who exercised faith in their religion habitually demonstrated greater resilience to recover from their illnesses and make new adjustments to cope with life. [53]. That irrespective, the debate over religion and improved social systems still take the centre stage in national development. For instance, there are societies that give excuses against medical treatments and public health interventions while others embrace the idea with zeal. A **classic example is observed in the survey that was conducted in Poland among two groups of residents** with data gathered during two distinct windows of the pandemic notably (1) **the final period of the third wave of the COVID-19 pandemic labelled "pandemic group,"** and (2) **the post-pandemic period** labelled **"post-pandemic group." Results showed no significant differences in accessing healthcare**

among the two categories. They concluded systemic factors were better involved [54]**.**

It has thus been proposed that while acknowledging religion, the role of religion in modelling attitudinal change toward healthcare, the essentials of an all-inclusive healthcare service is driven by a multiplicity of factors and not merely the existence of a pious distinctiveness [55]**.** Consolidating our finding is the conclusion drawn by Counted (2018), [57] in his work on religion and place attachment throwing light on the role of religious and spiritual experiences in people's understanding of their environment. He argued that sociodemographic factors remain important variables for discussing the sense of place theory, a disposition that was upheld by the investigations of Taylor, Everett and Edgar (2021), that ethnic factors are associated with attitudes displayed towards specific geographic settings of migrants hence not a one-size-fit all situation [58]**.**

15. Please mention the limitations of the study in a separate subsection.

Thank you! The section has been fixed as follows:

Limitations of the study

Appreciating the commitment involved in this study, we are hopeful the findings will positively impact the interests of international students in multiple areas especially in the advancement and enlightenment of their entitlements to the best patient-centred healthcare. That notwithstanding, we acknowledge that our findings may not necessarily represent the exact situation in all other institutions of higher learning in Hungary. Our study also featured only international students with no concession to health caregivers. We do also acknowledge the limitation of not collecting data on the exact immigration periods of our respondents, realising that could have offered us a chance to disaggregate and zoom in on some details. Finally, the unequal representation of religious groups for a balanced ‘quorum’ on issues of religiosity was not a deliberate act but rather the exact reflection of the study population that participated in the survey.

We are grateful for your comments!
