# Supplementary material for: Knocking for gold. How long must I? A survey report on international students seeking healthcare in Hungary
Source: Front Public Health. 2026 Jan 22;13:1624806. doi: 10.3389/fpubh.2025.1624806 (PMC12872748; doi:10.3389/fpubh.2025.1624806)
Supplement: Supplementary file 3 [file Data_Sheet_3.PDF]

Title of manuscript: Knocking for Gold- How Long must I? **A survey report on international students seeking healthcare in Hungary**

Authors:

1. Livia Yawa Like Atiku- PhD candidate (First author)
   University of Pecs Medical School
   Doctoral School of Clinical Medical Sciences
   Department of Public Health Medicine

2. Erika Maria Marek, PhD, Associate Professor (Coauthor)
   University of Pecs Medical School
   Department of Public Health Medicine

Abstract

Migration significantly impacts the health of international students in host countries. It has been reported in several studies that one of the main challenges of international students is gaining access to healthcare services. This study examines the experiences of international students in Hungarian universities, focusing on their self-reported health status, access to healthcare services, adequacy of services rendered, cultural sensitivity, and quality. This survey gathered responses from 436 students across major universities. Using a structured online survey and CART analysis, we examined socio-demographic influences on healthcare access and the availability of culturally competent care. Key findings reveal notable health challenges, with 28.3% of students reporting health deterioration since migrating to Hungary. Approximately 63% arrived with limited or no prior information on the healthcare system, and only 35.9% had a full understanding of their entitlements. Despite the Stipendium Hungaricum scholarship's insurance coverage, 30.9% of students incurred some out-of-pocket expenses, and 4.6% paid entirely for public healthcare. Trust issues also surfaced, with 36.7% placing more confidence in home-country providers compared to 20.6% for Hungarian providers. Issues of discrimination were implied from the data but not directly supported. These findings underscore critical policy reviews, including enhanced intercultural competence, better language support, and expanded mental health services.

Key words: health, access, healthcare, wellbeing, international students, Hungary.

Introduction

In a joint report of the World Health Organization (WHO) and the World Bank on global monitoring in the light of Universal Health Coverage (UHC), it was revealed that progress towards providing people everywhere with quality, affordable, and accessible healthcare has suffered a terrifying stagnation [1]. Captured in a precise and succinct statement of the Director-General of WHO was the concern about the glaring proofs that some people may be behind the bridge to universal health coverage if immediate steps are not taken to block it: *"The fact that so many people cannot benefit from affordable, quality, essential health services not only puts their own health at risk, it also puts the stability of communities, societies and economies at risk, We urgently need stronger political will, more aggressive investments in health, and a decisive shift to transform health systems based on primary health care."*

In European countries like Bulgaria and Germany, beneficiaries' claim to healthcare was primarily restricted to medical or surgical exigency, but in recent times has been upgraded to comprehensive coverage with the rollout of new regulations. Migrant health has been a central issue for many corporate bodies of global interest [2]. The World Health Organisation creates the awareness that *migrant-inclusive health systems improve public and global health outcomes for all*; preserving lives, reducing disease burdens and death rates [3]. With this mind, efforts were marshalled towards ensuring displaced persons from Ukraine acquired a new status that placed them at par with documented asylum seekers who have been integrated into the social security system of Germany [4]. This wave of humane assistance was replicated by Croatia, Hungary and Sweden granting entitlements in specific smaller groups of beneficiaries to the full state benefits coffers including (unaccompanied) children and adolescents below the age of 18 and (pregnant) women. Hungary, typically offered general health assessments to identify those who were in need of immediate health care of which some were compulsorily treated on arrival [4].

The Stipendium Hungaricum scholarship programme paved the way for over 11,000 international students to benefit from free tuition, comprehensive health insurance, regular stipend to cushion the cost of living and accommodation allowance for all levels of higher learning. [5]. This has contributed to making Hungary an attractive destination for international students **with dominance in the field of medicine due to the introduction of English-language programs, tailored specifically for international medical students**. The outbreak of SARS-Cov-2, despite its contagious nature did not dim the chances of both native universities and prospective students. Rather, measures were put in place to minimize the risks

of cross border spread through eligibility criteria defined by state protocols [6]. Impacts of the pandemic rippled in many unfathomed dimensions. Students arriving had to complete forms at the border detailing whether they carried signs and symptoms of the respiratory infection. Questions regarding the PCR (Polymerase chain reaction) test within a time frame of 48 hours were also confirmed at the immigration check point before allowed entry into the country [7]. Marked changes occurred during the period under review including an unprecedented drop in the number of international students admitted to programs in Hungary. It was reported that a significant 15% dip was recorded from the previous year resulting in the enrolment of only 35,000 in 2020. The disruption was also felt in the call for vaccination at approved hospital centres with restrictions on travel activities [8]. The pandemic narrowed international students in their movement and meetings as lectures became an online engagement compounding their challenges of not socializing with others in activities of fitness or leisure that nurtures friendships in cultural diversity. While some fresh students were battling with acculturation issues including nutrition [9], others had their graduation in limbo [10]. Evacuation of native students from hostels to create the recommended distance allowance for foreign students became crucial. Amidst these difficulties was the yoke of unemployment hanging on the neck of international students both for personal survival and remittance to families back home. The transition phase recorded outbursts of racism and xenophobic sentiments which overwhelmed the mental health of students. Students of certain nationalities were said to have been tagged as carriers of the viral infection [11]

Feelings of isolation, loneliness, and homesickness were widespread among students opening the floodgates for mental health problems to creep in. Complaints of anxiety and depression were dominating with many while some struggled with post-traumatic stress disorder (PTSD). Ill health was rampant predominantly from the quarters of undergraduate students [12]

As a result of these huge numbers, proactive steps were aligned with the call from global consultations on migration to accelerate deliberations on the role of scientific research in supporting evidence-based health responses associated with migration [5].

Generally, the number of overseas students in Hungary rose from 15,000 to 32,000, and their proportion among all students increased to17 per cent from 6.9 per cent between the 2010/2011 and the 2020/2021 academic years. By the 2023/2024 academic year, their number increased to 37,500 [13]. The percentage of all international students in Hungary expanded to 11.4 while 4.4. of the same group of students were Stipendium Hungaricum scholarship holders. International students are driven to study in their host nations for many reasons. Notably, they are enthusiastic at having new experiences discerning and performing in their study discipline.

Furthermore, studying abroad increases career opportunities due to the attainment of improved expertise for prospective economic engagement in their native country or other nations [14]. Besides, more often than not, international students obtain an extensive and additional flexibility in education than they would in the natural settings of their home country. Finally, they get the grounds to build a robust autonomous foundation that nurtures and sustains a stronger, strategic network of relationships from a multicultural perspective.

The prevailing circumstances confronting migrants from the start of journeying to the time of settling in their new environment potentially exposes them to life-threatening dangers that predisposes them to physical and mental disorders [15]. Some of these situations include, but are not limited to unequal access to healthcare services, susceptibilities related to migrant status (asylum seekers, refugees, foreign workers or international students), discrimination and exploitation due to austere immigration checks and policies guiding employment and other social-economic factors [16]. More often than not, common anti-migrant views held by members of a society also take a toll on these vulnerable migrants. Thus, with their influence on social life, they become the determinants of the migrant's health. Fixing these conglomerate of factors catalyses migrant's acculturation and integration into the new environment for progressive development [17].

In Hungary, international students in need of healthcare services have to observe a sequence of guidelines to secure an appointment for medical consultation. Arrangements for all appointments and registration are by email. Foreign students can also request support from a help desk by calling a designated phone line for an appointment to a consultation with the GP. Regardless of the choices, booking an appointment is a must if any of the services available are to be utilised [18].

After a survey conducted between 2021 and 2022 to establish whether international students were able to maintain their health after arriving in Hungary or whether migration has affected their health, and even more so whether they are able to access healthcare when they needed it, the responses revealed a significant number of them had maintained a stable health status over the time. Some observed changes had occurred and while others indicated they could not get access to consulting General Practitioner (GP) services when they needed them [19].

The revelation called for a review of Ashcroft's principle of support crystalized in a simple statement, "Many issues in professional ethics concern failures to respect a person's autonomy, ranging from manipulative under-disclosure of pertinent information to non-recognition of a refusal of medical interventions.", we embarked on this study to discover more [20].

Health financing continues to grow amidst the advent of novel trends in technology and demography as crucial elements for policymaking in healthcare. Consumers have become increasingly aware not only of their rights as patients but have also gained an understanding in the concept of quality healthcare. European healthcare systems usually provide coverage of most healthcare costs for their entire populace with systems structured to control healthcare spending at the national level [21].

Unfortunately, many countries are confronted with how to strengthen their health systems by bettering the quality and accessibility of healthcare while streamlining the expenditure on logistics and supplies [22]. Hungary offers its populace a relatively affordable health insurance package as compared to many of its other member European countries [23]. Generally, scholarship students are covered by a comprehensive national health insurance plan that exempts such students from making out-of-pocket payment for services except for prescribed drugs, some dental and ophthalmic cases. The national health insurance package absorbs the cost of preventive screening tests, out-patient care in the shades of internal medicine, skin disorders, urology, obstetrics and gynaecology including laboratory tests and other sophisticated diagnostic procedures. Ambulatory services, surgeries and a 24-hour call centre for consultation remain available to all foreign students [24]. In addition, the state of Hungary finances public health services to lift economic burdens off patients. The highly subsidised cost is manifested in the waiver of consultation fees ordinarily paid before seeing the medical doctor. The cost of some prescription drugs are generally slashed by half the price or more, which substantially reduces costs for patients [25]. This privilege of a comprehensive insurance coverage is activated when beneficiaries are active in a semester. Private entities also provide insurance services with a concession for foreign students at affordable rates as alternatives to the national provision**.**

Significance of research in transforming society

Crafting a space in the field of research for an advancement in human health is a gallant step in leaving no one behind [26]. Magnifying the usual and common movement of people through a humanitarian lens showcased a necessary spot for global health engagement. Thus, supporters of global health led the sculpturing of a giant step in a global movement to soften the hard, crusty ground that has been neglected in a million ages to merit the ardent attention of world health advocates [27].

Migration health has provoked an interest in varied geographic distributions. Scientific

research and the segregation of migrant groups for the better understanding of their peculiar health needs has aided planning appropriate interventions thereby driving the agenda for efficient service delivery in different migrant populations [28]. Research scholars and other significant stakeholders have solicited quality research works on both the international and domestic fronts in recognition of migration and health. This action is meant to propel SDG-3 into realisation [29]. International students in Hungary have been captured in research studies alongside domestic students in respect of the statistics and economic gains made within definite time periods. Vincze and Bács (2020), hinted that though the presence of international students in Hungary has boosted the nation's economy, the numbers of native students keep declining and therefore sadly threatens the future of their representativeness in their own institutions of higher learning [30]. Others have discussed issues in line with the acculturation of foreign students in their new environment using a cross-sectional study. One such study blew the whistle of a deteriorating general and mental health state among international students due to poor adjustment. However, the authors believed that by melting into the Hungarian culture the tension would ease [31].

Methods

Study design

Data were pooled and extracted from an online cross-sectional survey as part of an elaborate original study. The authors conducted the study to identify issues affecting the health and general well-being of international students in Hungary using a standardised questionnaire.

Research setting and tool development

A purposeful empirical survey was carried out in Hungary between May and August, 2021. An anonymous online survey questionnaire was served the international students' community by email from the goodwill office of The Tempus Public Foundation, the awarding agency of the Stipendium Hungaricum scholarship and also by the educational offices at the faculties of the University of Pecs portal, Neptun.

Eligibility for the completion of this questionnaire was to be a student in any of the public institutions of higher learning. This student must also have an active student status in the semester that the researchers collected data. Students who were passive for the particular semester were excluded from participation. **An active student is the student who has registered for a semester and is actively participating in their studies, including attending lectures and fulfilling academic requirements. A passive student, on the other hand is that one who has temporarily suspended their studies for a semester. Such students do not take classes neither do they fulfil any academic requirements. Students can declare their semester status as either active or passive at the beginning of each semester in the Neptun system (University Portal System).**

A total of 476 responses were received at the end of data collection but 436 were finally analysed after data purification. The difference of 40 were excluded from the analysis due to reasons such as+ uncompleted questionnaire and visible evidences of illogical responses.

Data collection method

Data were collected from a number of international students including Stipendium Hungaricum scholarship beneficiaries. An anonymous online survey questionnaire was sent to students in 4 Hungarian universities in no specific order or preference. There were sixty-three (63) questions in total for the questionnaire, two of which were open-ended and the rest, close-ended. There were questions pinned to the Likert scale to allow respondents to indicate their opinions, attitudes or feelings about their general and mental health since arriving in Hungary for academic purposes. The questions were in three main categories seeking information on their basic demographics, self-assessed health status, health habits and health seeking behaviours. Also included were questions on their expectations of healthcare in Hungary. Each close-ended question had options from which respondents selected from, depending on their reality or preference. There was also available among these, the option to withhold themselves from answering a particular question. Some areas that questions covered bothered on their self-assessed health status, health service availability, accessibility, affordability, acceptability and quality.

Data analysis

Descriptive statistics (e.g., response frequencies, percentages, and proportions) were used to summarize variables. Chi square goodness of fit and test for independence were employed to investigate the significant differences among studied parameters and socio-demographic variables. CART algorithms were employed to assess the relationship between some responses and socio-demographic variables of participants. Misclassification cost was used to select the most appropriate tree. Finally, p-values less than 0.05 (significance at 95% CI level) were considered statistically significant. Data were analyzed by SPSS software (windows version-26).

Ethical considerations

The study was conducted in accordance with the Helsinki Declaration and was approved by the Institutional Scientific Research Committee of the University of Pecs (nr. of approval: PTE/61924/2021) and the Hungarian Medical Research Council (nr. of ethical approval: BM/26490-1).

Results

Sociodemographic characteristics

The socio-demographic information of respondents is summarized in Table 1. The proportion of females (53.4%) in the study exceeded males (46.6%). Regardless, the difference was not significant ($p > 0.05$). The majority of respondents, comprising 42.5%, fell within the age group of 21-25 years, while only 5.2% were above 35 years old. These proportions indicate a statistically significant difference ($p < 0.05$) between the two age groups. The distribution of international students by United Nations Sustainable Development Goals (UN SDGs) regions demonstrated significant variations ($p < 0.05$). The highest proportion of international students came from Northern Africa and Western Asia region, accounting for 23.9% of respondents. Following closely were students from Eastern and South Eastern Asia, constituting 19.3%. In contrast, Latin America and the Caribbean, as well as Sub-Saharan Africa, had the lowest representation, with proportions of 5.2% and 8.9%, respectively.

While Muslims dominated the study with a count of 124 respondents representing 28.2%, Jews were the least represented with a count of just 4 which was less than 1% of the total number of respondents.

There were 36.6%, 26.4%, and 23.6% students with high school, bachelor's and master's qualifications respectively. Only 6.6% of the respondents had attained their PhD qualification.

As a result, majority of the students surveyed were currently reading their bachelor's, master's or PhD degrees with respective proportions of 39.8%, 26.6%, and 29.3%.

Table 1: Showing the Sociodemographic Characteristics of the Study Participants

**Variables**	**Levels**	**Frequency**	**Proportion (%)**	**Chi-square (χ^2)**	**p-value ($\alpha \leq 0.05$)**
Gender	Female	235	53.4	2.05	0.153
	male	205	46.6		
Age groups	Below 20 years	80	18.2	176	<0.001
	21-25 years	187	42.5		
	26-30 years	98	22.3		
	31-35 years	52	11.8		
	Above 35 years	23	5.2		
Region based on UN SDGs regions	Do not apply	54	12.3	72.7	<0.001
	Europe and Northern America	71	16.1		
	Northern Africa and Western Asia	105	23.9		
	Sub-Saharan Africa	39	8.9		
	Central and Southern Asia	63	14.3		
	Latin America and the Caribbean	23	5.2		
	Eastern and South-Eastern Asia	85	19.3		
Religion	Christianity	92	20.9	142	<0.001
	non-believer	115	26.1		
	Muslim	124	28.2		
	Jewish	4	0.9		
	Others	59	13.4		
	Do not wish to declare	46	10.5		
Marital status	single	350	79.5	1266	<0.001
	co-habiting	15	3.4		
	married	45	10.2		
	separated/divorced	4	0.9		
	other	17	3.9		
	Do not wish to declare	9	2		
Number of children	Have no child	395	89.8	1340	<0.001
	Have 1 child	13	3		
	Have 2 children	6	1.4		
	Have 3 or more children	7	1.6		
	Do not wish to declare	19	4.3		
Highest level of education	graduation at high school (or equivalent)	161	36.6	262	<0.001
	Barchelor's or equivalent	116	26.4		
	Master or equivalent	104	23.6		
	PhD, completed doctoral studies	29	6.6		
	Others	18	4.1		
	Do not wish to declare	12	2.7		
Level of current training	Preparatory for higher education admission	5	1.1	394	<0.001
	Bachelors	175	39.8		
	Masters	117	26.6		
	Doctoral	129	29.3		
	Post Doctoral	1	0.2		
	Do not wish to declare	13	3		

Self-health assessment of participants

International students studying in Hungary were asked to self-assess their health status and the result is presented in table 2. Before leaving their home countries, most of the students claimed their health status was very good with a proportion of 56.6%. This was significantly higher than ($p < 0.05$) those students who had bad health (0.7%) before moving to Hungary. A similar observation was made when the students first arrived in Hungary where the significant majority (47.5%) again indicated a very good health status with only 2.5% indicating a bad health upon arrival. When they were asked to rate their health status in recent period, the proportion of students with very good health status reduced to 36.2% whereas those with bad health increased to 3%. This led 28.3% of respondents admitting that their health status has slightly deteriorated since their arrival in Hungary. In contrast, a significant 43.7% indicated that their health status has not changed since they arrived in Hungary. Corona virus infection and mental health problem was the topmost health problems respondents were worried about at the time of this study. While 44.9% were concerned about corona virus infections, 41.4% worried about mental health issues. These proportions were significantly higher ($p < 0.05$) than those respondents who were concerned about acute infectious diseases (6.3%), chronic infectious diseases (3.7%), chronic non-infectious diseases (8.8%) or sexual and reproductive problems (7.7%). Majority of respondents (67.4%) had no known emerging health problems. Conversely, 22.5% had mental health problems as their emerging health problem of concern. Chronic health problem was not of concern to most of the respondents as 75.5% said they do not have any chronic health problems. Accordingly, 83.8% of respondents do not have to take any medications regularly. For those 13.7% who responded that they take medications regularly, 20.3% had stopped taking their medications because they claim it has finished and no more being prescribed in Hungary. A significant 33.8% of respondents attributed their decision to stop the medication to other reasons aside their personal decision to stop taking the medication (9.4%),

the medication not available in Hungary (9.4%) or no specialist found yet in Hungary (27.0%). Dental problem was of no concern for international students in Hungary as the significant majority (81.5%) responded no when asked if they had this health issue

Table 2: Showing Self Health Assessment of Participants

Questions	Levels	Frequency	Proportion (%)	Chi-square (χ^2)	p-value ($\alpha \leq 0.05$)
How would you rate your health (before leaving home country)	very good	246	56.6	526	<0.001
	good	147	33.8		
	fair	39	9		
	bad	3	0.7		
How would you rate your health (When you first arrived in Hungary)	very good	187	47.5	337	<0.001
	good	141	35.8		
	fair	56	14.2		
	bad	10	2.5		
How would you rate your health (Recently-the past few weeks to months)	very good	147	36.2	199	<0.001
	good	146	36		
	fair	101	24.9		
	bad	12	3		
Has your health status changed since arrival in Hungary	it improved significantly	27	6.2	225	<0.001
	it improved slightly	69	15.9		
	It has not changed at all	190	43.7		
	it deteriorated slightly	123	28.3		
	it deteriorated significantly	26	6		
What health problems are you recently worried about	Coronavirus infection (SARS-COV-2), Covid-19	193	44.9	N/A	N/A
	Acute infectious disease	27	6.3		
	Chronic, infectious disease (ie. hepatitis, HIV, etc.)	16	3.7		
	Chronic, non-infectious diseases	38	8.8		
	Dental problems	124	28.8		
	Sexual and reproductive health issues	33	7.7		
	Mental health issues	178	41.4		
	Others	77	17.9		
	Do not wish to declare	64	14.9		
Do you have any newly emerging health problem	Yes, acute physical health problem	48	11.1	N/A	N/A
	Yes, chronic physical health problem	21	4.9		
	Yes, psychical/mental health problem	97	22.5		
	No, nothing	291	67.4		
Do you have any CHRONIC health problem	yes	44	10.1	597	<0.001
	no	330	75.5		
	maybe	39	8.9		
	Do not wish to declare	24	5.5		
Do you (have to) take any medication regularly	yes	60	13.7	508	<0.001
	no	366	83.8		
	I should have taken but i do not take	11	2.5		
Why don't you take your medication if you should have	Finished and no more prescribed	15	20.3	17.1	0.002
	Have decided to stop	7	9.4		
	Medication not available in Hungary	7	9.4		
	No specialist found yet in Hungary	20	27		
	Other reasons	25	33.8		
Do you have any longstanding dental problems	yes	81	18.5	173	<0.001
	no	356	81.5		

Difficulty in accessing and utilizing healthcare services

Respondents were asked about how difficult it gets to access healthcare in Hungary and their responses are summarized in Table 3. The majority indicated that they will trust health professionals from their home countries more than those from Hungary. While 36.7% rated their trust in health professionals from their home country as 'very much', only 20.6% gave similar rating to health professionals in Hungary. The significant ($p < 0.05$) majority (36.3%) however stated that they somewhat trust health professionals in Hungary. When the need for health services arises, most of the respondents said they rather would contact their primary care providers with a significant ($p < 0.05$) proportion of 56.1% rather than contacting their religious community for advice (0.9%) or contacting a traditional healer (1.8%). Surprisingly, 63% of respondents did not have any information about the healthcare system in Hungary before their arrival. This proportion significantly exceeded the 12.9% who claimed they had such information before their arrival in Hungary.

When they were asked about getting any information on their entitlements to health services in Hungary, 35.9%, 32.9% and 31.3% respectively responded yes, no and partly. These proportions were not statistically different ($p > 0.05$). For those respondents who had information, the Tempus Public Foundation/Stipendium Hungaricum website and the university administration website were the leading online websites that provided respondents with such information. While 45.7% resorted to the Tempus Public Foundation/Stipendium Hungaricum website for their information, 41.8% depended on the University administration website. These were closely followed by those respondents who relied on their friends and family for information (39.9%), and onsite at the university administration office (22.1%). A little over half (50.6%) of the respondents said they could have full access to free public health

services in Hungary. A relatively lower proportion of 4.6% of the respondent also stated that they had to pay for all public health services while 41% of the international students surveyed have not had any health problems that required medical attention. On the contrary, 54.1% of respondents also indicated that they have been faced with health problems that required attention since they arrived in Hungary. Of the 54.1%, 27% always received the needed healthcare, 25.7% received healthcare in most cases, 11.1% in some cases, and 14.6% said they never received the healthcare services they required. Preliminary health check/aptitude test upon arrival at the university, primary care/GP services with acute problems, and visiting a dentist were the major medical care services respondents claimed had received since their arrival in Hungary with respective proportions of 38.1%, 32.8%, and 23.6%. Exactly 3.7% indicated they have had life threatening conditions and had to receive an emergency care. This proportion however was significantly ($p < 0.05$) lower than the three topmost medical care services received by international students in Hungary.

Although 44.2% of respondents indicated they felt the need for mental health counselling, 47.7% also contended that they do not need any mental health counselling in Hungary. Of the 44.2% who felt the need for mental health counselling, 31.2% did not get access to such services whereas 19.3% did get access. Consequently, only 10.6% of the respondents got the necessary mental health counselling while 33.2% did not get. In general, the proportion of international students in Hungary who are likely to face difficulties in accessing healthcare and those who are likely not to face any difficulties were not significant ($p > 0.05$). While 41.7% indicated they were likely to face difficulties in accessing healthcare in Hungary, 47.4% stated otherwise.

Table 3: Showing Difficulty in Accessing Healthcare

Questions	Levels	Frequency	Proportion (%)	Chi-square (χ^2)	p-value ($\alpha \leq 0.05$)

How much trust do you have for health professionals in your home country	very much	159	36.7	272	<0.001
	Somewhat	136	31.4		
	not really	47	10.9		
	not at all	8	1.8		
	Undecided	68	15.7		
	Do not wish to declare	15	3.5		
How much trust do you have for health professionals in Hungary	very much	89	20.6	205	<0.001
	Somewhat	157	36.3		
	not really	54	12.5		
	not at all	20	4.6		
	Undecided	100	23.1		
	Do not wish to declare	13	3		
Where do you prefer to go first for health services when you need them	Primary care (family doctor/GP) providers	243	56.1	846	<0.001
	Secondary care (outpatient clinic, or hospital) providers	86	19.9		
	Pharmacy for over-the-counter (OTC) medications	34	7.9		
	First, I use herbal medicine myself or within the family	27	6.2		
	Traditional/alternative healers	8	1.8		
	I ask the members of my religious community for advice/care	4	0.9		
	Do not wish to declare	31	7.2		
Did you have information about the healthcare system in Hungary before your arrival	Yes	56	12.9	180	<0.001
	No	273	63		
	Partly	104	24		
Did you get any information about your entitlements to health services in Hungary	Yes	155	35.9	1.43	0.489
	No	142	32.9		
	Partly	135	31.3		
How did you get information	Online, from Hungarian governmental website	70	16.8	N/A	N/A
	Online, Tempus Public Foundation/Stipendium Hungaricum website	190	45.7		
	Online, University administration website	174	41.8		
	Online, from other website	61	14.7		
	Informally, friends or family	166	39.9		
	From brochures	27	6.5		
	On-site, at university administration office	92	22.1		
	From other source	58	13.9		
Do you have access to healthcare services in Hungary	yes, full access to free public health services	219	50.8	518	<0.001
	yes partly, but I have to pay for certain public health services	133	30.9		
	no, I have to pay for all public healthcare services	20	4.6		
	no, I have access only for private healthcare	12	2.8		
	Do not wish to declare	47	10.9		
Have you been presented with any health problems since your arrival in Hungary	Yes	232	54.1	167	<0.001
	No	176	41		
	Do not wish to declare	21	4.9		
If yes, did you get the necessary care	yes, always	102	27	36.4	<0.001
	yes, in most cases, but not always	97	25.7		
	rather not, only in some cases	42	11.1		
	no, never	55	14.6		
	Do not wish to declare	82	21.7		
What kind of medical care have you taken since you arrived to Hungary	Preliminary health check/aptitude test upon arrival at the university	153	38.1	N/A	N/A
	Primary care/GP services with acute problems (ie. infections, etc.)	132	32.8		
	Primary care/GP services with chronic conditions	60	14.9		
	I visited a dentist	95	23.6		
	Out-patient care/secondary care/specialist	44	10.9		
	I had one-day surgery (without hospitalization)	11	2.7		

	In-patient care/I was hospitalized without surgery	12	3.0		
	I had surgery and was hospitalized for a while	7	1.7		
	Emergency care with serious/life-threatening conditions	15	3.7		
	I participated at preventive medical screening	25	6.2		
	Do not wish to say	99	24.6		
Have you felt the need for mental health counselling in Hungary	Yes	189	44.2	123	<0.001
	No	204	47.7		
	Do not wish to declare	35	8.2		
Did you have access to such services	Yes	81	19.3	57.9	<0.001
	No	131	31.2		
	Partly	62	14.8		
	not applicable	104	24.8		
	Do not wish to say	42	10		
Did you get the necessary mental health care	Yes	44	10.6	163	<0.001
	No	138	33.2		
	Partly	34	8.2		
	not applicable	155	37.3		
	Do not wish to say	45	10.8		
Did you have any difficulties in accessing health care (in general) in Hungary	Yes	176	41.7	97.6	<0.001
	No	200	47.4		
	Do not wish to declare	46	10.9		

Difficulties impeding healthcare access ratings

Respondents were asked to rate some difficulties that may impede healthcare access in Hungary. The result is summarized in table 4 below. For language barrier and lack of qualified interpreters, respondents rated this difficulty as very much likely to impede healthcare access in Hungary for international student with a significant proportion of 39.7% agreeing to this. While cultural barriers and misunderstandings are somewhat (30.6%) likely to impede healthcare access, religious barriers are not likely to impede healthcare access with more than half (52.8%) of the respondents attesting to this. Likewise, majority of the respondents stated that the lack of information about entitlements and lack of provider's information about entitlements will neither impede nor improve (neutral) healthcare access with respective proportions of 33.3% and 34.3%. While the majority (31.8%) agreed that the lack of translated informational materials is somewhat likely to impede healthcare access, the lack of vaccination document will not at all impede healthcare access with the significant ($p < 0.05$) majority 40.2% agreeing to this.

The administrative problems with health insurance, the lack of translated (previous)

documents, personal financial problems, unavailability of previously prescribed medications, the non-availability of mental health services and discrimination from healthcare providers were all not likely to impede healthcare access for international students in Hungary. The proportion of respondents who were like-minded to this rating were 33.0%, 34.6%, 36.2%, 42.0%, 34.5% and 33.6% respectively for administrative problems with health insurance, the lack of translated (previous) documents, personal financial problems, unavailability of previously prescribed medications, the non-availability of mental health services and discrimination from healthcare providers. On the other hand, the lack of social services for immigrants will have a neutral effect on healthcare access for international students in Hungary with 28.7% being the significant majority.

Table 4: Showing Difficulties Impeding Healthcare Access Ratings

`	Levels	Proportions (%					Chi-square (χ^2)	p-value ($\alpha \leq 0.05$)
		very much	somewhat	neutral	not really	not at all		
Estimate how much the following difficulties may impede healthcare access in Hungary	Language barriers, lack of qualified interpreters	39.7	32.4	17	5.9	5	206	<0.001
	cultural barriers, misunderstandings	12.5	30.6	28.6	18.3	10	68.5	<0.001
	religious barriers	2.6	4.6	22.2	17.9	52.8	319	<0.001
	Lack information on structure of health system	17.3	28.4	31.6	11.9	10.9	73.4	<0.001
	Lack of information about my entitlements	14.8	25.1	33.3	13	13.8	63.4	<0.001
	Lack of provider's information about my entitlements	12.1	28	34.3	12.3	13.4	85.8	<0.001
	Lack of translated informational materials	29.6	31.8	23.6	7.2	7.7	113	<0.001
	Lack of my vaccination documents	3.8	11.3	25.8	18.9	40.2	152	<0.001
	Administrative problems with my health insurance	9.4	13.2	25.9	18.5	33	71.8	<0.001
	Lack my translated (previous) documents	9	15.6	22.8	17.9	34.6	71.4	<0.001
	My personal financial problems	9.9	12.9	25.3	15.7	36.2	91.3	<0.001
	Previously prescribed medications not available	5.2	8.8	27.7	16.3	42	174	<0.001
	non-availability of mental health services	8.3	11.7	27.3	18.2	34.5	91.1	<0.001
	Lack of social services for immigrants	16.7	23.3	28.4	11.1	20.5	33.6	<0.001
	Discrimination from healthcare providers	6.2	16.2	28.7	15.4	33.6	95.3	<0.001
	Others	6.6	10.4	38.7	10.7	33.5	154	<0.001

Socio-demographics and changes in health status after arrival in Hungary

Generally, males had their health status significantly or slightly improved than females upon arrival in Hungary as presented in Table 5 (please refer to supplementary sheet). As a result, the health of females deteriorated more as compared with males upon arrival in Hungary. Those respondents in the age category of 21-25 years had their health status improved (significantly and slightly) than any other age groups. When respondents' regions based on UN SDG regions were considered, those coming from Central and Southern Asia were the significant ($p < 0.05$) majority who claimed their health status significantly improved upon arrival in Hungary. Those coming from Europe and Northern America on the other hand were the significant majority ($p < 0.05$) who said their health status either significantly or slightly deteriorated. The respondents' regions based on UN SDG regions had significant ($p < 0.05$) associations with respondents' health status rating upon arrival in Hungary. The religious background and educational level of respondents also showed significant ($p < 0.05$) associations with their health status rating. For religion, the significant majority who claimed an improvement (both significant and slight improvements) in their health status upon arrival in Hungary were Muslims. In contrast, non-believers were the major group whose health status deteriorated significantly or slightly upon arrival in Hungary. When their level of education was considered, those with high school graduation certificate or equivalent had their health status significantly deteriorated than any other educational levels. On the other hand, the significant value ($p < 0.05$) majority of respondents with bachelor's degree or equivalent had their health status significantly improved upon arrival in Hungary than any other educational levels.

Please, refer to supplementary sheet for

Discussion of findings

We conducted a survey to gain an insight into the state of health of international students in Hungary in the midst of the Covid-19 pandemic. This cross-sectional study helped identify the challenges foreign students encountered and how they adapted to their new environment for academic gains. The findings of the study brought an exposition to valuable information surrounding how their healthcare needs were met. In this discussion, we dilate on the issues raised and compare with existing literature, making recommendations for greater benefits under the following subheadings:

Health status by socio-demographics

Gender differences of participants showed traits of resilience at withstanding migration fatigue in favour of males. As shown in Table 5, female participants who suffered health breakdowns were more than their male counterparts. This finding reverberates the work of Trappolini and Giudici [32] who lend credence to the observation that migration affects the health status of migrants after staying in a foreign country for long. **Health status differences** between migrants and non-migrants are marginally more distinct among women than men. This led some researchers to posit that there are factors responsible for the unique trajectories travelled by migrants depending on their gender [33]. Their assertion was confirmed in another study that went ahead to mention that the disadvantage suffered by women was as a result of insufficient attention to preventive and screening measures for morbid medical conditions such as metabolic diseases, obesity and perinatal problems [34]. Apart from ailments common to both gender such as infectious diseases, female migrants may encounter additional difficulties in accessing health care because they are mostly relegated to the background for socio-cultural reasons [35]. More so, women by virtue of their vulnerable status as migrants become exposed to numerous health risks of which physical abuse and sexual violence are but a few [36]. Narrowing down into the health problems that were of concern to students in Table 2, 33 of

them representing 7.7% indicated sexual and reproductive health issues. Of this figure, 19 (4.4%) were males and 14 (3.3%) were females. Reasons were not given for the concerns as this was a close-ended question. However, existing literature explains that male international students are more sexually active than their female counterparts hence stand a higher risk of exposure to sexually transmitted infections [37]. This revelation could be lending clues to their vulnerability to indulge in risky sexual behaviours under the influence of alcohol and cigarette smoking. For obvious reasons, these behaviours are common and may possibly occur without the use of sheaths or condoms to protect them from the body fluids of their sexual partners [38] [39].

On the flip side, female international students may be vulnerable to violations of their feminine rights as in exercising their rights to negotiate and participate in safe sexual activities. The fear of associated risks to contraction of sexually transmitted infections and unwanted pregnancies could be triggers to disruption in their mental processes [40]. More disturbing is the fear of being stigmatized after falling victim to indiscriminate sexual violence due to socio-cultural norms that frown on females being openly expressive in their sexual exploits [41]. International students are reported to be aware of protective measures in avoiding all forms of sexual mishaps. Strangely, these mechanisms are underutilized for sake of culture. While sexual and reproductive units of student clinics may be at their disposal, they may not patronize those facilities because they do not want to be part of the statistics [42]. A number of studies have supported this fact and these are some of the reasons mental health services should be better resourced to provide unlimited guidance and counselling services to all manner of students [43] [44]. This evidence inevitably accounts for the differences in gender susceptibility to poor health as documented in this study. Shifting the perspectives in the shade of participants' region of origin, those from Central and Southern Asia experienced an improvement in health while in Hungary. Regrettably, the ones from Europe and Northern

America reported a deteriorated health after relocating to Hungary. This is unfortunate Eastern Europeans are reported to have the highest odds ratio of poor health with respect to nativity [45]. Some other reasons for this outcome could be as a result of the variations in how Coverage UHC is perceived and implemented in these destinations. Almost every country in Western Europe is said to have UHC [46]. Romaniuk and Szromek (2016), revealed there are nuances in how health reforms have transitioned over the years toward UHC in Central and Eastern Europe bringing the countries of the former Soviet Union in perspectives with focus on the near similarities and subtle differences in their health systems. With this evidence, the idea of UHC in North America will be different from how it is interpreted in Hungary and this may affect the health of students from these origins who have come to study in Hungary. Therefore, this problem of deciding whether a given country has, or does not have universal health coverage is perpetuated by the lack of precision in defining its scope. In effect, disparities within regions of origin could be partly due to differences in the culture of the people [47].

In terms of education, respondents with high school certificates experienced poor health relative to respondents on other educational levels. This finding amplifies a study carried out on the behavioural health risk among international students in the United States and how they built resilience to protect themselves. The study, conducted in the United States examined the sociodemographic differences that served as pointers to acts of resilience or vulnerabilities in the acculturation process. It found that graduate students usually exhibit greater levels of resilience than undergraduates, primarily because previous experiences have added to their level of maturity [48]. This finding is an extra affirmation substantiating the earlier works of Panter-Brick et al (2014) on the application of concepts to health, risks and resilience indicating an inclusive approach to viewing them shape the pathways of human experience rather than treating them in split opinions.

[49],

Scaling the discussion in the context of religion, the health of Muslims improved while that of non-believers deteriorated. Religious beliefs may not be empirically proven. Nevertheless, they have an impact on those who practice them. According to Karl Marx, religion is *"a fantasy that allowed people to balm their degraded lives."* In an elaborate expression, Marx stated *"Religion is the sigh of the oppressed creature, the heart of a heartless world, and the soul of soulless conditions. It is the opium of the people."* Religion moulds beliefs and behaviours of practitioners and has been confirmed that religiously practicing its doctrines improves the impact of health interventions [50]. The Islamic faith incorporates concepts of health beliefs, health tips and social care that is integrated into practice [51], and even promote mental health after traumatic experiences [52]. Non-believers, on the other hand hang on no supreme force as backup for encouragement hence their inability to resist stress hence the deterioration of their health as reported by these authors [51]. For ages, people have relied on religion for hope and worthiness in times of ill health, trusting that there is a profound sense and purpose to overcoming physical and mental difficulties. In the circles of professional nursing, the spirituality of man has been greatly considered as an element to be held in full regard if their healing process is valued. It is a significant piece of holistic patient care towards recuperation. In fact, it has been emphasized in palliative care that when people are faced with fear of the unknown or are at a point of imminent death, many resort to religion for reassurance [53]. It is understandable therefore that the practice of religion has influence over how people reacted to the pandemic and escaped its harsh effects. Research continues to reverberate that persons who exercised faith in their religion habitually demonstrated greater resilience to recover from their illnesses and make new adjustments to cope with life. [54]. That irrespective, the debate over religion and improved social systems still take the centre stage in national development. For instance, there are societies that give excuses against medical treatments and public health interventions while others embrace the idea with zeal. A **classic example is observed in the**

survey that was conducted in Poland among two groups of residents with data gathered during two distinct windows of the pandemic notably (1) **the final period of the third wave of the Covid-19 pandemic labelled "pandemic group,"** and (2) **the post-pandemic period** labelled **"post-pandemic group." Results showed no significant differences in accessing healthcare among the two categories. They concluded systemic factors were better involved** [55]**.**

It has thus been proposed that while acknowledging religion, its role in modelling attitudinal change toward healthcare, the essentials of an all-inclusive healthcare service is driven by a multiplicity of factors and not merely the existence of a pious distinctiveness [56]**.**

To summarize the analysis rendered under socio-demographics, differences in gender, origin, educational and religious backgrounds had an impact on participant's health after migrating to Hungary. This could be as a result of their physical and psychological make-up reacting to their adaptability in adjusting to their new environment. This outcome is not unexpected since participants are coming from different parts of the world with different experiences. These differences will determine whether they would be able to survive in a new terrain and how much of the differences they can withstand, then how much of it they can also sustain over time. In the context of existing literature, sociologists have explained associations between demographic characteristics, sociocultural adjustment, and psychological well-being accounted for changes in different sociocultural spheres of growth along the life course and were the most precarious for the psychological well-being of immigrants in the absence of apparent discrimination, taking into account predictions of depression from participants [57] Consolidating our finding is the conclusion drawn by Counted (2018), [58] in his work on religion and place attachment throwing light on the role of religious and spiritual experiences in people's understanding of their environment. He argued that sociodemographic factors remain important variables for discussing the sense of place theory, a disposition that was

upheld by the investigations of Taylor, Everett and Edgar (2021), that ethnic factors are associated with attitudes displayed towards specific geographic settings of migrants hence not a one-size-fit all situation [59].

Medical services patronised since arriving in Hungary

Considering the kind of medical services sought by students as captured in Table 3, visits to the GP were mainly because of infections (132, 32.8%) and chronic illnesses (60, 14.9%). Juxtaposing this proportions to the total number of participants, close to 50% of international students had health issues after settling in Hungary, an outcome synonymous with existing literature from Oduwaye, Kiraz, and Sorakin, (2023), [60]. Proponents of migration health have long advocated interventions for persons in motion over long distances with limited access to healthcare services. They further buttressed their stance not only in line with philosophies underpinning anthropological reasoning but also with empirical evidence of changes occurring in the human body during the course of migration. Unfortunately, the gap between health promotion and disease prevention activities remains wide among vulnerable populations [61]. Reasons accounting for the high numbers of infection can be attributed to Covid-19 pandemic sufficing it to be that the baseline data of international students were established at the time they arrived in Hungary. A situational analysis could also be made in the context of how much preparation was made by host institutions towards receiving students especially in the face of a global outbreak of an infectious disease [62]. The adequacy of how much personal space each student had to themselves in their various dormitories or apartments of residence, lecture rooms or theatres may have also provided the vehicle that worsened the already volatile contagious situation. Guidelines of the World Health Organisation clearly specify among others the preparation of public health responses to migrant arrivals, while continually meeting the health needs of existing migrant populations and the receiving community [63]. Advancing the argument towards an infallible culture of hand hygiene where resources were mounted by

institutional managers, questions could be raised in the direction of whether compliance with their usage was supervised or left to rot in the decadence of non-usage [64]. Installations of hand-hygiene stations at designated points in hospital areas and other public places in respect of protocols developed to combat Covid-19 pandemic were undoubtedly a proof of institutional managers' initiatives born out of commitment to state protocols mechanised to control infection spread. Situations might have also been compounded by the unexpected influx of a marauding crowd spilling over from the Rusian-Ukranian war when Hungary in that trail-blazing humanitarian gesture gave room to the masses fleeing an acquisitive pandemonium [65]. Records even confirm that Ukraine has lower SARS-CoV-2 vaccination rates than Hungary hence a greater possibility that some spread must have resulted from that emergency infiltration [66]. Public health specialists may argue that Hungary had a strong heard immunity per the proportion of its population that got vaccinated and therefore mounted a strong garrison against infection spread from across its borders. However, foreign students were expected to champion their own welfare by securing personal hand sanitizers and nose masks as self-guard 'weapons' against the viral infection and other infectious microorganisms [67]. In that understanding, some blame may be apportioned to students for the laxity on their part in complying with global directives in the wake of the pandemic. This finding partly agrees with the work of Jaita, Pisutsan, Lawpoolsri et al (2023), that concluded international students' knowledge about infectious and non-infectious diseases were woefully inadequate hence the need for migrant students to revise and repackage preparative efforts at disease prevention before embarking on a travel mission [68].

Important in this discussion is how much impact was made if per WHO regulations all international students were vaccinated against the infectious disease, how many shots of the vaccine they had and whether they received a booster dose in addition [3]. Fundamentals of disease surveillance and response preparedness systems may also need to be re-assessed and

re-evaluated for effectiveness as part of a problem-solving approach in answering questions related to the high number of infections [69]. Unfortunately, commitment to this activity is scarce, according to relevant literature [70]. Of equal importance are the numbers of international students who presented with chronic disease conditions. Migrants living with chronic diseases are often disadvantaged as chronic illnesses require a lifetime medical treatment management for optimal health and delays in accessing timely care is a way of silently courting complications [71]. The situation as it stands, paints a gloomy picture of the health needs of international students. This finding complements the observation of Mucci Traversini, Giorgi, Tommasi, Sio, and Arcangeli, (2020) [72], who after their systematic review on the psychological health of migrant workers uncovered that disorders emerging chiefly from the research were low concentration at work, depressive moods and anxiety. These were conditions arising from marginalization amongst others, for which commitment to occupational medicine would be a wholesome remedy in vanquishing those occupational triggers.

Making space for diversity and integration

From Table 4, the estimation of cultural barriers and misunderstandings becoming an impediment to healthcare access in Hungary was tipped for ‘very much’ at 12.5% and somewhat at 30.6%, altogether 43.1%. This figure is greater than the stakes for ‘neutral’, ‘not really’ and ‘not at all’ (17%, 5.9% and 5% respectively). The expression of international students’ opinion regarding this outcome simply communicates their desire of seeing and benefiting from a multi-culturally oriented health service when they need them. Hitherto, they had indicated by popular acclamation that they trust care professionals in their home country more than they do for caregivers in Hungary (Table 3); a clue for Hungary to run with in birthing its own success of an enviable health hub within the EU that will draw not only the

patronage of international students but the broader community of Europeans within its sub region. There is diversity and lack of integration regarding health care provision across Europe due to policy differences between health care systems and social services leading to poorly coordinated outcomes [73]. But migrants are also human beings and have the fundamental human right to enjoying the highest attainable standard of health [3]. Migrants in general are susceptible to chronic physical health conditions and mental health [74]. For this reason, the WHO charges all countries to build robust and resilient health care of sound quality that responds to the needs of all members in their population, including those who may be vulnerable such as refugees and migrants [63]. Healthcare that is comprehensively and equably reachable must be provided for these migrants in national contexts and in response to intricate and emerging individual needs [75]. The commitment towards actualising this goal is the heartbeat of the World Health Organisation in the European region, even to the point of demanding the inclusion of undocumented migrants in an integrated healthcare plan for the entire population [76]. Lack of funds and of trained and stable workforce, organisational shortfalls and poor synchronisation of activities across the different horizons of management hinder the provision of healthcare for migrants [77]. Reflecting over the high numbers of international students in dire need of healthcare in the midst of such rich and caring continental provision, more will have to be done for the populace of foreign students by the Hungarian institutions as a matter of duty and global prospect.

Critically examining the statistics

Remarkably, the health of respondents within the age bracket of 21-25 years improved with their stay in Hungary while that of older ones deteriorated. Facts supporting this observation have been linked to the boisterous and adventurous nature of age group 21-25 years who have less social responsibilities. On the reverse, older ones are focused on their goals but also prone to social exclusion for various social reasons. Converse to this finding is the evidence provided

by [78] that young adults schooling in host nations have demonstrated strength in being responsible for themselves rather than being wholly dependent on others as compared with care leavers of the same age group. They have also been identified to suffer less psychosomatic distress and fewer risky behaviours compared with care leavers and that the declining state of health in older migrants has been attributed to sub-standard living conditions. [79] observed similar findings in their study and emphasised the importance of putting the life course in view as an essential reference point so that issues about it can be accurately and fairly judged. Appreciating the pattern of the aging process in policy reviews informs what interventions will promote health and tackle social exclusion for older migrants. Furthermore, squirting a dose of empathy in social exclusion activities will be advantageous for grown-up migrants. [80], eruditely upheld this viewpoint and further crystallised a four-point direction that illuminates how this knowledge can be applied to (i) effectively respond to new health trends and evidence, (ii) fill longstanding gaps in care, (iii) addressing fully issues of health inequities, and (iv) helping produce efficiently using less of resources. This direction has been predicted to prevent or reduce adverse outcomes if optimised with care [81]. Integration into development oriented societies that embrace diversity and thrive in unity becomes the ground for which older migrants can wilfully be postured to pivot so they can contribute their wealth of experiences for collective benefits [79]. Galvanising this blueprint, Gullo, García-Alba, Bravo, and del Valle, (2021) [82] emphasised a more cohesive, multidisciplinary rallying that will reflect over the life course. In so doing, we soften and even out the jagged edges of a lost roadmap. In principle, education, according to the United Nations Development Program (UNDP), a great equalizer, is a major prognosticator of health and healthy outcomes that fosters social cohesion for a superior economic advantage [83].

Switching into the lane of Table 2, self-ratings of their health saw a steady decline in the numbers for ‘very good’. The interpretation for this scenario could be that international

students generally arrived in Hungary in good health. From an initial 56%, it dropped to 47.5% upon arrival in Hungary and 36.2% close to the time of responding to the questionnaire. Largely, the decline in health occurred over the period in perspective (during COVID-19 pandemic) consequently resulting in the troubling numbers seeking care service. Related studies sourced from scholarly bulletins amplified the works of many researchers that international students were overwhelmed by innumerable mental health challenges during the Covid-19 pandemic due to systemic factors. These challenges were suspected to be spilling from academic or socio-cultural sources [84].

Difficulty in accessing healthcare- the discipline of equity

The findings in Table 3 echo a void in the target of having every member within the international student community satisfied with the freedom to access healthcare at their own will when deemed right by them. While some got it always and others had it in most case and some others never had a chance at it. More worrying is the realisation of the staggering 14.6% students who never got the services they required registering an outright contradiction to the WHO's fight of leaving no one behind as far as universal health coverage is concerned [3].

By design, several factors have been implicated as common hitches that buffet international students. It has been captured in similar studies which found that the needs of international students were not adequately addressed. For instance, a study was conducted by Kosztin, Merkely Szabó et al [85] with the aim to compare the prevalence of acute infection and seropositivity of SARS-CoV-2 among healthcare workers (HCWs) and medical students. A total of 1832 of students recruited in a study (53%) were international students, while 682 (20%), Hungarian students. Hungarian students had lower prevalence of seropositivity. High prevalence was recorded among international students. By the proportions, an assumption can be made that more international students would need a doctor's attention than domestic

students would [86]. In effect, seeking medical attention is the fulcrum around which recovery evolves. Unfortunately, it appeared affected students encountered some obstacles in reaching out for these services. A simple prediction of the underlying cause may be alluding to bureaucratic and management issues rather than geographical or infrastructural causes [87]. Deprivation suffered by the overwhelming numbers may have unleashed a greater burden of the contagious respiratory infection on the population. In the face of a worsening wave of an already devastated world, compromises had to be made in certain critical areas. A call was made to suspend bureaucracy in the provision of essential services during the pandemic since its time-consuming. The action was a part of global efforts to nib the spread in the bud [88]. One negative element that rears its head in any multicultural setting is the possibility of an unwanted duo- racial and ethnic discrimination. The two cannot be totally ruled out while interrogating the reasons for which those concerned were not given the chance if the results are something to go by. Scientific literature is replete with information on the subject matter. Certain foreign cultures have preferences for particular nationals while expressing resentment toward others Authors have remarked saying although instilling moral values in students is appropriate, strategic investment for futuristic goals of a *healthy population is necessary to sustaining multiracial student communities [84].

If we are to refer to the consequences of systems operating in deficits, chronically ill participants had a disruption in the continuity of their medical treatments either because those medicines were not available in Hungary or there were no specialists to attend to them (access to GP services appeared unavailable). The potentially dangerous situation places affected students in limbo- a point of retardation [89]. Managers may need to be reminded that exposure to adverse societal pressure increases one's vulnerability to deteriorating mental health throughout their lifespan due to systemic racism [90]. In another vein, others would deny group discrimination in order to avoid the psychological pain associated with victimhood therefore

isolating their group from the afflictions of stigmatized racial minorities [91]. More intricately, adverse individual determinants, internal forces of an organisation and prevailing market forces create vulnerabilities among migrants [92]. These distinct phases of migration are linked to specific vulnerabilities that have the potentials to influence health outcomes in later life. Thus, pre-existing health problems at the pre-migration phase may blight one's state of health during migration, which may subsequently be aggravated by somatic or mental trauma, injury, or deprivation during the process of migration [93]. Comparatively, a yawning gap exists between the ideals of global health targets embedded in SDG-3, (improved health for all in the broader scope) through universal health coverage and the prevailing situation of international students at the time. The tenets of universal health coverage reiterate health as being a fundamental human right and commits to maximising chances at securing the highest attainable level of health for all since anything short of that drowns this global effort [94].

Levelling off the demands of an international student population, health services require government's commitment to healthcare resourcing. This action will provide the channel for ironing out all difficulties associated with access be it geographical, financial, infrastructural or human. In the light of this outcome resonates similar findings by Chen et al, 2023, highlighted strategic positioning of health centres within communities improve healthcare access and that scaling up their numbers with effective coordination could enhance healthcare outcomes [95] In more robust health system settings, WHO inspires public health impact through policy dialogue and tactical avenues that support improved performance for collective benefits [3]. Bringing into practice this foresight means expanding health services for populations in entirety with focused interventions for international students in all facets applicable.

Limited insurance coverage

Health insurance solves the problem by half covering the cost of consultation, examination and diagnostic procedures as indicated in the Stipendium Hungaricum scholarship package for all beneficiaries. The absence of an insured health simply means inaccessible healthcare [96]. From the Hungarian central statistical office, 11.4% of the entire student population in institutions of higher education are international students. Out of this, 4.4% are Stipendium Hungaricum scholarship holders. This implies that a significant 7% of foreign students may either be beneficiaries of other forms of scholarship or self-funding. Secondly, self-funding students are by regulation under obligation to insure their health during the admission process. Deductively, some form of insurance coverage must have been in place for every single student. Therefore, the ‘obstructed’ access to services for international students depicted in Table 3 could be related to reasons other than health insurance coverage [97]. As a minority group in a foreign country, coverage of the population concerned and the kind of services accessible to members are usually limited as revealed in current scientific literature [98]. It has also been on record that without health insurance, it is usually difficult and almost impossible for students to bear the full cost of cash and carry- the reason for which institutions arrange for affordable insurance services for their students [99]. It is a global phenomenon that visiting a specialist and receiving some treatment could cost so much dollars in the United States while a hospital stay of only three days could cost about 10,000 dollars [100] [101]. Interestingly, the average cost of healthcare in Hungary for international students is between 200-300 EUR per year. Relatively, Hungary offers an affordable alternative; nonetheless, it could be challenging for a lot of foreign students. Weighing the options available, consequences for a denied access are ominous and devastating since essential services like vaccinations and management of critical conditions risk being missed and complicated [102]. The TAJ card however covers basic ophthalmic and dentistry services such as consultation and examination

fees but not for prescriptions for lenses or aesthetic procedures thereby precipitating a limiting factor to accessing complex services [103].

Communicating on chaotic wavelengths

The stakes were high (39.7%) on the Likert scale in estimating that language barrier and the lack of qualified interpreters as impediments to accessing healthcare in Hungary. Study participants indicated language was their most significant barrier to accessing care through the ratings of the possible options among 14 other variables in its category. It has been noted in other studies that suggest international students were not able to effectively communicate socially and academically, a situation that retarded their ability at acquiring new conversational skills in their host country. This assertion was corroborated by [104] who conducted a study on language-related problems of international students in a Russian university and concluded that international students in non-English speaking countries contend with the challenges of language difficulties. The Hungarian society is mostly condensed in their local language. A significant 98.9% speak Hungarian, English 25.3%, German 12.6%, Russian 2.1%, French 1.5%, Romanian 1.4%, other 5.1% ([105]). Unfortunately, the process of patient-caregiver communication is hampered by a strong language barrier. This deficit negatively impacts the quality of care that is rendered or received [106]. There are potential risks to students' health if their health context, language, and beliefs differ significantly from the understanding of health in the Hungarian context. Misunderstandings can occur and delays in diagnoses pose a threat to their health and safety unless assistance is sought from people who speak both languages. Until then, the integrity of privacy and confidentiality may be seriously compromised [107]. Resorting to medical interpreter services agreeably may be a reasonable means [108]. However, to correct this cacophony, we should be making use of language translator applications that suit this digital dispensation [109].

The findings of this study bear consequences for both the international students' community and their host country Hungary. Introducing a mandatory language training for international students in the first 6 months of their studies as it is with countries like Germany [110].

Conclusion

This study highlights major difficulties encountered by international students in Hungary while navigating the intricate healthcare system of Hungary. From the findings of the study, international students continue to battle with access to healthcare in Hungary as it is in other parts of the world. While geographical location may remain the primary factor for this impediment, the lack of access due to inharmonious linguistic deficiencies between students and caregivers creates a crucible of tension further sinking the attempts of many foreign students in need of medical attention. To continue in this trajectory will only thwart global efforts at improving health outcomes. The impression created by this data portrays access to health services as an atrium type space in the middle of a wide corridor. This research is expected to help in reviewing policies and developing significant community partnerships that foster evidence-based interventions. More importantly, it should be embraced in the strength of neuro-divergent harmony without acrimony or animosity for an affirmative action that nurtures a home away from home kind of environment for international students in Hungary. In summing up, a future qualitative study will be a booster to complementing this survey.

Limitations of the study

Appreciating the commitment involved in this study, we are hopeful the findings will positively impact the interests of international students in multiple areas especially in the advancement and enlightenment of their entitlements to the best patient-centred healthcare. That notwithstanding, we acknowledge that our findings may not necessarily represent the exact situation in all other institutions of higher learning in Hungary. Our study also featured only

international students with no concession to health caregivers. We do also acknowledge the limitation of not collecting data on the exact immigration periods of our respondents, realising that could have offered us a chance to disaggregate and zoom in on some details. Finally, the unequal representation of religious groups for a balanced quorum on issues of religiosity was not a deliberate act but rather the exact reflection of the study population.

References

[1] World Health Organization, “Mental Health care for all: let’s make it a reality.,” WHO, 2021.

[2] S. S. B. V. a. S. Rita Baeten, “Inequalities in access to healthcare. A study of national policies,” 2018.

[3] W. H. Organization, “COVID-19 Vaccination Insights Report,” WHO, 2024.

[4] J. &. P. I. (. Cylus, “An analysis of perceived access to health care in Europe: How universal is universal coverage?,” *Health Policy,* vol. 119, no. 9, pp. 1133-1144, 2015.

[5] Tempus Közalapítvány, “Stipendium Hungaricum - Statisztikak,” Tempus Kozalapitvany, 2022.

[6] L. R. F. G.-O. S Hungler, “‘Hungary: Legal Response to Covid-19’, in Jeff King and Octávio LM Ferraz et al (eds),,” The Oxford Compendium of National Legal Responses to Covid-19 (OUP 2021). , 2023.

[7] I. O. f. Migration, “The European Migration Crisis and Hungary,” IOM UN MIGRATION, Budapest, 2025.

[8] A. S. L. Z. C. László Zoltán Zöld, “The migratory impact of COVID-19: The role of time and distances in the migration decisions of Hungarians during the COVID-19 pandemic,” *Wiley Online Library,* vol. 30, no. 8, p. 2804, 2024.

[9] K. L. I. P. S. S. R. I. Hilal S, “Psychological Distress and Food Insecurity among International Students at a Hungarian University: A Post-Pandemic Survey.,” *nutrients,* vol. 16, no. 2, p. 241, 2024.

[10] T. Bianka, “The Effect of the Pandemic on the Internationalisation of Higher Education.,” *TEMPUS KOZALAPITVANY,* 2021.

[11] L. Gall, “Hungary’s Xenophobic Anti-Migrant Campaign,” *Human Rights Watch,* 13 September 2016.

[12] V. G. S. S.-N. A. A.-T. A. B. S.-A. M. &. K. L. R. (. Rekenyi, “The Effects and Differences of Social Support, Depression, and Vital Exhaustion during the COVID-19 Pandemic among International and Domestic University Students,” *International Journal of Environmental Research and Public Health,* vol. 20, no. 2, p. 1502, 2023.

[13] H. C. S. Office, “Foreigners residing in Hungary,” Hungarian Central Statistical Office, 2021.

[14] C. d. R. C. E. A. V. C. O. G. A. a. S. C. “. A. t. G. M. i. H.-I. P. J. N. Velásquez, “Multimethodological Approach to Gastrointestinal Microsporidiosis in HIV-Infected Patients.,” Acta parasitologica,, 2019.

[15] G. &. E.-N. 2. Priebe, “Public Health Aspects of Mental Health Among Migrants and Refugees: A Review of the Evidence on Mental Health Care for Refugees, Asylum Seekers and Irregular

Migrants in the WHO European Region," Health Evidence Network Synthesis Report, Copenhagen: WHO Regional Office for Europe;, 2016.

[16] B. S. Szaflarski M, "The Effects of Perceived Discrimination on Immigrant and Refugee Physical and Mental Health.," *Adv Med Sociol. ,* vol. 19, pp. 173-204, 2019.

[17] I. O. f. Migration(IOM), "World Migration Report," IOM UN Migration, 2022.

[18] University of Pecs, " Information about PTE GP,," University of Pecs, Accessed April, 21, 2024.

[19] University of Pecs International Centre- PTE, "Medical Care," [Online]. Available: https://international.pte.hu/current-students/information-about-services/medical-care. [Accessed 17 July 2025].

[20] R. E. D. A. D. H. &. M. J. R. Ashcroft, Principles of Healthcare Ethics, 2007.

[21] OECD, "Education at a Glance," OECD Publishing, 2024.

[22] OECD, "Education at a Glance," OECD Publications, 2022.

[23] I. C. Insurance, "European Health Insurance Options," [Online]. Available: https://www.internationalinsurance.com/health/europe/?srsltid=AfmBOopD88Ss8fz0bOUGzUajrWoo4zifl_7bROtUcSttCMul_eEUZKeF. [Accessed 17 July 2025].

[24] April International, "Hungary Health Insurance 2025 - For Foreigners + Expats," ONLINE, Accessed April, 21, 2024.

[25] April International, "April International-The April Group," [Online]. Available: https://www.april-international.com/en/about-april/april-group. [Accessed 17 July 2025].

[26] M. Swan, "Crowdsourced Health Research Studies: An Important Emerging Complement to Clinical Trials in the Public Health Research Ecosystem," *JMIR,* vol. 14, no. 2, p. e46, 2012.

[27] World Health Organisation, "History of WHO- Global Health Organisation," WHO, 2025.

[28] R. Z. C. X. P. P. E. I. C.-M. I. A. R. W. Burns, "Migration health research in the United Kingdom: A scoping review," vol. 4, 2021.

[29] J. V. A. B. Z. C. R. a. M. K. K. Wickramage, "Migration and health: A global public health research priority,," *BMC Public Health,* vol. 18, no. 1, 2018.

[30] S. V. &. Z. Bács, "International students in Hungarian higher education.," *Applied Studies in Agribusiness and Commerce.,,* vol. 4, no. 13, 2020.

[31] T. Bajzat, “INTERNATIONAL STUDENTS’ INTERCULTURAL CHALLENGES IN HUNGARY,” *Multidiszcip. tudományok,* vol. 13, no. 3, 2023.

[32] C. G. Eleonora Trappolini, “Gendering health differences between nonmigrants and migrants by duration of stay in Italy,” *Demographic Research,* vol. 45, no. 221–258 , 2021.

[33] E. T. &. C. Giudici, “Gendering health differences between nonmigrants and migrants by duration of stay in Italy,” *Demographic Research,* vol. 45, no. 7, 2020.

[34] M. a. M. G. Khlat, “Health and Mortality Patterns Among Migrants in France.,” *University of Pennsylvania,* vol. 8, 2017.

[35] K. a. G. D. Donato, “Gender and international migration.,” *Russell Sage Foundation.,* 2015.

[36] S. R. C. E. &. L. T. Lattof, “Priorities and Challenges Accessing Health Care Among Female Migrants,” *Health services insights,* vol. 11 , 2018.

[37] A. K. H. L. P. M. P. G. Maina, “Abukari Kwame Hua Li Pammla M. Petrucka Geoffrey Maina Sexual and Reproductive Health Experiences of International Students Studying in Universities of Western Countries: A Critical Literature Review,” *Abukari Kwame Hua Li Sexual and Reproductive Health Experiences of International Students Studying in Universities of Western Countries: A Critical Literature Review Journal of International Students ,* vol. 14, no. 2, 2024.

[38] A. M. A. B. F. E. C. B. S. F. E. D. J. A. A. …. D. J. A. Mundie, “University students’ access and use of sexual and reproductive health services in Australia.,” *Culture, Health & Sexuality, ,* vol. 1, no. 17, 2024.

[39] B. C. E. C. S. Sudarto, “Exploring Barriers and Opportunities for Tertiary Education Providers to Deliver Sexual and Reproductive Health Education to International Students: A Qualitative Study.,” *Sex Res Soc Policy,* 2025.

[40] H. M. A. S. M. e. a. .. Ouahid, “Gender norms and access to sexual and reproductive health services among women in the Marrakech-Safi region of Morocco: a qualitative study.,” *BMC Pregnancy Childbirth,* vol. 23, p. 407, 2023.

[41] S. I.-W. D. &. Y. S. Okeke, “Adolescent pregnancy in the time of COVID-19: what are the implications for sexual and reproductive health and rights globally?,” *Reprod Health ,* vol. 19, no. 1, 2022.

[42] J. Y. N. N. C. e. a. Okyere, “Adolescent sexual and reproductive health needs and utilisation of health services in the Bono East Region, Ghana.,” *Ghana. Reprod Health ,* vol. 21, p. 87, 2022.

[43] B. P. &. M. Todorova, “Sexual Violence Prevention and International Students in Canadian Universities: Misalignments, Gaps, and Ways Forward.,” *Todorova, Brooks, Persaud, & Moorhouse, (2022). Comparative and International Education/Éducation comparée et internationale. ,* vol. 50, no. 2, pp. 33-, 2022.

[44] A. P. H. K. F. e. a. Bakhtiari, “Factors affecting students’ attitudes towards reproductive health in the north of Iran: Designing an educational program.,” *Factors affecting students’ attitudes*

towards reproductive health in the north of Iran: Designing an educational program. BMC Public Health , vol. 23, p. 1557, 2023.

[45] D. &. B. O. Lanari, “International migration and health inequalities in later life.,” *Ageing and Society,* vol. 32, no. 6, 2012 .

[46] G. P. Marchildon, “ The integration challenge in Canadian regionalization.,” *Cadernos de Saúde Pública 35 (suppl 2): ,* vol. 35, no. 2, 2019.

[47] P. S. A. T. e. o. t. h. s. o. i. C. a. E. E. a. t. a. w. s. e. a. p. f. a. a. o. 2. y. o. t. B. H. S. R. 1. 9. (. Romaniuk, “Romaniuk, P., Szromek, A.R. The evolution of the health system outcomes in Central and Eastern Europe and their association with social, economic and political factors: an analysis of 25 years of transition. BMC Health Serv Res 16, 95 (2016).,” *Health Serv Res ,* vol. 16, p. 95, 2016.

[48] A. M. C. L. X. D. a. Z. Youn Kyoung Kima, “Behavioral Health Risk and Resilience,” *ournal of International Students,* vol. 9, no. 1, 2019.

[49] B. A. E. M. M. F. P. K. L. J. Panter-Brick C, “Practitioner review: Engaging fathers--recommendations for a game change in parenting interventions based on a systematic review of the global evidence.,” *J Child Psychol Psychiatry.,* vol. 50, no. 11, pp. 187-212, 2014.

[50] M. H. E. P. M. J. &. R. T. Helen McLaren, “Review of Religious Considerations in Interventions with Muslim-Minorities in Australia,” *Journal of Religion and Health ,* vol. 63, no. 2031 - 2067, 2024.

[51] W. S. I. &. K. G. Shahin, “Health beliefs and chronic illnesses of refugees: a systematic review.,” *Ethnicity & health,* vol. 26, no. 5, 2021.

[52] U. F. A. L. A. P. F. &. R. T. EL-Awad, “ Different relations of religion and mental health: Comparing Middle Eastern Muslim refugee and immigrant adolescents.,” *European Journal of Health Psychology,* vol. 29, no. 1, pp. 26-37, 2022.

[53] N. I. C. R. D. M. M. R. G. S. A. S. a. R. D. i. N. A. S. R. H. (. 2. Murgia C, “Spirituality and Religious Diversity in Nursing: A Scoping Review.,” *Healthcare (Basel). . ,* vol. 10, no. 9, p. 1661, 2022.

[54] R.-K. S. A. B. Janzen KC, “Nurses' Perspectives on Spiritual Caregiving: Tending to the Sacred.,” *Journal of Christian Nursing : a Quarterly Publication of Nurses Christian Fellowship. ,* vol. 36, no. 4, pp. 251-257, 2019.

[55] M.-K. M. B. E. Tuczyńska M, “Religious affiliation and perceptions of healthcare access during and after COVID-19 in Poland.,” *Front Public Health. 2025 ,* 2025.

[56] N. Lalani, “Meanings and Interpretations of Spirituality in Nursing and Health. Religions, 11(9), 428.,” *Religions, ,* vol. 11, no. 9, p. 428., 20.

[57] I. &. J. N. Shekriladze, “Shekriladze, I., & Javakhishvili, N. Sociocultural predictors of immigrant adjustment and well-being.,” *Frontiers in sociology,* vol. 9, 2024.

[58] F. W. V. Counted, “Religion and Place Attachment: A Cascade of Parallel Processes,” *The Psychology of Religion and Place,* vol. 3, no. 030, 2018.

[59] Y. E. A. M. &. E. F. Taylor, “Perception of cross-cultural adjustment by immigrant professionals from three ethnic groups in one host context.,” *International Journal of Cross Cultural Management,* vol. 21, no. 2, pp. 227-244, 2021.

[60] O. K. A. &. S. Y. Oduwaye, “ A Trend Analysis of the Challenges of International Students Over 21 Years.,” *Sage Open, ,* vol. 13 , no. 4, 2023.

[61] C. M. H. S. D. &. A. D. M. Farmer, “Balancing Demand and Supply for Vetrans' Health Care: A Summary of Three RAND Assessments Conducted Under the Veterans Choice Act.,” *Rand health quarterly,* vol. 6, no. 1, 2016.

[62] M. M. M. P. &. S. I. Roshid, “Interrogating higher education's responses to international student mobility in the context of the COVID-19 pandemic,” *Heliyon,* vol. 9, no. 3, 2023.

[63] WHO, “World mental health report: Transforming mental health for all.,” WHO, 2022.

[64] G. T. G. M. &. A. Z. Engdaw, “Hand hygiene compliance and associated factors among health care providers in Central Gondar zone public primary hospitals, Northwest Ethiopia.,” *Antimicrobial resistance and infection control, ,* vol. 8, no. 190, 2019.

[65] IOM, “Annual Report for 2023,” *Council,* no. 115 Session, 2023.

[66] A. B. &. K. M. Namdar, “The Russo-Ukrainian war crisis and vaccination of Ukrainian refugees as an urgent need.,” *Vacunas,* vol. 23, no. 3, pp. 247-248, 2022.

[67] S. M. &. H. J. M. J. Hosseini Bamakan, “Role of social responsibility in prevention of the COVID-19 outbreak from systems thinking perspective.,” *Public Health,* vol. 190, 2021.

[68] S. P. P. L. S. K. A. K. C. K. T. K. S. C. P. F. G. T. &. D. J. Jaita, “International University Students' Pre-Travel Preparation, Knowledge and Practices towards Travel Health in Thailand: A Nationwide Cross-Sectional Study.,” *Tropical medicine and infectious disease,,* vol. 8, no. 6, 2023.

[69] S. D. A. G. M. F. A. M. &. S. M. Sasie, “Sasie, S. D., Ayano, G., Mamo, F., Azage, M., & Spigt, M. (Assessing the performance of the integrated disease surveillance and response systems: a systematic review of global evidence.,” *Public Public Health,* vol. 231, pp. 71-74, 2024.

[70] S. Y. P. A. M. M. Revati K Phalkey, “Revati K Phalkey, Shelby Yamamoto, Pradip Awate, MichChallenges with the implementation of an Integrated Disease Surveillance and Response (IDSR) system: systematic review of the lessons learned,,” *Health Policy and Planning,* vol. 30, no. 1, 2015.

[71] J. J.-T. C. B.-J. C. R. J. J. Y. Tenorio-Mucha, “Tenorio-Mucha, J.,Social determinants of the healthcare needs of undocumented migrants living with non-communicable diseases: a scoping review:,” *BMJ Public Health,* 2024.

[72] N. T. V. G. G. T. E. D. S. S. &. A. G. Mucci, “Migrant Workers and Psychological Health: A Systematic Review.,” *Sustainability,* vol. 12, no. 1, p. 120, 2020.

[73] A. &. M. H. Pavli, “Health problems of newly arrived migrants and refugees in Europe,,” *Journal of Travel Medicine,* vol. 24, no. 4, 2017.

[74] P. Hunter, “The refugee crisis challenges national health care systems.,” *EMBO reports. John Wiley & Sons,* vol. 17, no. 4, pp. 492-495, 2016.

[75] E. A. R. H. V. A. D. J.-A. A. S. M. a. I. N. Urbanavičė R, “Experiences and challenges of refugees from Ukraine in accessing healthcare and social services during their integration in Lithuania.,” *Frontiers in Public Health,* 2024.

[76] K. K. A. B. R. M. D. G. G. R. B. N. M. L. M. B. K. Stevenson, “Universal health coverage for undocumented migrants in the WHO European region: a long way to go,,” *The Lancet Regional Health - Europe,* 2024.

[77] A. H. S. B. H. e. a. Lebano, “Migrants’ and refugees’ health status and healthcare in Europe: a scoping literature review.,” *BMC Public Health,* vol. 20, 2020.

[78] F. G.-A. L. B. A. &. D. V. J. F. Gullo, “Crossing Countries and Crossing Ages: The Difficult Transition to Adulthood of Unaccompanied Migrant Care Leavers.,” *International journal of environmental research and public health,* vol. 18, no. 13, 20.

[79] N. L. J. &. d. M. K. Conkova, “The experience of aging and perceptions of “aging well” among older migrants in the Netherlands.,” *The Gerontologist,* vol. 60, no. 2, 2020.

[80] C. A. A. V. E. &. M. R. Hommes, “Four reasons for adopting a life course approach to health in the COVID-19 era and beyond.,” *Revista panamericana de salud publica = Pan American journal of public health,* vol. 46, 2022.

[81] S. A. H. E. B. M. V. S. H. C. S. E. L. &. H. N. Russ, “What Makes an Intervention a Life Course Intervention?.,” *Paediatrics,* vol. 149, no. Suppl 5, 2022.

[82] U. B. K. &. S. J. Bultmann, “Integrating a life course perspective in work environment and health research: empirical challenges and interdisciplinary opportunities.,” *Scand J Work Environ Health,* vol. 50, no. 5, pp. 311-316, 2024.

[83] F. a. P. I. Bernardi, “Education as an equalizer for human development?,” UNDP Human Development Report, 2019.

[84] D. P. M. &. A. C. Akiba, “Study Abroad Angst: A Literature Review on the Mental Health of International Students During COVID-19.,” *International Journal of Environmental Research and Public Health,* vol. 21, no. 12, 2024.

[85] A. M. B. S. A. J. B. B. V. P. V. B. &. V. Z. Kosztin, “Kosztin, A., Merkely, B., Szabó, A. J., Blaha,Acute SARS-CoV-2 infection and seropositivity among healthcare workers and medical students in summer.,” *International journal of occupational medicine and environmental health, 35(2), 209–216. ,* vol. 35, no. 2, pp. 209-216, 2022.

[86] P. A. U. L. P. S. P. P. E. D. I. K. M. N. S. C. I. …. K. L. R. Indrayathi, "Indrayathi, P. A., Ulandari, L. P. S., Pradnyani, P. E., Dhamanti, I., Kirshbaum, M. N., Szepesi, C. I., … Kolozsvari, L. R. (2024). Understanding International Students' Perspective of Health Service Quality: A Cross-Sectional Study in a Hungarian Univer," *Risk Management and Healthcare Policy, 17, 1757–1769,* vol. 17, pp. 1757-1769, 2024.

[87] O. J. O. C. O. A. A. E. S. A. A. e. a. F. P. t. P. A. R. o. A. P. H. P. C. A. J. o. P. H. 2. 1. (. Otorkpa, "From Policy to Practice: A Review of Africa's Public Health Policy.," *Central African Journal of Public Health,* vol. 10, no. 2, pp. 90-99, 2024.

[88] M. E. Covid-19:, "Covid-19: Bureaucracy and targets are distracting from patient care and should be suspended, say doctors;," *BMJ,* vol. 374, 2021.

[89] J. M. S. S. M. Bagaforo A. M. S., "Bagaforo Impact of COVID-19 restrictions on mental health of international students in higher education: a narrative review.," *Journal of Public Health Emerg,* vol. 8, no. 28, 2024.

[90] J. B. A. D. M. C. I. D. J. J. P. B. P. P. P. A. S. E. S. T. W. T. &. G. S. L. Kirkbride, "The social determinants of mental health and disorder: evidence, prevention and recommendations.," *World Psychiatry: official journal of the World Psychiatric Association (WPA),,* vol. 23, no. 1, pp. 58-90, 2024.

[91] H. E. a. S. M. McAvay, "'I am fine, but my group is not':Exploring the meanings of the personal/group discrimination discrepancy among minority and majority populations," *Journal of Ethnic and Migration Studies,* 2024.

[92] F. Castelli, "Drivers of migration: why do people move?,," *Journal of Travel Medicine,* vol. 25, no. 1, 2018.

[93] C. R. B. &. M. M. (. a. R. Siriwardhana, "Thematic Discussion Paper 2nd Global Consultation on Migrant Health: Resetting the agenda, Colombo, Sri Lanka.," International Organisation for Migration, 2017.

[94] A. D. M. P. A. G.-M. S. &. A. F. Pichon-Riviere, "Determining the efficiency path to universal health coverage: cost-effectiveness thresholds for 174 countries based on growth in life expectancy and health expenditures," *The Lancet. Global health, ,* vol. 11, no. 6, p. e833–e842., 2023.

[95] L. C. T. L. T. C. C. &. P. J. Chen, "The Contributions of Population Distribution, Healthcare Resourcing, and Transportation Infrastructure to Spatial Accessibility of Health Care.," *Inquiry: a journal of medical care organization, provision and financing, ,* vol. 60, 2023.

[96] G. G. Borbor, "Analysis of Health Insurance and Cash and Carry Systems in Cape Coast Teaching Hospital of Ghana. Science," *Journal of Applied Mathematics and Statistics. ,* vol. 7, no. 10, 2019.

[97] E. A. S. A. C. M. T. D. &. L. W. Liu, "Health Insurance Literacy and Medical Care Avoidance Among International Students: A Case Study.," *International journal of public health, ,* vol. 68, 2023.

[98] A. N. G.-D. B. O. P. N. N. I. N. &. R. V. K. Masai, “Healthcare services utilization among international students in Ankara, Turkey: a cross-sectional study.,” *BMC health services research, ,* vol. 2, no. 11, p. 311, 2021.

[99] Q. L. W. K. A. e. a. .. Men, “Challenges and strategies for navigating Australian healthcare access: experience from Chinese international students.,” *Int J Equity Health ,* vol. 23, p. 189, 2024.

[100] H. A. S. C. Z. H. Y. P. G. S. T. I. M. L. .. Adjei NN, “Adjei NN, Haas A, Sun CC, Zhao H, Yeh PG, Giordano SH, Toumazis I, Meyer LA.,” *Value Health.,* vol. 28, no. 2, pp. 206-214, 2025.

[101] ,. Raluca Elena Narita, “The Impact of Health Insurance,",” *JRFM, MDPI, ,* vol. 16, no. 5, pp. 1-16, 2023.

[102] N. D. D. &. R. N. Vecchio, “The effect of inadequate access to healthcare services on emergency room visits. A comparison between physical and mental health conditions.,” *PloS one, ,* 2018.

[103] Egeszsegvonal, “Social Security Code and TAJ Card,” Egeszsegvonal, 2022.

[104] T. A. N. Ibragimova E. R., “Language-related problems of international students of Elabuga Institute of Kazan Federal University.,” *Revista ESPACIOS,* vol. 39, no. 2, 2018.

[105] T. W. F.-. CIA, “Travel the globe with CIA’s World Factbook.,” The World Factbook, 2022.

[106] A. B. M. a. M. S. Darlon Jan, “What Should Be the Scope of Long-Term Care Organizations’ Obligations to Offer Culturally and Linguistically Appropriate Services to Patients?,” *AMA J Ethics. 2023;25(10):E733-739,* vol. 25, no. 10, pp. 733-739, 2023.

[107] A. H. S. P. K. H. F. R. G. K. &. H. M. F. MacFarlane, “Barriers to the use of trained interpreters in consultations with refugees in four resettlement countries: a qualitative analysis using normalisation process theory.,” *BMC family practice,* vol. 21, no. 1, 2020.

[108] M. H. A. M. F. &. W. C. M. Heath, “Interpreter services and effect on healthcare - a systematic review of the impact of different types of interpreters on patient outcome.,” *Journal of Migration and Health,* vol. 7, 2023.

[109] A. G. A. W. S. Z. E. M.-M. M. G. A. M. Y. E. C. T. W. L. D. L. X. H. B. &. B. F. Panayiotou, “Language Translation Apps in Health Care Settings: Expert Opinion,” *JMIR mHealth and uHealth, ,* vol. 7, no. 4, 2019.

[110] C. B. S. H. C. J. Rello, “Updated guidance on the management of COVID-19: from an American Thoracic Society/European Respiratory Society coordinated International Task Force,” *European Respiratory Review 2020 29,* vol. 29, no. 157, 2020.

[111] Egeszsegvonal, “Social Security Code and TAJ Card,” Egeszsegvonal, , 2022.

[112] C. G. Eleonora Trappolini, “Gendering health differences between nonmigrants and migrants by duration of stay in Italy,” *Demographic Research,* 2021.

[113] V. G. S. S.-N. A. A.-T. A. B. S.-A. M. &. K. L. R. Rekenyi, “The Effects and Differences of Social Support, Depression, and Vital Exhaustion during the COVID-19 Pandemic among International and Domestic Unive,” *International Journal of Environmental Research and Public Health,* vol. 20, no. 2, 2023.

[114] “International health insurance: what if it was easy?,” April International:.

[115] University of Pecs- International Centre-PTE, “University of Pecs,” [Online]. Available: https://international.pte.hu/current-students/information-about-services/medical-care. [Accessed 17 July 2025].

[116] P. S. A. T. e. o. t. h. s. o. i. C. a. E. E. a. t. a. w. s. e. a. p. f. a. a. o. 2. y. o. t. B. H. S. R. 1. 9. (. Romaniuk, “RThe evolution of the health system outcomes in Central and Eastern Europe and their association with social, economic and political factors: an analysis of 25 years of transition.,” *BMC Health Serv Res ,* vol. 16, p. 95, 2016.

[117] M.-K. M. B. E. R. a. a. p. o. h. a. d. a. a. C.-1. i. P. F. P. H. 2. Tuczyńska M, “Religious affiliation and perceptions of healthcare access during and after COVID-19 in Poland.,” *Front Public Health.,* 2025.
