# Supplementary material for: Knocking for gold. How long must I? A survey report on international students seeking healthcare in Hungary
Source: Front Public Health. 2026 Jan 22;13:1624806. doi: 10.3389/fpubh.2025.1624806 (PMC12872748; doi:10.3389/fpubh.2025.1624806)
Supplement: Supplementary file 4 [file Data_Sheet_4.PDF]

Figure 1

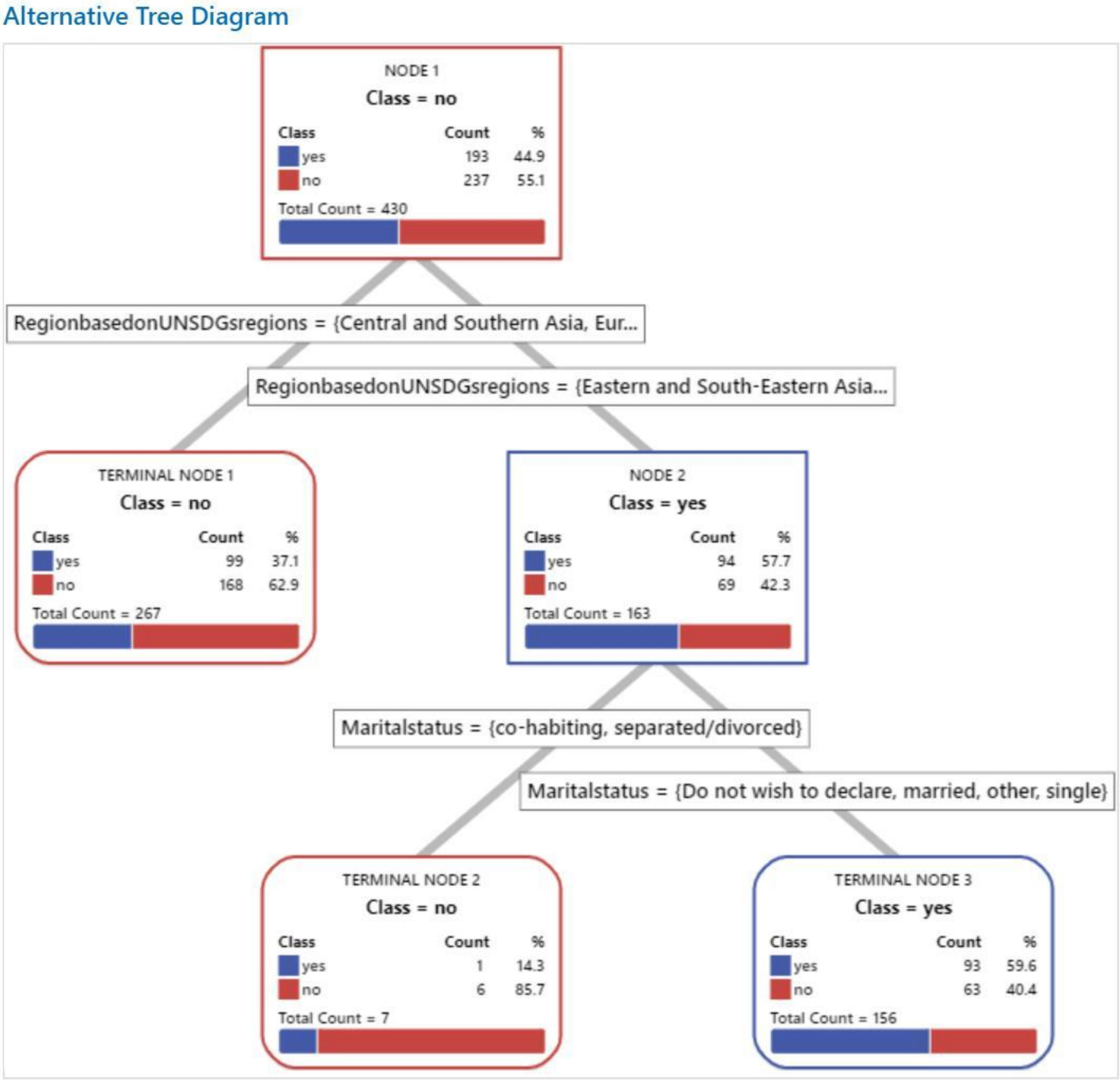

CART algorithm for worry about corona viruses

Alternative Tree Diagram

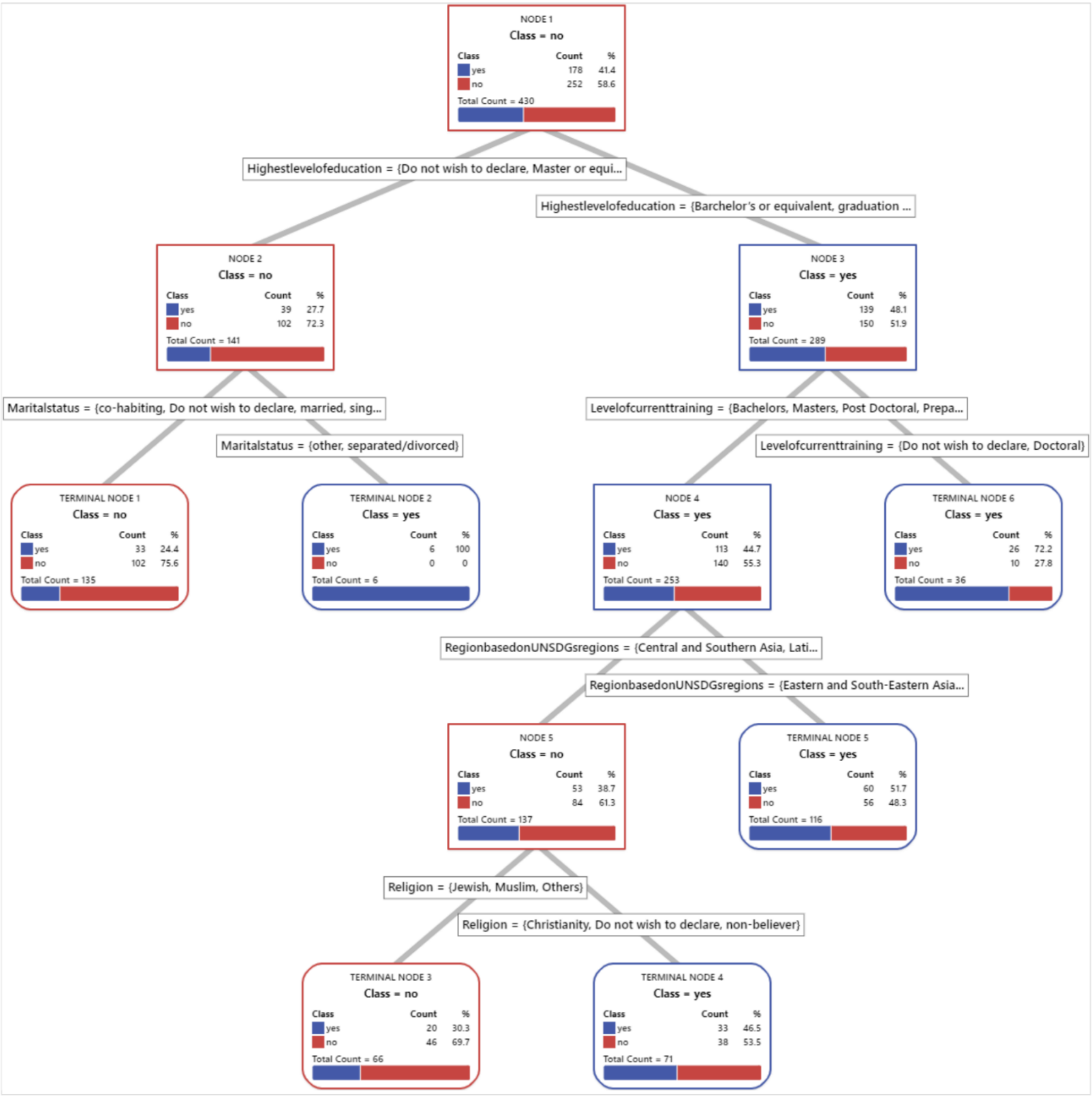

CART algorithm for worry about mental health issues

Alternative Tree Diagram

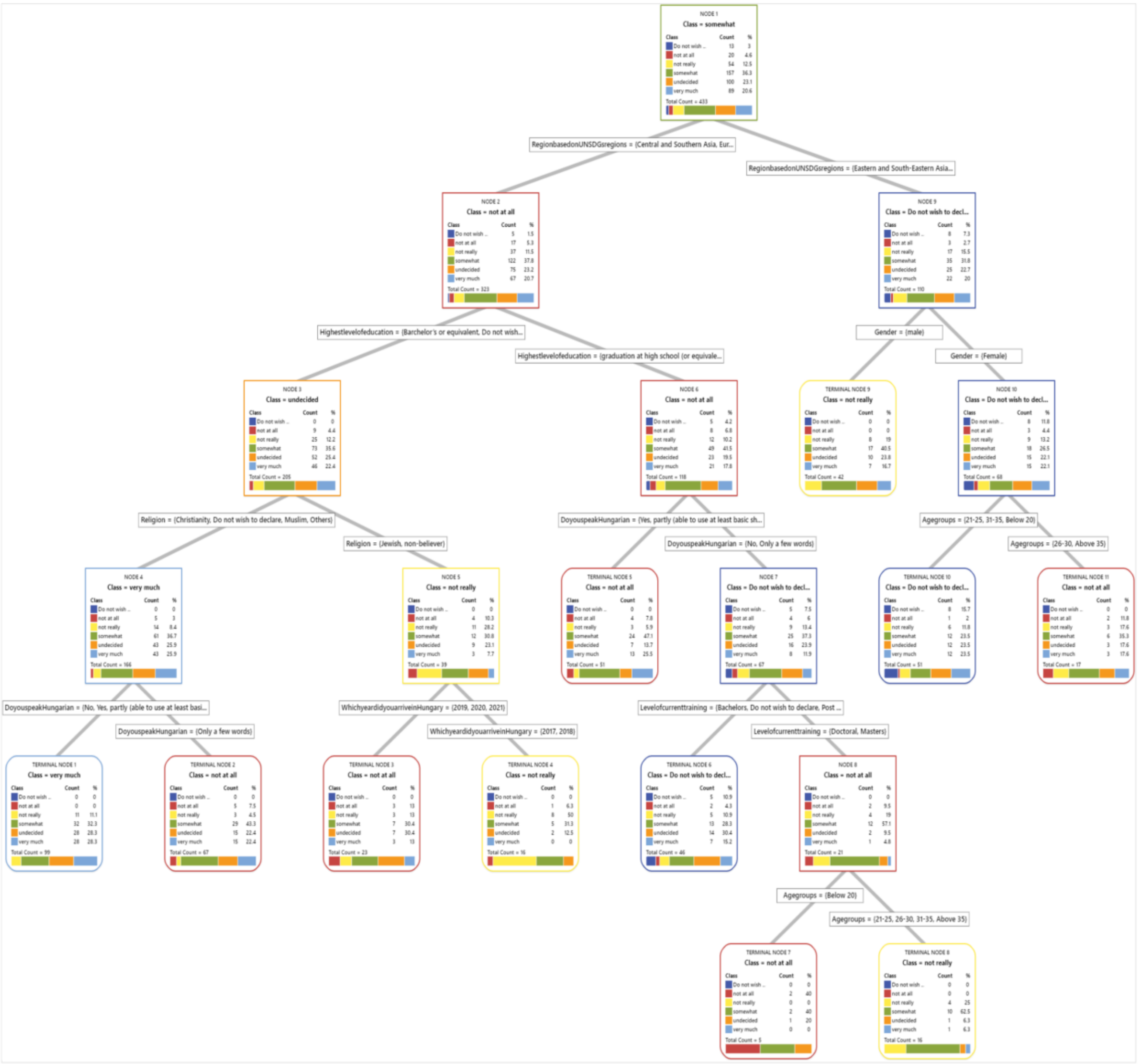

CART algorithm for trust for healthcare professionals in Hungary

Optimal Tree Diagram

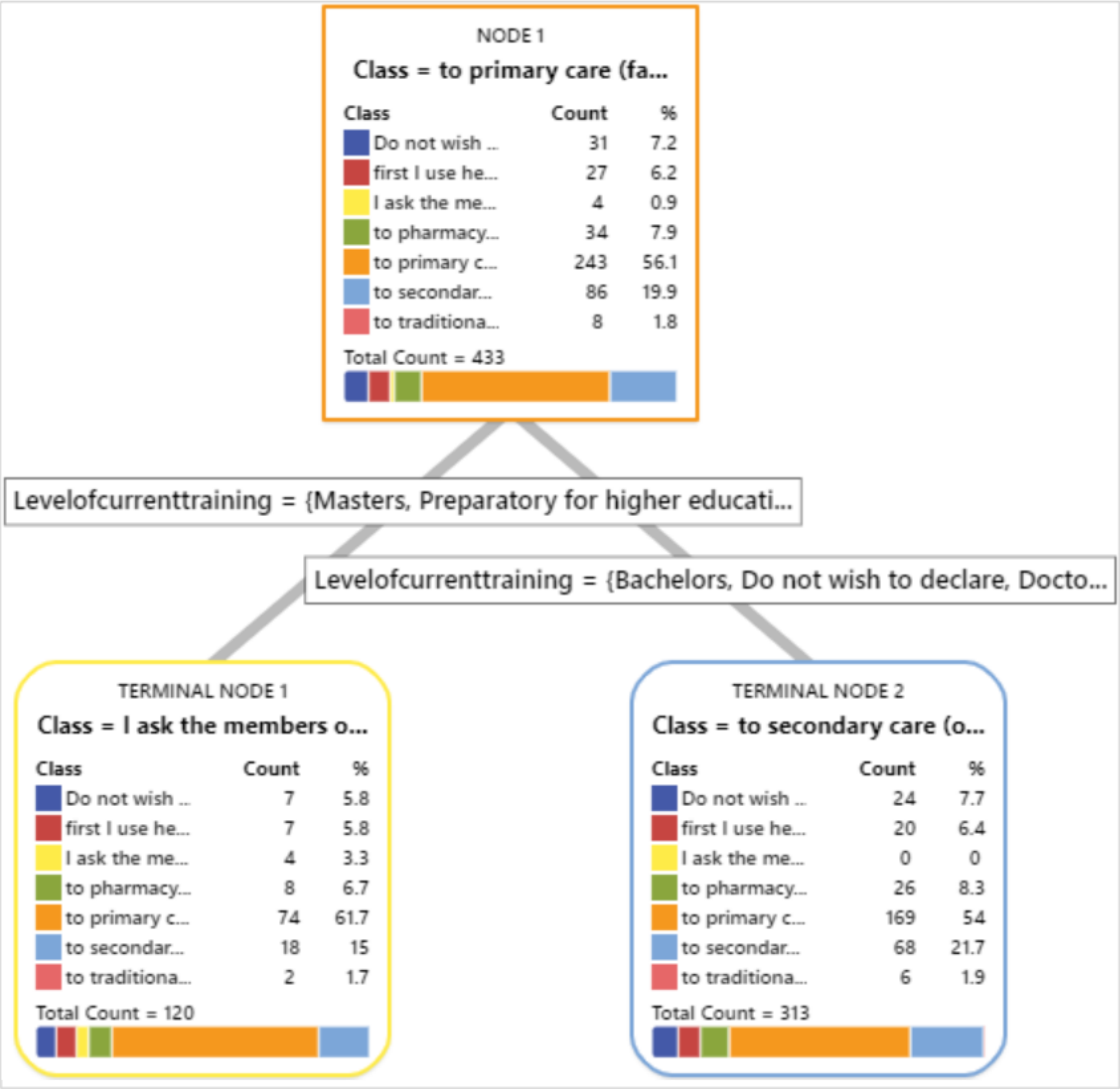

CART algorithm and first preference for healthcare services when needed

Alternative Tree Diagram

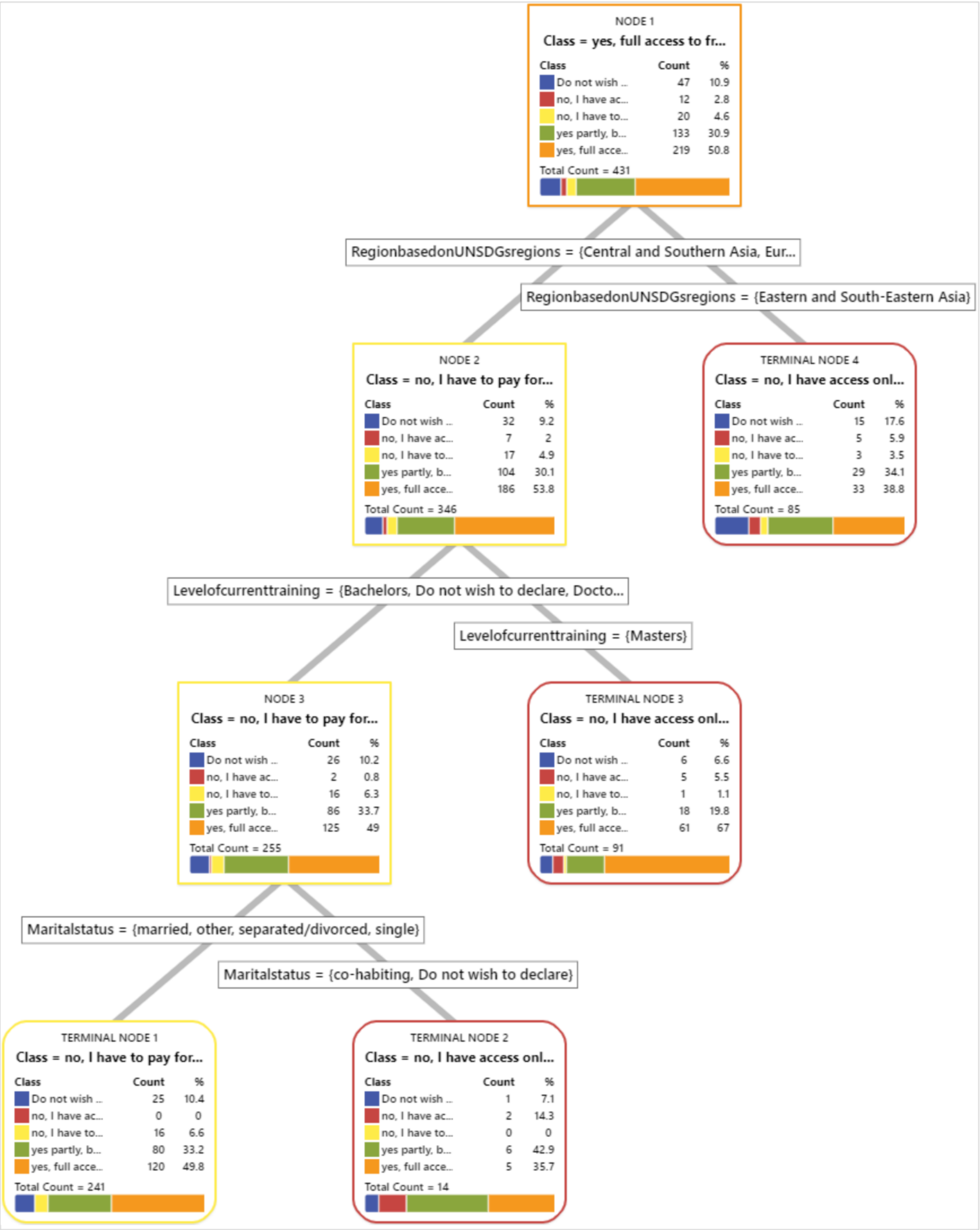

CART algorithms and access to healthcare services in Hungary

Optimal Tree Diagram

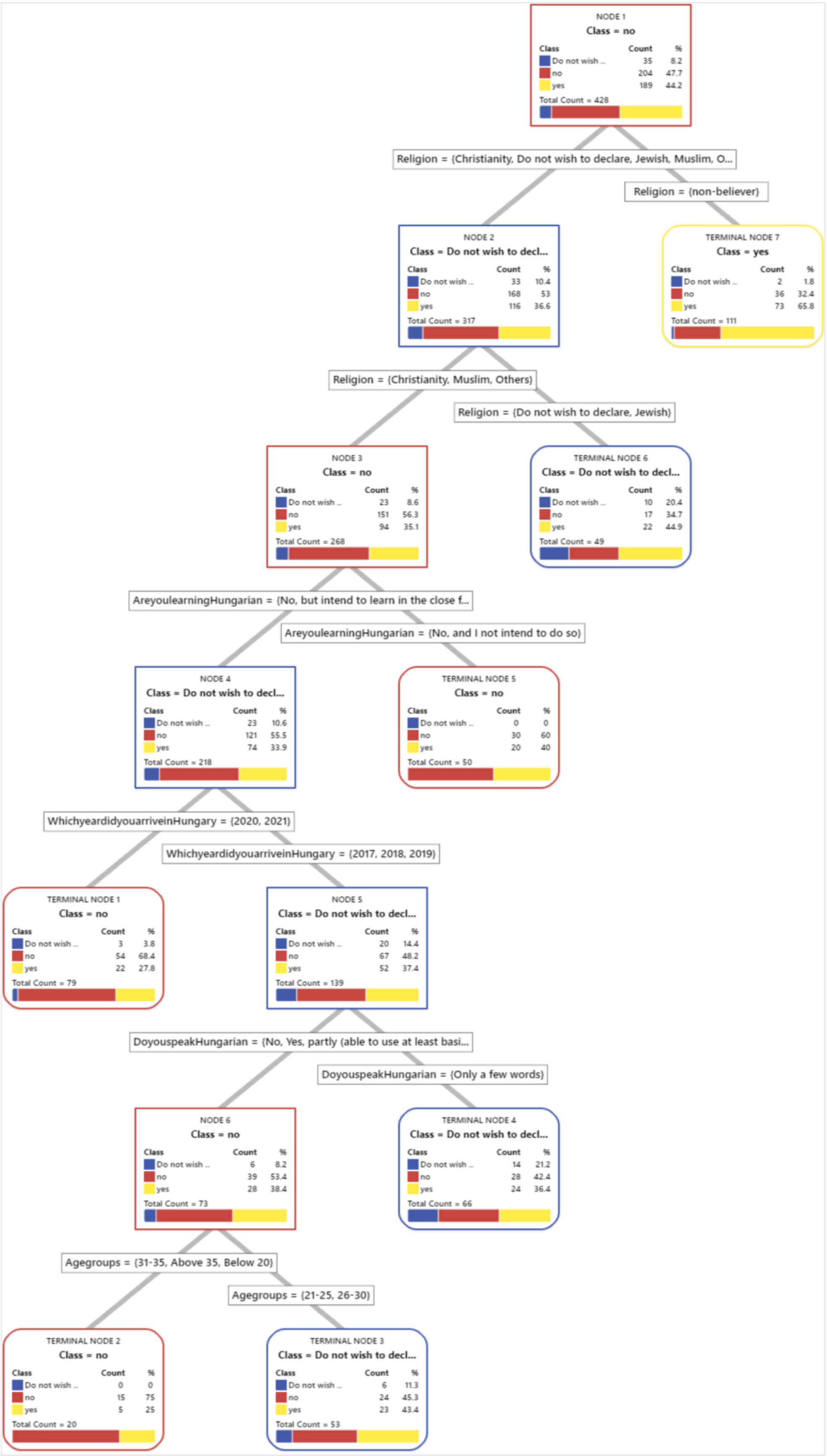

CART algorithm and the need for mental health counselling in Hungary

Alternative Tree Diagram

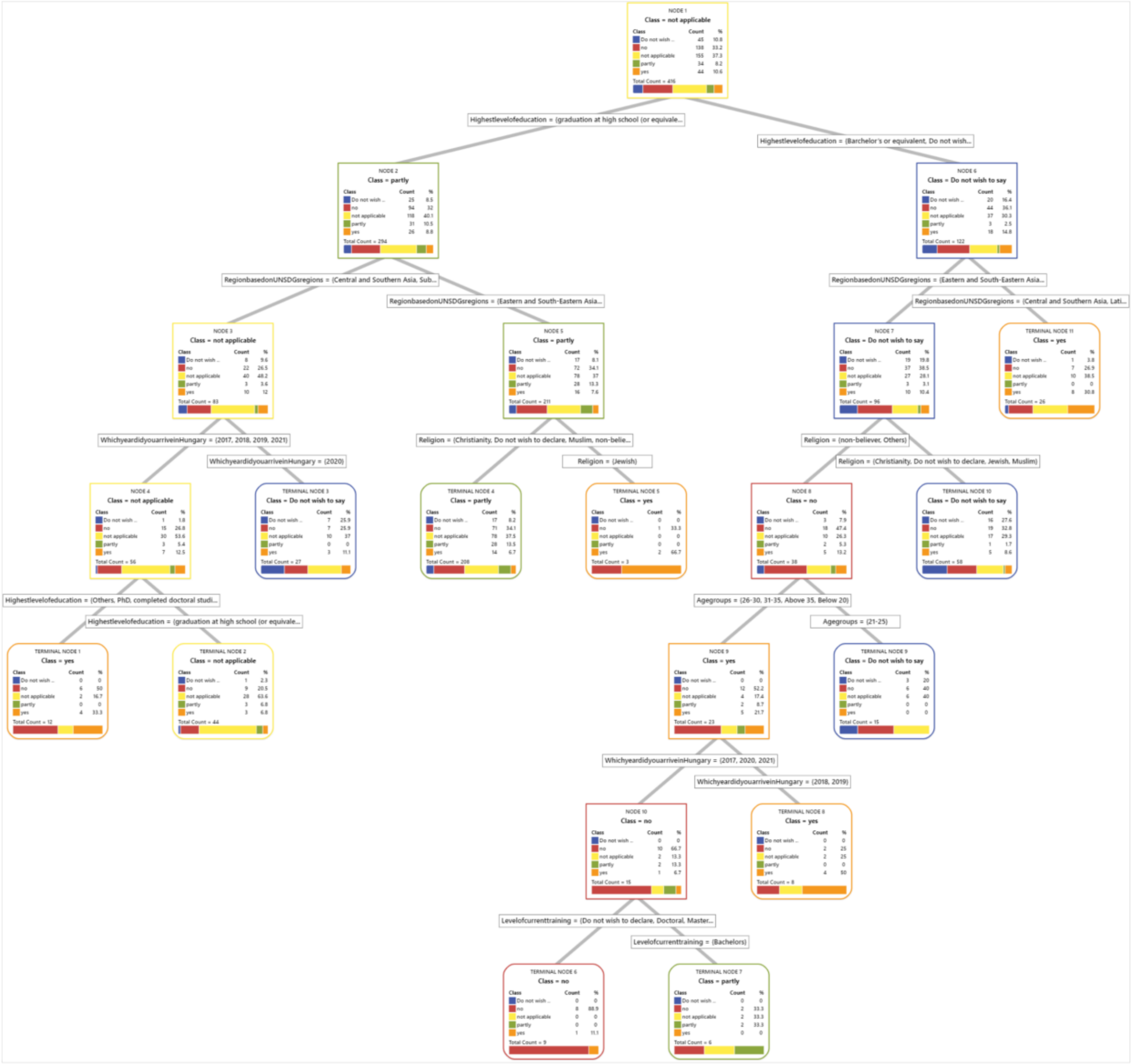

CART algorithm and getting the necessary mental healthcare

Alternative Tree Diagram

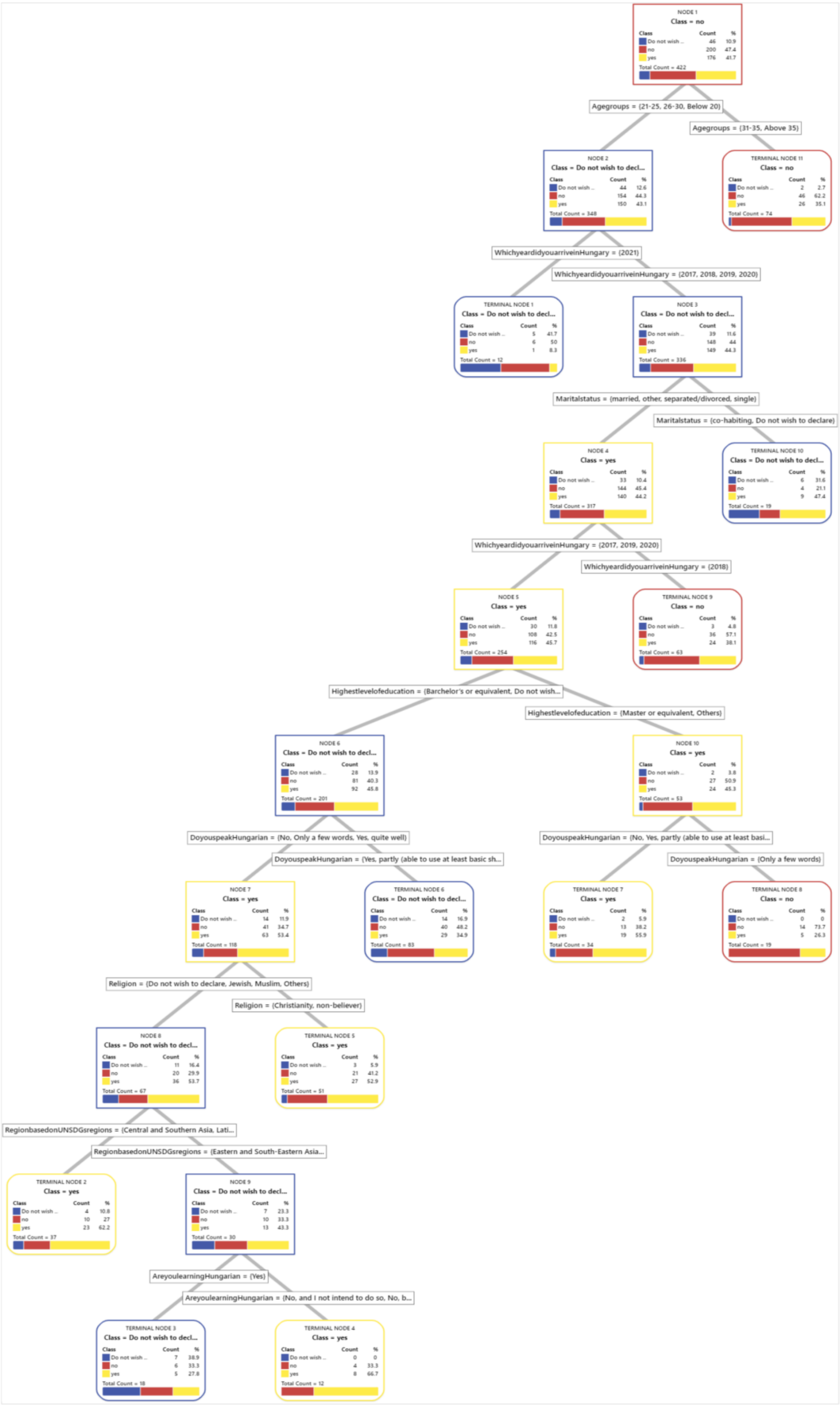

CART models and difficulties in accessing healthcare generally in Hungary
