# Supplementary material for: Knocking for gold. How long must I? A survey report on international students seeking healthcare in Hungary
Source: Front Public Health. 2026 Jan 22;13:1624806. doi: 10.3389/fpubh.2025.1624806 (PMC12872748; doi:10.3389/fpubh.2025.1624806)
Supplement: Supplementary file 5 [file Data_Sheet_5.PDF]

SOCIODEMOGRAPHICS AND CHANGE IN HEALTH AFTER ARRIVAL IN HUNGARY

**Parameters**	**Levels**	**Has your health status changed since arrival in Hungary (Counts)**					**Chi-square (χ^2)**	**p-value ($\alpha \leq 0.05$)**
		**it improved significantly**	**it improved slightly**	**It has not changed at all**	**it deteriorated slightly**	**it deteriorated significantly**		
Gender	Female	11	33	97	79	13	8.9	0.063
	male	16	36	93	44	13		
Age groups	Below 20	6	15	29	27	3	30.8	0.014
	21-25	11	35	66	63	10		
	26-30	4	11	55	18	9		
	31-35	3	7	28	11	1		
	Above 35	3	1	12	4	3		
Region based on UN SDGs regions	N/A	4	6	16	20	7	73.3	0.000
	Europe and Northern America	1	4	30	30	6		
	Northern Africa and Western Asia	6	10	56	25	5		
	Sub-Saharan Africa	2	7	21	9	0		
	Central and Southern Asia	9	17	20	8	8		
	Latin America and the Caribbean	0	2	12	9	0		
	Eastern and South-Eastern Asia	5	23	35	22	0		
Religion	Christianity	2	12	48	26	4	45.6	0.001
	non-believer	6	17	40	44	7		
	Muslim	11	20	59	27	5		
	Jewish	0	0	0	2	2		

	Others	6	15	25	10	2		
	Do not wish to declare	2	5	18	14	6		
Highest level of education	graduation at high school (or equivalent)	7	31	51	60	11	33.1	0.033
	Barchelor's or equivalent	10	18	56	23	9		
	Master or equivalent	6	14	52	27	3		
	PhD, completed doctoral studies	2	3	18	3	3		
	Others	2	1	8	5	0		
	Do not wish to declare	0	2	5	5	0		
Level of current training	Preparatory for higher education admission	1	2	0	2	0	18.5	0.557
	Bachelors	11	34	68	52	8		
	Masters	8	17	56	31	5		
	Doctoral	7	14	59	35	11		
	Post Doctoral	0	0	1	0	0		
	Do not wish to declare	0	2	6	3	2		
