# Supplementary material for: Knocking for gold. How long must I? A survey report on international students seeking healthcare in Hungary
Source: Front Public Health. 2026 Jan 22;13:1624806. doi: 10.3389/fpubh.2025.1624806 (PMC12872748; doi:10.3389/fpubh.2025.1624806)
Supplement: Supplementary file 6 [file Data_Sheet_6.PDF]

TABLE FIVE (5)

SOCIODEMOGRAPHICS AND CHANGE IN HEALTH AFTER ARRIVAL IN HUNGARY

Parameters	Levels	Has your health status changed since arrival in Hungary (Counts)					Chi-square (χ^2)	p-value ($\alpha \leq 0.05$)
		it improved significantly	it improved slightly	It has not changed at all	it deteriorated slightly	it deteriorated significantly		
Gender	Female	11	33	97	79	13	8.9	0.063
	male	16	36	93	44	13		
Age groups	Below 20	6	15	29	27	3	30.8	0.014
	21-25	11	35	66	63	10		
	26-30	4	11	55	18	9		
	31-35	3	7	28	11	1		
	Above 35	3	1	12	4	3		
Region based on UN SDGs regions	N/A	4	6	16	20	7	73.3	0.000
	Europe and Northern America	1	4	30	30	6		
	Northern Africa and Western Asia	6	10	56	25	5		
	Sub-Saharan Africa	2	7	21	9	0		
	Central and Southern Asia	9	17	20	8	8		
	Latin America and the Caribbean	0	2	12	9	0		
	Eastern and South-Eastern Asia	5	23	35	22	0		
Religion	Christianity	2	12	48	26	4	45.6	0.001
	non-believer	6	17	40	44	7		
	Muslim	11	20	59	27	5		
	Jewish	0	0	0	2	2		

	Others	6	15	25	10	2		
	Do not wish to declare	2	5	18	14	6		
Highest level of education	graduation at high school (or equivalent)	7	31	51	60	11	33.1	0.033
	Barchelor's or equivalent	10	18	56	23	9		
	Master or equivalent	6	14	52	27	3		
	PhD, completed doctoral studies	2	3	18	3	3		
	Others	2	1	8	5	0		
	Do not wish to declare	0	2	5	5	0		
Level of current training	Preparatory for higher education admission	1	2	0	2	0	18.5	0.557
	Bachelors	11	34	68	52	8		
	Masters	8	17	56	31	5		
	Doctoral	7	14	59	35	11		
	Post Doctoral	0	0	1	0	0		
	Do not wish to declare	0	2	6	3	2		
