# Supplementary material for: Knocking for gold. How long must I? A survey report on international students seeking healthcare in Hungary
Source: Front Public Health. 2026 Jan 22;13:1624806. doi: 10.3389/fpubh.2025.1624806 (PMC12872748; doi:10.3389/fpubh.2025.1624806)
Supplement: Supplementary file 7 [file Table_1.DOCX]

**SOCIODEMOGRAPHICS AND CHANGE IN HEALTH AFTER ARRIVAL IN HUNGARY**

| **Parameters** | **Levels** | **Has your health status changed since arrival in Hungary (Counts)** | | | | | **Chi-square**  **(χ²)** | **p-value**  **(α ≤ 0.05)** |
| --- | --- | --- | --- | --- | --- | --- | --- | --- |
|  |  | **it improved**  **significantly** | **it improved**  **slightly** | **It has not**  **changed at all** | **it deteriorated**  **slightly** | **it deteriorated**  **significantly** |  |  |
|  |  |  |  |  |  |  |  |  |
| Gender | Female | 11 | 33 | 97 | 79 | 13 | 8.9 | 0.063 |
|  | male | 16 | 36 | 93 | 44 | 13 |  |  |
|  |  |  |  |  |  |  |  |  |
| Age groups | Below 20 | 6 | 15 | 29 | 27 | 3 | 30.8 | 0.014 |
|  | 21-25 | 11 | 35 | 66 | 63 | 10 |  |  |
|  | 26-30 | 4 | 11 | 55 | 18 | 9 |  |  |
|  | 31-35 | 3 | 7 | 28 | 11 | 1 |  |  |
|  | Above 35 | 3 | 1 | 12 | 4 | 3 |  |  |
|  |  |  |  |  |  |  |  |  |
| Region based on UN SDGs regions | N/A | 4 | 6 | 16 | 20 | 7 | 73.3 | 0.000 |
|  | Europe and Northern America | 1 | 4 | 30 | 30 | 6 |  |  |
|  | Northern Africa and Western Asia | 6 | 10 | 56 | 25 | 5 |  |  |
|  | Sub-Saharan Africa | 2 | 7 | 21 | 9 | 0 |  |  |
|  | Central and Southern Asia | 9 | 17 | 20 | 8 | 8 |  |  |
|  | Latin America and the Caribbean | 0 | 2 | 12 | 9 | 0 |  |  |
|  | Eastern and South-Eastern Asia | 5 | 23 | 35 | 22 | 0 |  |  |
|  |  |  |  |  |  |  |  |  |
| Religion | Christianity | 2 | 12 | 48 | 26 | 4 | 45.6 | 0.001 |
|  | non-believer | 6 | 17 | 40 | 44 | 7 |  |  |
|  | Muslim | 11 | 20 | 59 | 27 | 5 |  |  |
|  | Jewish | 0 | 0 | 0 | 2 | 2 |  |  |
|  | Others | 6 | 15 | 25 | 10 | 2 |  |  |
|  | Do not wish to declare | 2 | 5 | 18 | 14 | 6 |  |  |
|  |  |  |  |  |  |  |  |  |
| Highest level of education | graduation at high school (or equivalent) | 7 | 31 | 51 | 60 | 11 | 33.1 | 0.033 |
|  | Barchelor’s or equivalent | 10 | 18 | 56 | 23 | 9 |  |  |
|  | Master or equivalent | 6 | 14 | 52 | 27 | 3 |  |  |
|  | PhD, completed doctoral studies | 2 | 3 | 18 | 3 | 3 |  |  |
|  | Others | 2 | 1 | 8 | 5 | 0 |  |  |
|  | Do not wish to declare | 0 | 2 | 5 | 5 | 0 |  |  |
|  |  |  |  |  |  |  |  |  |
| Level of current training | Preparatory for higher education admission | 1 | 2 | 0 | 2 | 0 | 18.5 | 0.557 |
|  | Bachelors | 11 | 34 | 68 | 52 | 8 |  |  |
|  | Masters | 8 | 17 | 56 | 31 | 5 |  |  |
|  | Doctoral | 7 | 14 | 59 | 35 | 11 |  |  |
|  | Post Doctoral | 0 | 0 | 1 | 0 | 0 |  |  |
|  | Do not wish to declare | 0 | 2 | 6 | 3 | 2 |  |  |
