# Supplementary material for: Knocking for gold. How long must I? A survey report on international students seeking healthcare in Hungary
Source: Front Public Health. 2026 Jan 22;13:1624806. doi: 10.3389/fpubh.2025.1624806 (PMC12872748; doi:10.3389/fpubh.2025.1624806)
Supplement: Supplementary file 9 [file Table_3.DOCX]

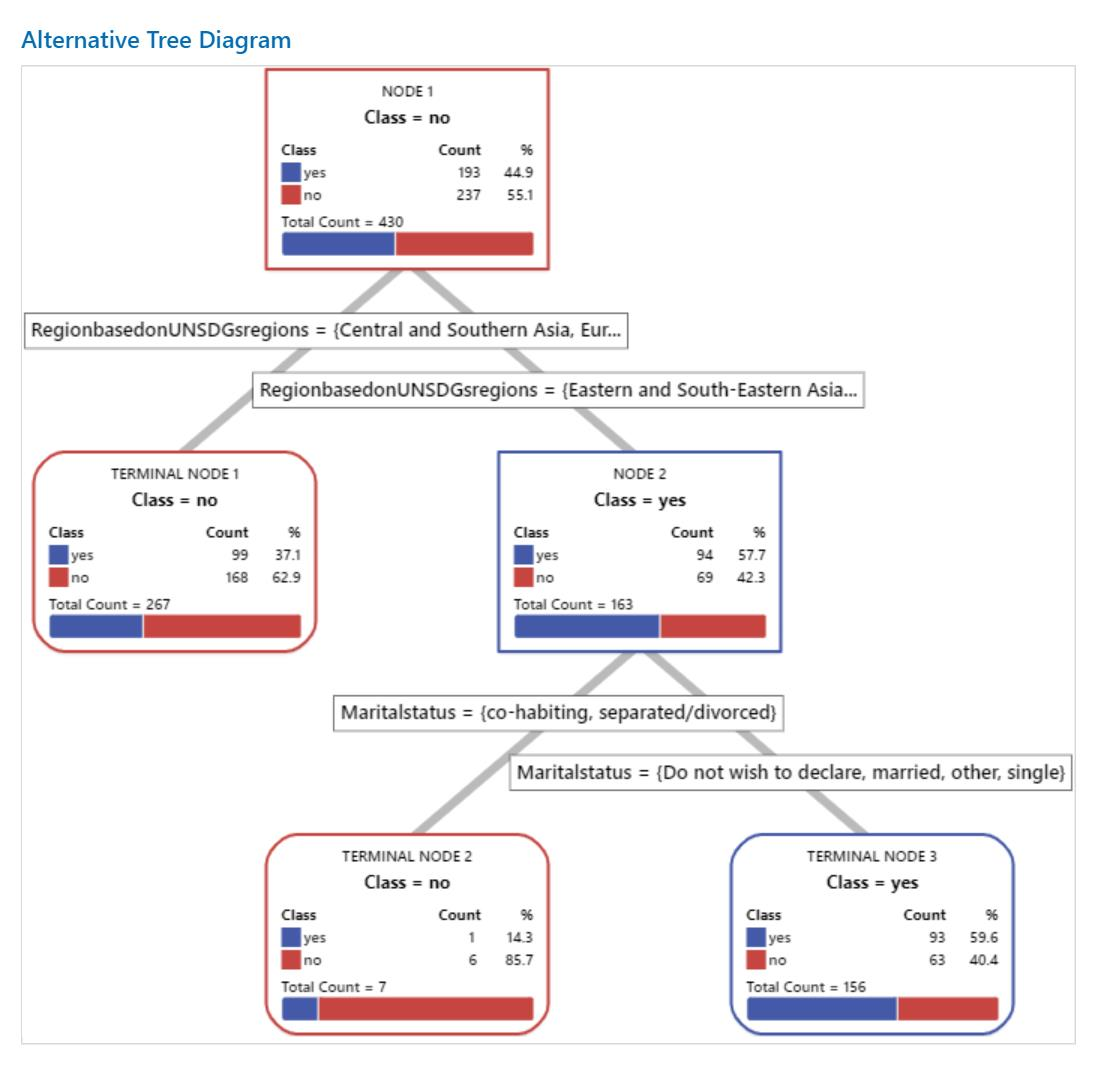


**CART algorithm for worry about corona viruses**


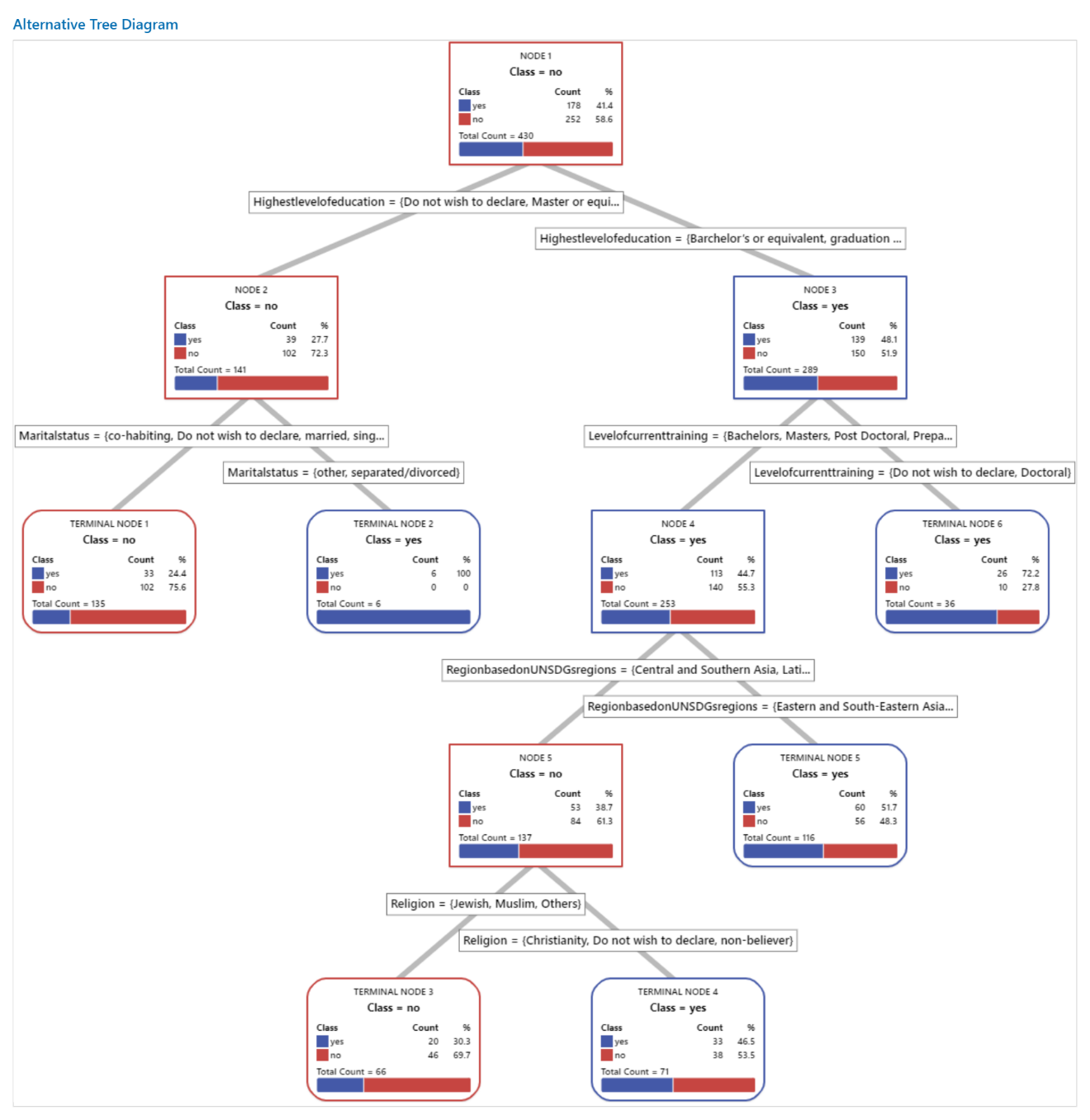


**CART algorithm for worry about mental health issues**


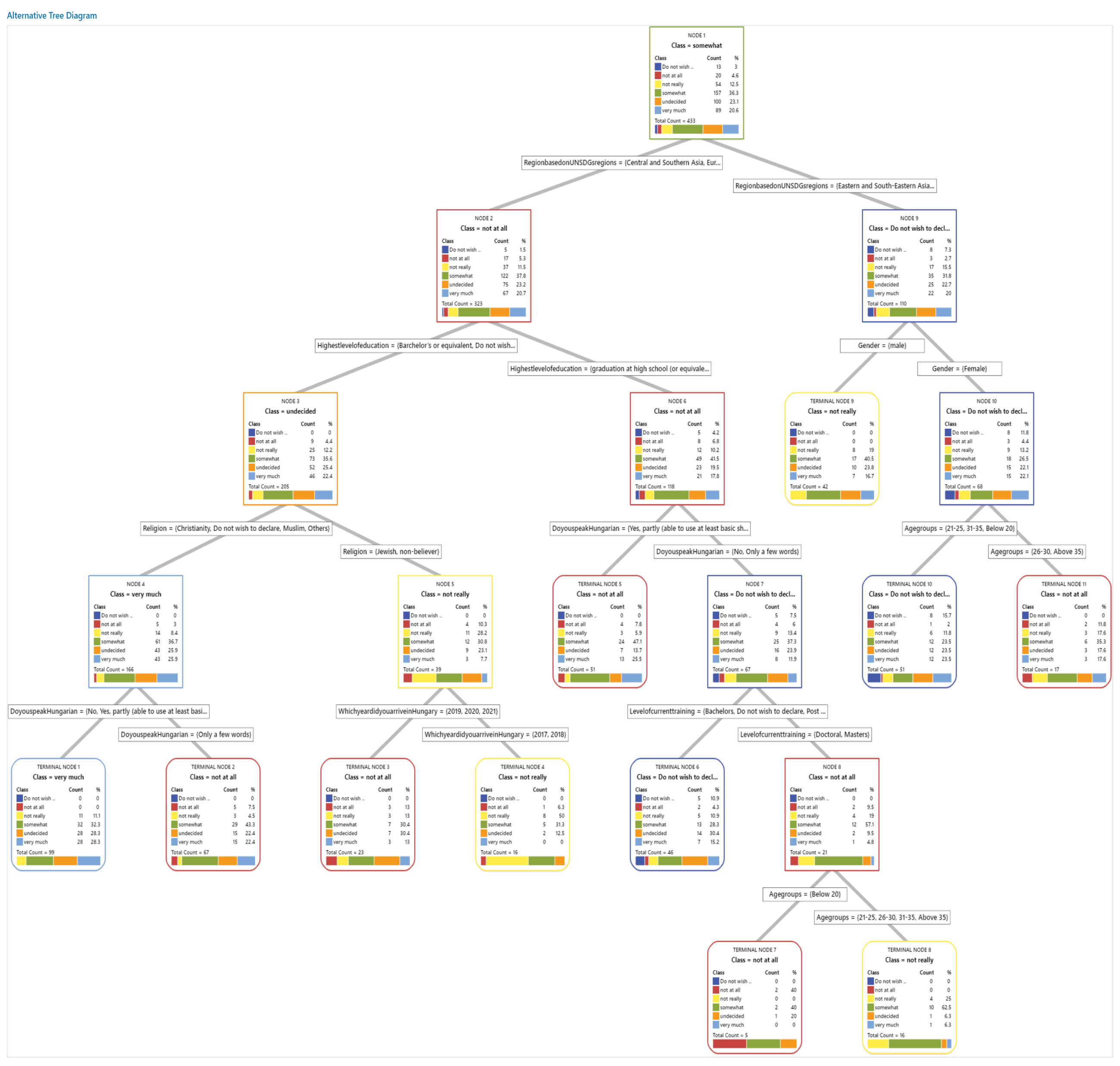


**CART algorithm for trust for healthcare professionals in Hungary**


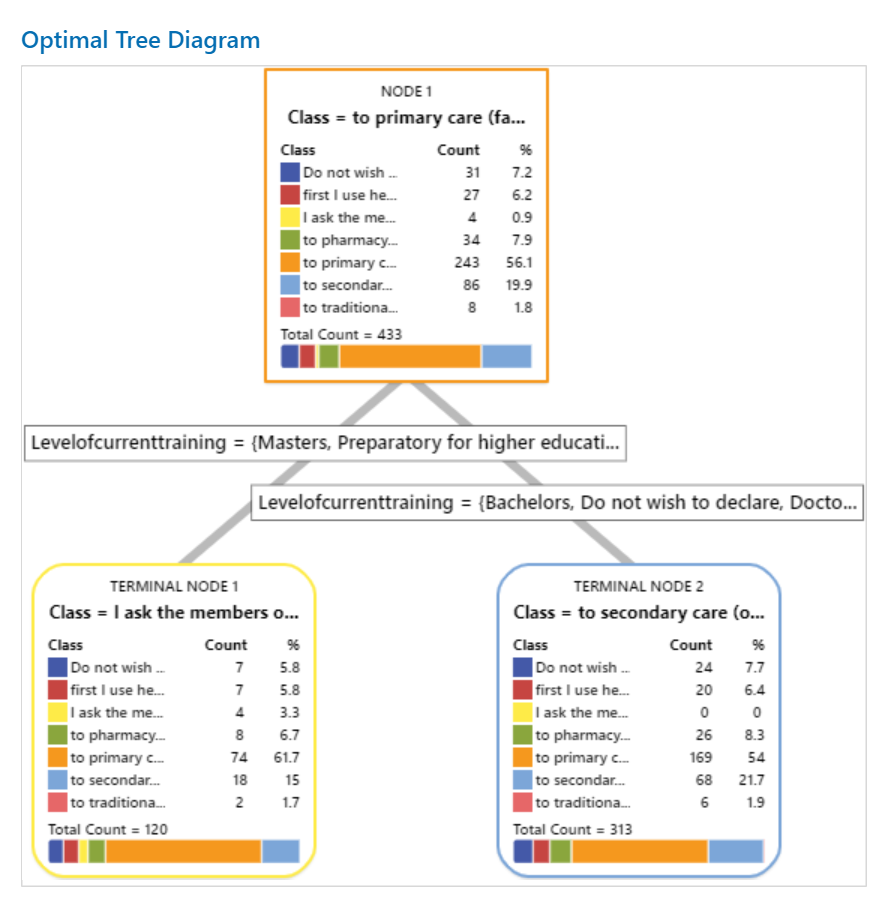


**CART algorithm and first preference for healthcare services when needed**


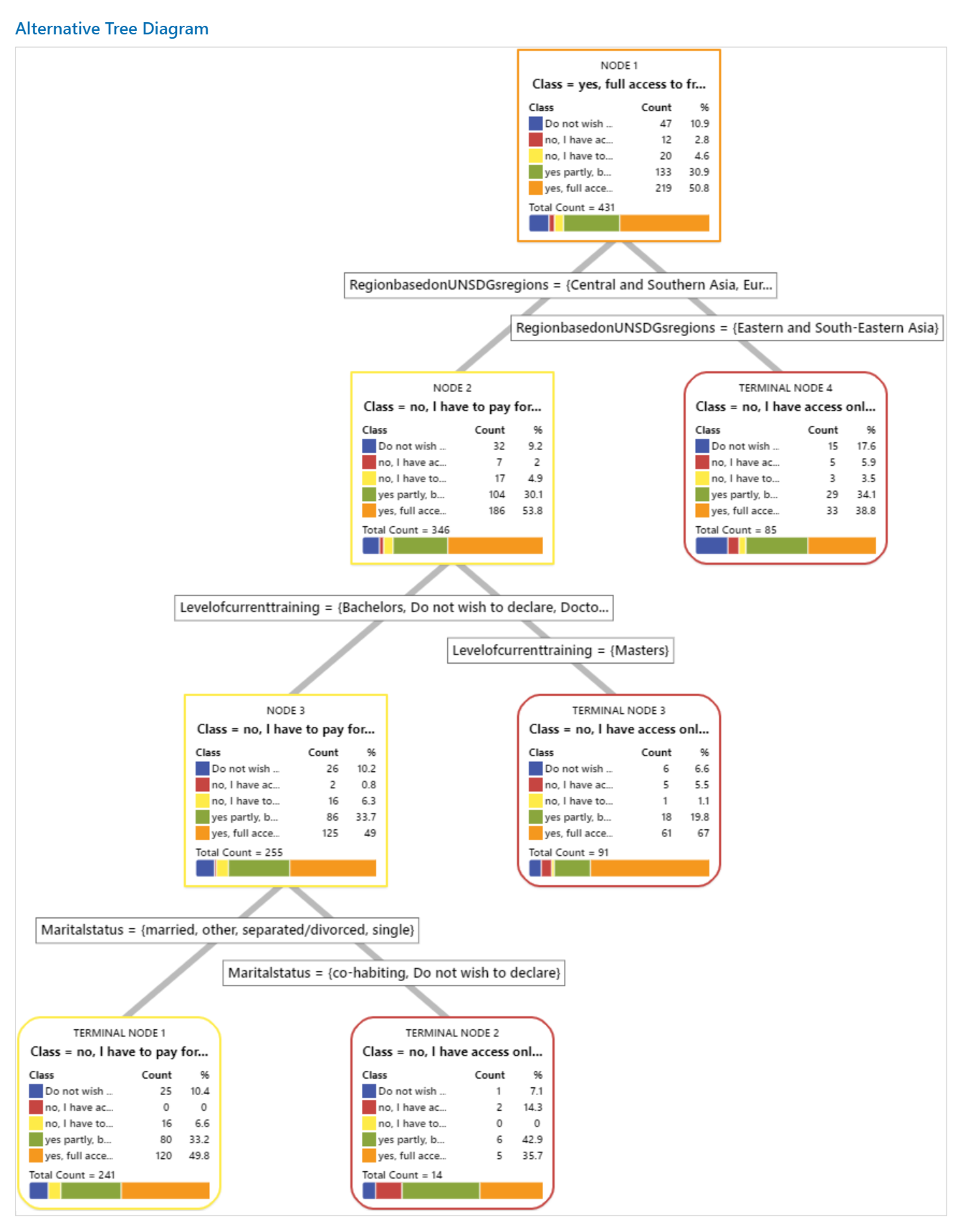


**CART algorithms and access to healthcare services in Hungary**


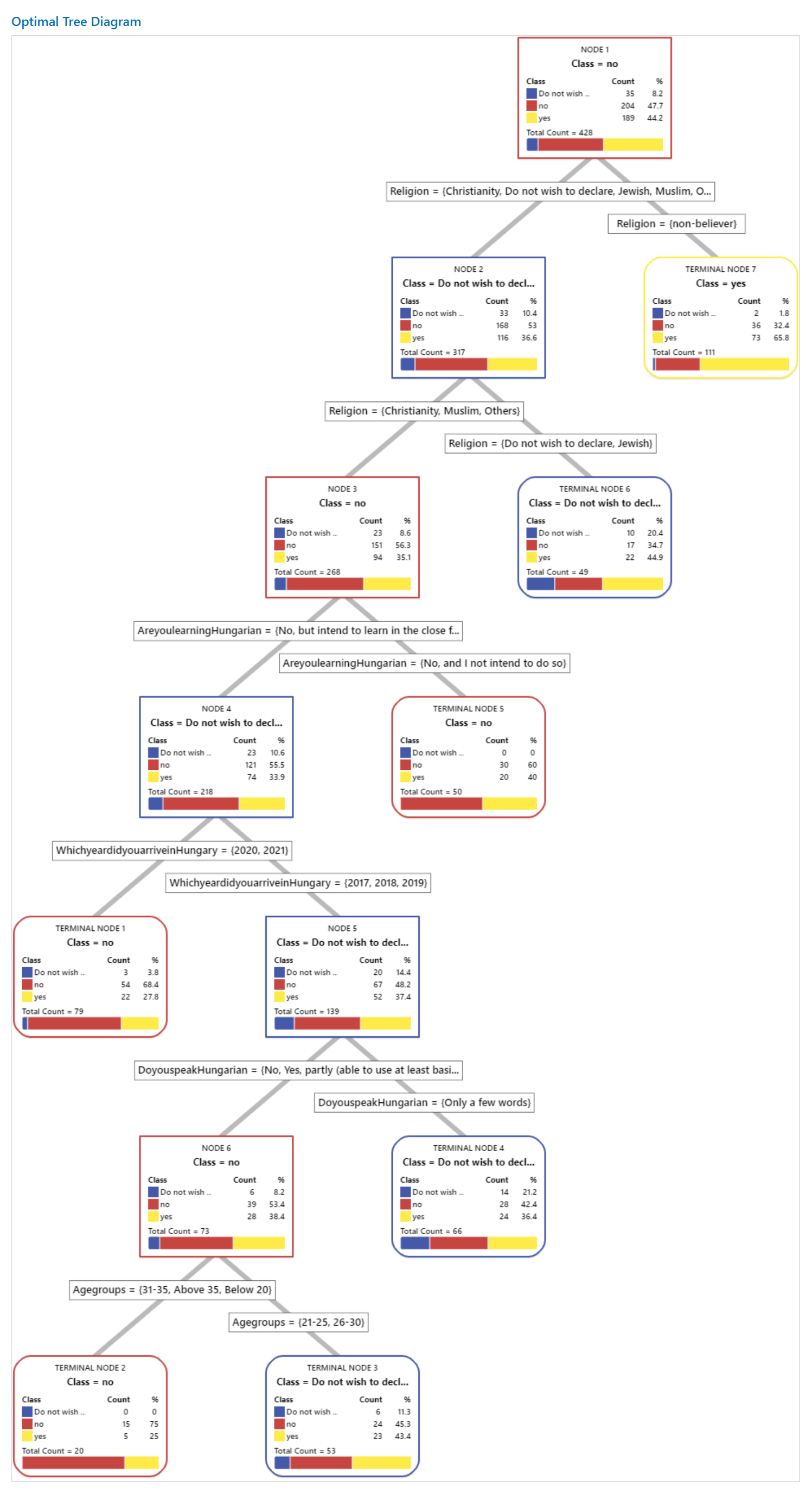


**CART algorithm and the need for mental health counselling in Hungary**


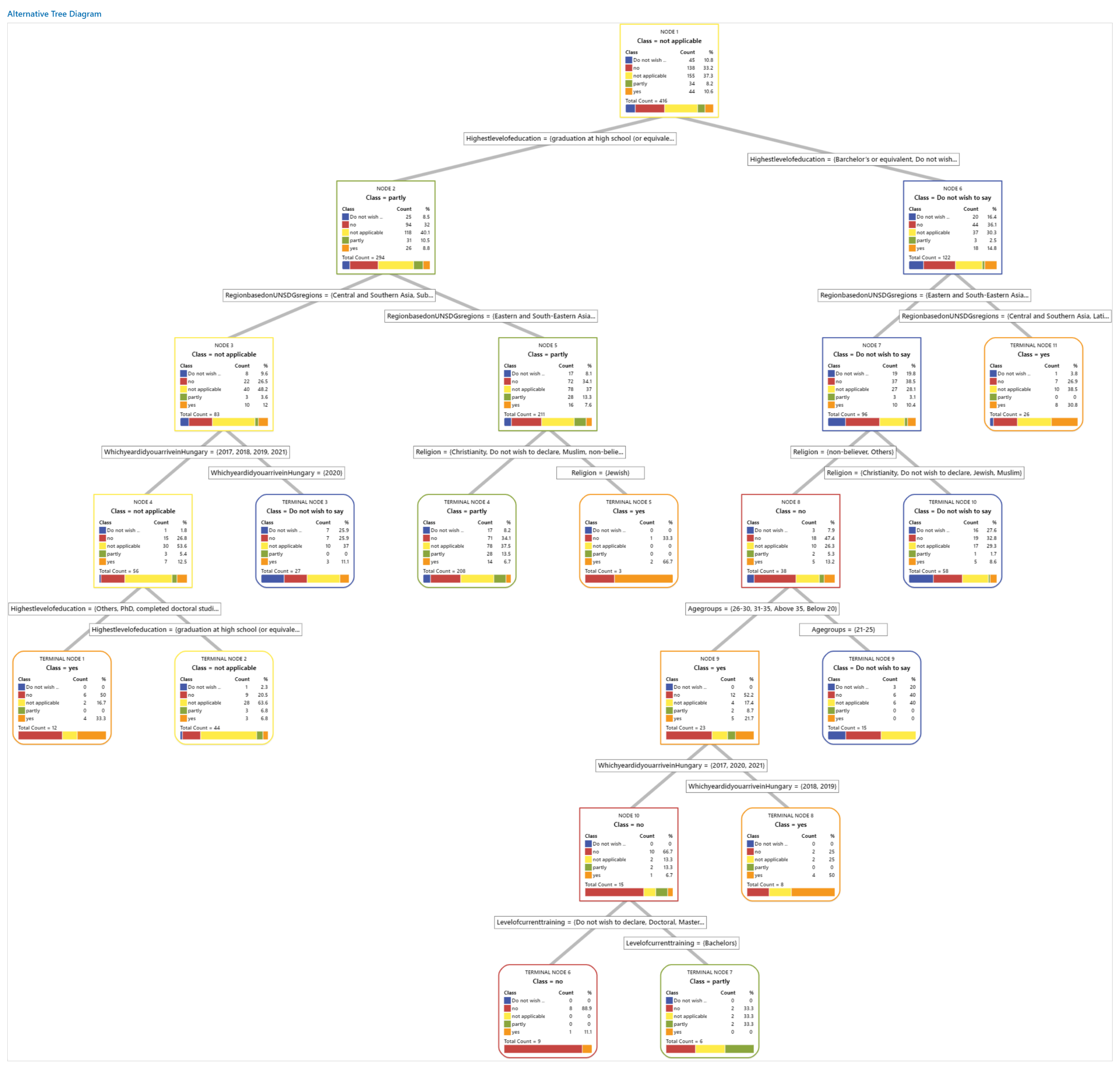


**CART algorithm and getting the necessary mental healthcare**


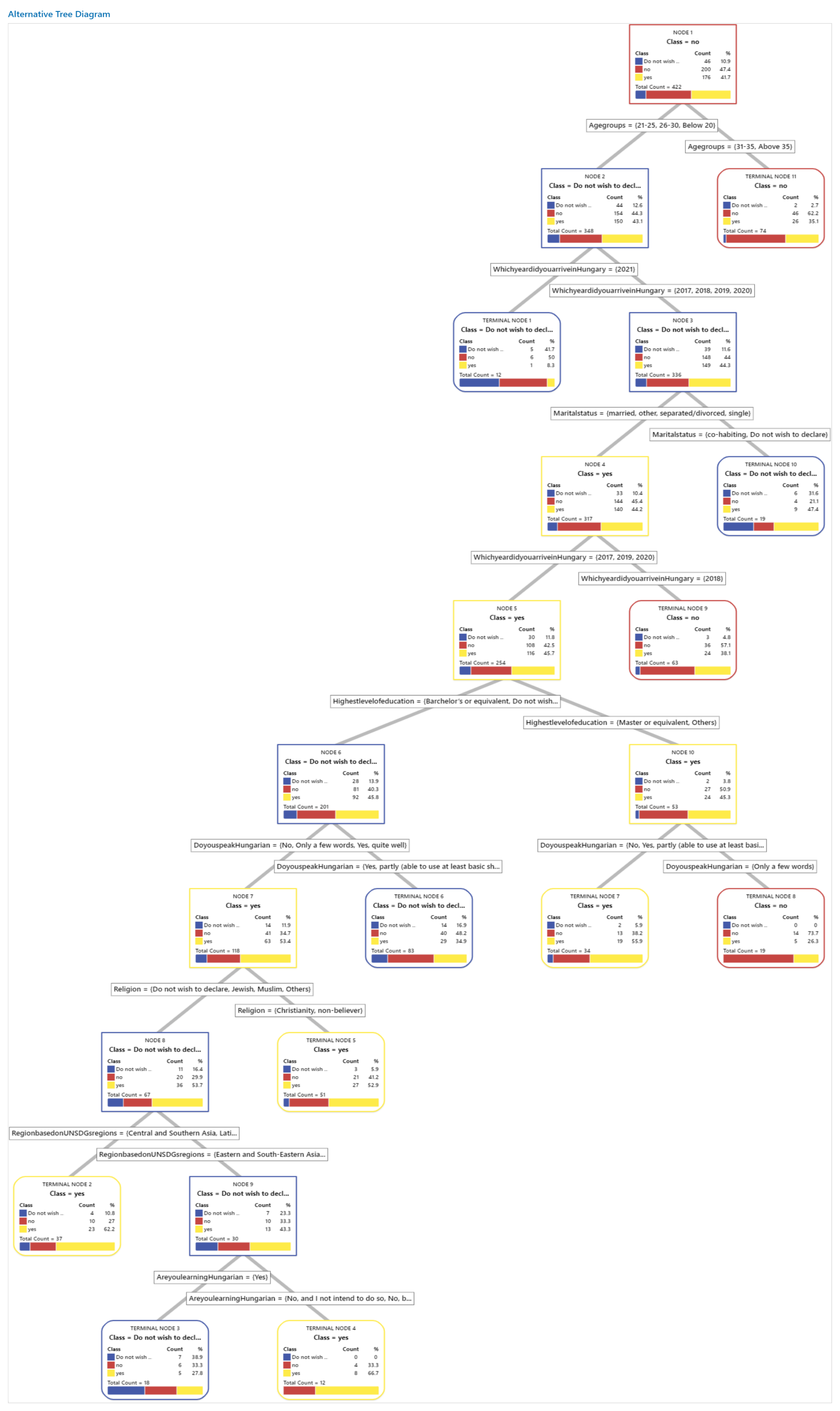


**CART models and difficulties in accessing healthcare generally in Hungary**
